# Higher Order Large Gap Asymptotics at the Hard Edge for Muttalib–Borodin Ensembles

**DOI:** 10.1007/s00220-021-04059-1

**Published:** 2021-04-29

**Authors:** Christophe Charlier, Jonatan Lenells, Julian Mauersberger

**Affiliations:** Department of Mathematics, KTH Royal Institute of Technology, Stockholm, Sweden

## Abstract

We consider the limiting process that arises at the hard edge of Muttalib–Borodin ensembles. This point process depends on $\theta > 0$ and has a kernel built out of Wright’s generalized Bessel functions. In a recent paper, Claeys, Girotti and Stivigny have established first and second order asymptotics for large gap probabilities in these ensembles. These asymptotics take the form

$$\begin{aligned} {\mathbb {P}}(\text{ gap } \text{ on } [0,s]) = C \exp \left( -a s^{2\rho } + b s^{\rho } + c \ln s \right) (1 + o(1)) \qquad \text{ as } s \rightarrow + \infty , \end{aligned}$$

where the constants $\rho$, *a*, and *b* have been derived explicitly via a differential identity in *s* and the analysis of a Riemann–Hilbert problem. Their method can be used to evaluate *c* (with more efforts), but does not allow for the evaluation of *C*. In this work, we obtain expressions for the constants *c* and *C* by employing a differential identity in $\theta$. When $\theta$ is rational, we find that *C* can be expressed in terms of Barnes’ *G*-function. We also show that the asymptotic formula can be extended to all orders in *s*.

## Introduction and Main Results

The Muttalib–Borodin ensembles are joint probability density functions of the form

1.1 $$\begin{aligned} \frac{1}{Z_{n}} \prod _{1 \le j < k \le n} (x_{k}-x_{j})(x_{k}^{\theta }-x_{j}^{\theta }) \prod _{j=1}^{n} w(x_{j})dx_{j}, \end{aligned}$$

where the *n* points $x_{1},\ldots ,x_{n}$ belong to the interval $[0,+\infty )$, $\theta > 0$ is a parameter of the model, and $Z_{n}$ is a normalization constant. The positive weight function *w* is defined on $[0,+\infty )$ and is assumed to have enough decay at $\infty$ to make (1.1) a well-defined density function.

The probability density function (1.1) exhibits so-called two-body interactions—in addition to the repulsion between the points $x_{1},\ldots ,x_{n}$, there is also repulsion between the points $x_{1}^{\theta },\ldots ,x_{n}^{\theta }$. The models defined by (1.1) were introduced by Muttalib in 1995 in the study of disordered conductors in the metallic regime [26]. They have attracted a lot of attention recently in the random matrix community, partly due to the work of Cheliotis [10] who showed that the squared singular values of certain lower triangular random matrices have the same joint density as (1.1) in the case of the Laguerre weight $w(x) = x^{\alpha }e^{-x}$, $\alpha > -1$. Other matrix ensembles whose eigenvalues are distributed according to (1.1) for the Laguerre or Jacobi weight were obtained in [18].

As $n \rightarrow + \infty$ the macroscopic behavior of the points $x_{1},\ldots ,x_{n}$ is well described by an equilibrium measure $\mu$ which depends on the weight *w*. Such measures have been studied in detail in [11] for general values of $\theta$. In particular, the authors of [11] found sufficient conditions on *w* for $\mu$ to be supported on a single cut. If there is a hard edge (that is, if part of the points accumulate near the origin as $n \rightarrow +\infty$), the density of $\mu$ behaves as a constant times $x^{-\frac{1}{1+\theta }}$ as $x \rightarrow 0^{+}$. On the other hand, near a soft edge, this density vanishes to the order 1/2 for any value of $\theta$; this is the usual square root behavior that is often encountered in random matrix theory. We also refer to [5, 8, 23] for related results on the equilibrium measure.

The Muttalib–Borodin point process is determinantal for any $\theta >0$. This means that the density (1.1), as well as all the associated correlation functions, can be expressed as determinants involving a function ${\mathbb {K}}_{n}$ (general definitions and properties of point processes can be found in [7, 21, 28]). This function ${\mathbb {K}}_{n}$ is called the kernel and encodes all the probabilistic information about the point process. In the simplest case $\theta = 1$, the point process is a polynomial ensemble. This means that all the correlation functions can be expressed in terms of orthogonal polynomials (associated to *w*), and that there exists a Christoffel–Darboux formula which can be utilized to derive asymptotic formulas as $n \rightarrow + \infty$. For $\theta \ne 1$, the point process is still determinantal; however the aforementioned properties become more complicated for rational values of $\theta$, and are lost if $\theta$ is irrational. In fact, for $\theta \ne 1$, the kernel is instead expressed in terms of biorthogonal polynomials [6], for which there is no simple analog of the Christoffel–Darboux formula (when $\theta$ is an integer, the Christoffel–Darboux formula contains $\theta$ terms, see [20]).

As $n \rightarrow + \infty$, the local repulsion of the points leads to microscopic limit laws that depend on the location. The term microscopic refers to the fact that the correlation is measured in the unit of the mean level spacing. For $\theta = 1$, three different canonical limiting kernels arise: the sine kernel arises in the bulk, the Airy kernel near soft edges, and the Bessel kernel near (typical) hard edges. The three limiting kernels are independent of the fine details of the weight; this phenomenon is called universality in random matrix theory [22]. Also, the kernels are all *integrable* (of size 2) in the sense of Its–Izergin–Korepin–Slavnov [19], and there are $2 \times 2$ matrix Riemann–Hilbert (RH) problems available for the asymptotics analysis. Much less is known for $\theta \ne 1$. In the case of the Laguerre weight $w(x) = x^{\alpha }e^{-x}$, $\alpha > -1$, Borodin proved in his pioneering work [6] that

1.2 $$\begin{aligned} \lim _{n\rightarrow + \infty } \frac{1}{n^{\frac{1}{\theta }}} {\mathbb {K}}_{n}\Big ( \frac{x}{n^{\frac{1}{\theta }}},\frac{y}{n^{\frac{1}{\theta }}} \Big ) = {\mathbb {K}}(x,y), \qquad x,y > 0, \end{aligned}$$

for any $\alpha > -1$ and $\theta > 0$, where the limiting kernel ${\mathbb {K}}(x,y)$ depends on $\alpha$ and $\theta$ and can be expressed in terms of Wright’s generalized Bessel functions (see also (1.3) below). If $\theta = p/q$ with *p*, *q* relatively prime integers, then the kernel ${\mathbb {K}}$ is integrable, but of size $p+q$ [31], which means that the associated RH problems involve matrices of size $(p+q)\times (p+q)$. In the Jacobi case (i.e., the weight is supported on [0, 1] and given by $w(x) = x^{\alpha }$, $\alpha > -1$), Borodin proved that the same limiting kernel ${\mathbb {K}}$ appears at the hard edge if a slightly different scaling limit is considered (the terms $n^{\frac{1}{\theta }}$ in (1.2) need to be replaced by $n^{1+\frac{1}{\theta }}$). It seems reasonable to expect some universality of this kernel, in the sense that ${\mathbb {K}}$ should appear in the hard edge scaling limit for a large class of weights of the form $w(x) = x^{\alpha }e^{-V(x)}$ for a smooth potential *V*. Moreover, from the behavior of $\mu$ described above, one expects the sine kernel in the bulk and the Airy kernel at the soft edge for a large class of weights. This has been proved in the special case $\theta = \frac{1}{2}$ only recently by Kuijlaars and Molag in [24] using a non-standard analysis of a $3 \times 3$ matrix RH problem. The case of general $\theta$ is still open. We also mention that, in case $\theta$ or $1/\theta$ is an integer, the kernel ${\mathbb {K}}$ can be expressed in terms of a Meijer function and coincides with the limiting kernel at the hard edge of certain product random matrices [25, Theorem 5.1].

There are several expressions available in the literature for the kernel ${\mathbb {K}}(x,y)$ in (1.2); in [6] it is written as a series, and also in terms of Wright’s generalized Bessel functions. For us, the following double contour integral expression (from [12]) will be important:

1.3 $$\begin{aligned} {\mathbb {K}}(x,y) = \frac{1}{4\pi ^{2}} \int _{\gamma } du \int _{{\tilde{\gamma }}} dv \frac{F(u)}{F(v)} \frac{x^{-u}y^{v-1}}{u-v}, \qquad x,y >0, \end{aligned}$$

where the function *F* is given by

**Fig. 1:**
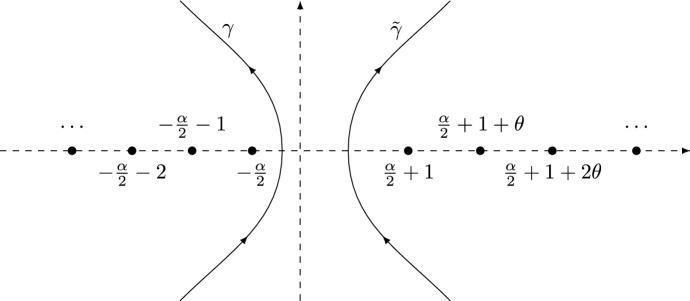
The contours $\gamma$ and ${\tilde{\gamma }}$ for $\alpha = 1.6$ and $\theta = 1.2$. The dots are the zeros and poles of *F*

1.4 $$\begin{aligned} F(z) = \frac{\Gamma \big ( z+\frac{\alpha }{2} \big )}{\Gamma \left( \frac{\frac{\alpha }{2}+1-z}{\theta } \right) } \end{aligned}$$

with $\Gamma$ denoting the Gamma function (see [27, Chapter 5]). The contours $\gamma$ and ${\tilde{\gamma }}$ are both oriented upward and do not intersect each other; the contour $\gamma$ intersects ${\mathbb {R}}$ to the right of the poles of *F* and ${\tilde{\gamma }}$ intersects ${\mathbb {R}}$ to the left of the zeros of *F*, see Fig. 1. The contour $\gamma$ tends to infinity in sectors lying strictly in the left half-plane, and ${\tilde{\gamma }}$ tends to infinity in sectors lying strictly in the right half-plane. If $\theta = 1$, the kernel ${\mathbb {K}}$ reduces to

1.5 $$\begin{aligned} \left. {\mathbb {K}}(x,y) \right| _{\theta = 1} = 4{\mathbb {K}}_{\mathrm {Be}}(4x,4y), \qquad x,y > 0, \end{aligned}$$

where ${\mathbb {K}}_{\mathrm {Be}}$ is the well-known Bessel kernel [29] given by

$$\begin{aligned} {\mathbb {K}}_{\mathrm {Be}}(x,y) = \frac{J_{\alpha }(\sqrt{x})\sqrt{y}J_{\alpha }^{\prime }(\sqrt{y})-\sqrt{x}J_{\alpha }^{\prime }(\sqrt{x})J_{\alpha }(\sqrt{y})}{2(x-y)}, \end{aligned}$$

with $J_\alpha$ the Bessel function of the first kind of order $\alpha$.

By [12, equation (1.18)], the finite *n* probability to observe a gap on $[0, n^{-\frac{1}{\theta }}s]$ converges as $n \rightarrow + \infty$ to the probability to observe a gap on [0, *s*] in the limiting process with kernel ${\mathbb {K}}$. This is a slightly stronger result than the convergence of the kernel (1.2). Let $x^{\star } := \min \{x_{1},\ldots ,x_{n}\}$ denote the smallest point. Then the limiting distribution of $x^{\star }$ is given by

1.6 $$\begin{aligned} \lim _{n\rightarrow + \infty } {\mathbb {P}}_{n}\big (n^{\frac{1}{\theta }}x^{\star }> s\big ) = \det \big (1- \left. {\mathbb {K}} \right| _{[0,s]}\big ), \qquad s > 0, \end{aligned}$$

where the right-hand side is the Fredholm determinant associated to ${\mathbb {K}}$ on the interval [0, *s*]. For $\theta = 1$, Tracy and Widom have shown in [29] that the log *s*-derivative of this Fredholm determinant solves a Painlevé V equation. In the case of $\theta$ rational, a more involved system of differential equations has been derived recently in [31].

In the case $\theta = 1$, the large gap asymptotics (i.e., the asymptotics of (1.6) as $s \rightarrow + \infty$) are known from Deift, Krasovsky and Vasilevska [15, Theorem 4] where it was shown that^1^

1.7 $$\begin{aligned} \left. \det \Big ( 1-\left. {\mathbb {K}}\right| _{[0,s]} \Big )\right| _{\theta = 1}= & {} \frac{G(1+\alpha )}{(2\pi )^{\frac{\alpha }{2}}} \exp \Big ( -s + 2 \alpha \sqrt{s} - \frac{\alpha ^{2}}{4} \ln (4s) + {{{\mathcal {O}}}}(s^{-1/2}) \Big ) \quad \nonumber \\&\text{ as } s \rightarrow +\infty , \end{aligned}$$

where *G* is Barnes’ *G*-function (see [27, Chapter 5]). The study of the general case $\theta > 0$ has been initiated by Claeys, Girotti and Stivigny in the recent paper [12]. They obtained the asymptotic formula

1.8 $$\begin{aligned} \det \Big ( \left. 1-{\mathbb {K}} \right| _{[0,s]} \Big ) = C \exp \Big ( -a s^{2\rho }+b s^{\rho }+c \ln s +{{{\mathcal {O}}}}(s^{-\rho }) \Big ) \quad \text{ as } s \rightarrow + \infty ,\qquad \end{aligned}$$

where the real constants $\rho$, *a*, and *b* are explicitly given by

1.9 $$\begin{aligned} \rho = \frac{\theta }{1+\theta }, \qquad a = \frac{1}{4}(1+\theta )^{2} \theta ^{\frac{1-3\theta }{1+\theta }}, \quad \text{ and } \quad b = \frac{1}{2} (1+\theta )(1+2\alpha - \theta ) \theta ^{-\frac{2\theta }{\theta + 1}}.\nonumber \\ \end{aligned}$$

It is quite remarkable that, even though the kernel ${\mathbb {K}}$ is known to be integrable only for rational $\theta$, they managed to obtain an asymptotic formula valid *for any* fixed $\theta > 0$ (we comment on their method below).

### Main results

The constants *c* and *C* in the large gap probability (1.8) are *multiplicative* constants. Therefore, there is no accurate description of the large gap probability without their explicit expressions. Obtaining such expressions is precisely the purpose of this paper. Our main result is the following.

#### Theorem 1.1

(Explicit expressions for *c* and *C*). For any fixed $\theta > 0$ and $\alpha > -1$, the constants *c* and *C* that appear in the asymptotic formula (1.8) are given by

1.10 $$\begin{aligned} c =&-\frac{6 \alpha ^2-6\alpha (\theta -1) +(\theta -1)^2}{12(1+\theta )}, \end{aligned}$$

1.11 $$\begin{aligned} C =&\; \frac{G ( 1+\alpha )}{(2\pi )^{\frac{\alpha }{2}}} \exp \big ( d(1,\alpha ) - d(\theta ,\alpha ) \big ) \exp \left( \frac{24 \alpha (\alpha +2)+15+3\theta + 4 \theta ^{2}}{24(1+\theta )} \ln \theta \right) \nonumber \\&\times \exp \left( \frac{6\alpha \theta - 6 \alpha (1+\alpha )-(\theta -1)^{2}}{12 \theta } \ln (1+\theta ) \right) , \end{aligned}$$

where *G* is the Barnes *G*-function and the real quantity $d(\theta ,\alpha )$ is defined by the limit

1.12 $$\begin{aligned} d(\theta ,\alpha )&= \lim _{N\rightarrow + \infty } \Bigg [ \sum _{k=1}^{N} \ln \Gamma (1+\alpha + k \theta ) - \Bigg \{\frac{\theta }{2} N^{2} \ln N + \frac{\theta (2 \ln \theta - 3)}{4}N^{2} \nonumber \\&\quad +\left( 1+\alpha + \frac{\theta -1}{2}\right) N \ln N \nonumber \\&\quad + \left( \frac{\ln (2\pi )}{2}-(1+\alpha )+\frac{1-\theta }{2} + \left( \alpha + \frac{1+\theta }{2} \right) \ln \theta \right) N \nonumber \\&\quad +\frac{1+6\alpha ^{2} + \theta (3+\theta ) + 6\alpha (1+\theta )}{12 \theta } \ln N \Bigg \} \Bigg ]. \end{aligned}$$

#### Remark 1.2

(The case $\theta = 1$). For $\theta = 1$, the expressions for the coefficients $\rho , a,b,c$, and *C* given in (1.9)–(1.11) reduce to

1.13 $$\begin{aligned} \rho = \frac{1}{2}, \qquad a = 1, \qquad b = 2\alpha , \qquad c = -\frac{\alpha ^{2}}{4}, \quad \text{ and } \quad C = \frac{2^{- \frac{\alpha ^{2}}{2}}G(1+\alpha )}{(2\pi )^{\frac{\alpha }{2}}},\qquad \end{aligned}$$

so we recover (1.7) as a special case of (1.8).

#### Remark 1.3

(The constant *d*). The constant $d =d(\theta , \alpha )$ (constant in the sense that it is independent of *s*) is defined by the limit in (1.12) in a similar way as the Euler gamma constant $\gamma _{\mathrm {E}}$, which appears in the definition of *G*, is defined by (see [27, Eq. 5.2.3])

$$\begin{aligned} \gamma _{\mathrm {E}} = \lim _{N\rightarrow + \infty } \sum _{k=1}^{N} \left( \frac{1}{k}-\ln \left( 1+\frac{1}{k} \right) \right) . \end{aligned}$$

The definition of *d* can also be compared with the following expression for the derivative of the Riemann $\zeta$-function evaluated at $-1$ (see [27, Eq. 5.17.7]):

$$\begin{aligned} \zeta '(-1) = \lim _{N \rightarrow + \infty } \Bigg [ \sum _{k=1}^{N} \ln \Gamma (k) - \Bigg \{ \frac{1}{2}N^{2}\ln N - \frac{3}{4} N^{2} + \frac{\ln (2\pi )}{2}N-\frac{1}{12}\ln (N) \Bigg \} \Bigg ]. \end{aligned}$$

In fact, comparing the above expression with the definition (1.12) of *d*, we see that^2^

$$\begin{aligned} d(1,-1) = \zeta '(-1). \end{aligned}$$

More generally, for $\theta = 1$ but any value of $\alpha > -1$, we can use the functional equation for the Barnes *G*-function to write

1.14 $$\begin{aligned} \sum _{k=1}^{N} \ln \Gamma (1+\alpha + k) = \ln G(1+\alpha + N) - \ln G(2+\alpha ). \end{aligned}$$

Using the expansion (see [27, Eq. 5.17.5])

1.15 $$\begin{aligned} \ln G(z+1) = \frac{z^{2}}{2}\ln z - \frac{3}{4}z^{2} + \frac{\ln (2\pi )}{2}z - \frac{1}{12}\ln z + \zeta '(-1) + {{{\mathcal {O}}}}(z^{-1}), \qquad z \rightarrow + \infty ,\nonumber \\ \end{aligned}$$

we conclude from (1.12) that

1.16 $$\begin{aligned}&d(1,\alpha ) = \zeta '(-1) + \frac{1+\alpha }{2}\ln (2\pi ) - \ln G(2+\alpha ) \end{aligned}$$

for $\alpha > -1$. In other words, for $\theta = 1$, $d(\theta ,\alpha )$ is expressed in terms of already known special functions evaluated at certain points. The next proposition shows that this is still the case if $\theta$ is a rational number, but then the expression becomes more complicated.

#### Proposition 1.4

(Expression for $d(\theta , \alpha )$ when $\theta = p/q$ is rational). Let $\alpha > -1$ and $\theta = p/q$ where $p,q \in {\mathbb {N}}{{\setminus }} \{0\}$. Then $d(\theta ,\alpha )$ admits the following expression:

1.17 $$\begin{aligned} d\Big (\theta = \frac{p}{q},\alpha \Big )&= pq\zeta '(-1) + \frac{p+(1+2\alpha )q}{4} \ln (2\pi )\nonumber \\&\quad -\frac{1+6\alpha ^{2} + \theta (3+\theta ) + 6\alpha (1+\theta )}{12 \theta } \ln q \nonumber \\&\quad - \sum _{k=1}^{q} \sum _{j = 1}^{p} \ln G \left( \frac{j+\alpha }{p} + \frac{k}{q} \right) . \end{aligned}$$

#### Proof

See “Appendix A”. $$\quad \square $$

Quantities such as $\zeta '(-1)$ or $G(1+\alpha )$ appear in several asymptotic formulas in random matrix theory. For example, $\zeta '(-1)$ appears in the large gap asymptotics of the Airy point process [13] and in the asymptotics of the partition function for a large class of random matrix ensembles [9, equations (1.38)–(1.40)], while the Barnes *G*-function appears in the large gap asymptotics of the Bessel point process (see 1.7) and in the asymptotics of large Toeplitz and Hankel determinants with Fisher-Hartwig singularities [9, 14]. However, despite its relatively simple definition, we have not been able to express *d* in terms of known special functions for irrational values of $\theta$.

#### Remark 1.5

(The symmetry $\theta \mapsto \frac{1}{\theta }$). By [6, page 4], the determinant on the left-hand side of (1.8) is invariant under the following changes of the parameters:

1.18 $$\begin{aligned} s \mapsto s^{\theta }, \qquad \theta \mapsto \frac{1}{\theta }, \quad \text{ and } \quad \alpha \mapsto \alpha ^{\star }:=\frac{1+\alpha }{\theta }-1. \end{aligned}$$

It follows that the coefficients $\rho$, *a*, *b*, *c*, and *C* must obey the following symmetry relations for any $\theta > 0$ and $\alpha > -1$:

1.19 $$\begin{aligned}&\rho (\theta ,\alpha ) = \theta \rho \big ( \tfrac{1}{\theta },\alpha ^{\star } \big ), \quad a(\theta ,\alpha ) = a(\tfrac{1}{\theta },\alpha ^{\star }), \quad b(\theta ,\alpha ) = b(\tfrac{1}{\theta },\alpha ^{\star }), \nonumber \\&c(\theta ,\alpha ) = \theta c(\tfrac{1}{\theta },\alpha ^{\star }), \quad C(\theta ,\alpha ) = C(\tfrac{1}{\theta },\alpha ^{\star }), \end{aligned}$$

where we have indicated the dependence of the coefficients on $\theta$ and $\alpha$ explicitly. The first four of these relations are easily verified directly from the definitions (1.9)–(1.10) by simple computations. The relation $C(\theta ,\alpha ) = C(\tfrac{1}{\theta },\alpha ^{\star })$ can also be verified directly from the definition (1.11) of *C*, but the computations are more involved. In fact, a long but straightforward computation which uses (1.16) and the functional relation $G(z+1) = \Gamma (z)G(z)$ implies that the relation $C(\theta ,\alpha ) = C(\tfrac{1}{\theta },\alpha ^{\star })$ is equivalent to the symmetry relation for *d* given in the following proposition.

#### Proposition 1.6

(Symmetry relation for *d*). The constant $d = d(\theta , \alpha )$ defined in (1.12) satisfies

1.20 $$\begin{aligned} d(\theta ,\alpha ) =&\; d \left( \frac{1}{\theta },\frac{1+\alpha }{\theta }-1 \right) + \ln \Gamma \left( \frac{1+\alpha }{\theta } \right) - \ln \Gamma \left( 1+\alpha \right) \nonumber \\&\; + \frac{13 + 6 \alpha ^{2} + \theta (\theta -3) + 6 \alpha (\theta + 3)}{12 \theta }\ln \theta \end{aligned}$$

for $$\theta > 0$$ and $$\alpha > -1$$.

#### Proof

See “Appendix B”. $$\quad \square $$

Our second main result shows that the expansion (1.8) of the Fredholm determinant of ${\mathbb {K}}$ on [0, *s*] can be extended to all orders in powers of $s^{-\rho }$ as $s \rightarrow + \infty$. More precisely, we have the following.

#### Theorem 1.7

(Asymptotics to all orders). Let $N \ge 1$ be an integer and fix $\theta > 0$ and $\alpha > -1$. As $s \rightarrow + \infty$, there exist constants $C_1,\ldots ,C_N \in {\mathbb {R}}$ such that

1.21 $$\begin{aligned} \det \Big ( \left. 1-{\mathbb {K}} \right| _{[0,s]} \Big ) = C \exp \Big ( -a s^{2\rho }+b s^{\rho }+c \ln s + \sum _{j=1}^{N}C_j s^{-j\rho } + {{{\mathcal {O}}}}\big (s^{-(N+1)\rho }\big ) \Big ),\nonumber \\ \end{aligned}$$

where ${\mathbb {K}}$ is the kernel defined in (1.3) and $\rho , a,b,c,C$ are given by (1.9)–(1.11).

### Outline of proofs

Our proof of Theorem 1.1 is based on some preliminary results from [12]. An important and remarkable ingredient of that paper (inspired by [4]) is the identity

1.22 $$\begin{aligned} \det \Big ( \left. 1-{\mathbb {K}} \right| _{[0,s]} \Big ) = \det \Big ( 1-{\mathbb {M}}_{s} \Big ), \end{aligned}$$

where the integrable kernel ${\mathbb {M}}_{s}$ of size $2 \times 2$ is given for any $\theta > 0$ by

1.23 $$\begin{aligned} {\mathbb {M}}_{s}(u,v) = \frac{\mathbf{f }(u)^{T}\mathbf{g }(v)}{u-v}, \qquad \mathbf{f }(u) = \frac{1}{2\pi i} \begin{pmatrix} \chi _{\gamma }(u) \\ s^{u} \chi _{{\tilde{\gamma }}}(u) \end{pmatrix}, \qquad \mathbf{g }(v) = \begin{pmatrix} -F(v)^{-1} \chi _{{\tilde{\gamma }}}(v) \\ s^{-v}F(v)\chi _{\gamma }(v) \end{pmatrix}\nonumber \\ \end{aligned}$$

with $\chi _{\gamma }$ and $\chi _{{\tilde{\gamma }}}$ denoting the indicator functions of $\gamma$ and ${\tilde{\gamma }}$, respectively. Using some results from [3, 4] and following the procedure developed by Its–Izergin–Korepin–Slavnov (IIKS) [19], the authors of [12] obtained a differential identity for

1.24 $$\begin{aligned} \partial _{s} \ln \det \Big ( \left. 1-{\mathbb {K}} \right| _{[0,s]} \Big ) \end{aligned}$$

in terms of the solution *Y* of a $2 \times 2$ matrix RH problem. Moreover, by performing a (non-standard) Deift/Zhou [17] steepest descent analysis of this RH problem, they computed the large *s* asymptotics of the expression in (1.24). The asymptotic formula (1.8) and the expressions (1.9) for the coefficients *a* and *b* were then obtained from the relation

1.25 $$\begin{aligned} \ln \det \Big ( \left. 1-{\mathbb {K}} \right| _{[0,s]} \Big ) = \ln \det \Big ( \left. 1-{\mathbb {K}} \right| _{[0,M]} \Big ) + \int _{M}^{s} \partial _{s'} \ln \det \Big ( \left. 1-{\mathbb {K}} \right| _{[0,s']} \Big )ds',\nonumber \\ \end{aligned}$$

where *M* is a sufficiently large but fixed constant.

In principle, the method of [12] can be employed to obtain any number of terms in the large *s* expansion of (1.24) (even though the computations become technically more involved as higher order terms are included). In particular, it is possible to compute the constant *c* by extending the expansion of (1.24) to the next order and then substituting the resulting asymptotics into the integrand of (1.25). However, the fact that the quantity

$$\begin{aligned} \ln \det \Big ( \left. 1-{\mathbb {K}} \right| _{[0,M]} \Big ) \end{aligned}$$

is an unknown constant (independent of *s*) is an essential obstacle to the computation of *C*, see also [12, Remark 1.3]. Therefore, in the present work we adopt a different approach which takes advantage of the known result for $\theta = 1$ given in (1.7).

Whereas the approach of [12] is based on a differential identity in *s*, our approach relies on a differential identity in $\theta$. More precisely, using (1.22)–(1.23) and results from [3, Section 5.1], we apply the IIKS procedure [19] to obtain a differential identity for

1.26 $$\begin{aligned} \partial _{\theta } \ln \det \Big ( \left. 1-{\mathbb {K}} \right| _{[0,s]} \Big ) \end{aligned}$$

in terms of the solution *Y* of the RH problem of [12] mentioned above (henceforth referred to as the RH problem for *Y*). By recycling the steepest descent analysis of [12], we obtain asymptotics of *Y* as $s \rightarrow + \infty$. The steepest descent analysis in [12] was performed for $\theta$ fixed, but we can easily show that the resulting asymptotic formulas are in fact valid uniformly for $\theta$ in any compact subset of $(0,+\infty )$. An integration of (1.26) from $\theta = 1$ to an arbitrary (but fixed) $\theta > 0$ then gives

1.27 $$\begin{aligned}&\left. \ln \det \Big ( \left. 1-{\mathbb {K}} \right| _{[0,s]} \Big )\right| _{\theta } \nonumber \\&\quad = \left. \ln \det \Big ( \left. 1-{\mathbb {K}} \right| _{[0,s]} \Big )\right| _{\theta =1} + \int _{1}^{\theta } \left. \partial _{\theta '} \ln \det \Big ( \left. 1-{\mathbb {K}} \right| _{[0,s]} \Big ) \right| _{\theta '}d\theta '. \end{aligned}$$

The main advantage of this approach is that the large *s* asymptotics of

1.28 $$\begin{aligned} \left. \ln \det \Big ( \left. 1-{\mathbb {K}} \right| _{[0,s]} \Big )\right| _{\theta =1} \end{aligned}$$

are known (including the constant term), see (1.7). Therefore, if we compute the asymptotics of (1.26) to sufficiently high order and substitute the resulting expansion into (1.27) (using the uniformity of this expansion with respect to $\theta$), we can obtain *C* by performing the integral with respect to $\theta '$.

#### The two cases $\theta \le 1$ and $\theta \ge 1$

The proof of Theorem 1.1 naturally splits into the two cases $\theta \in (0,1]$ and $\theta \in [1, \infty )$. Similar techniques can be used to handle both of these cases, but since they are associated with different branch cut structures, slightly different arguments are required. To avoid having to deal with two different cases, we will therefore, for simplicity, give the derivation of Theorem 1.1 only in the case $\theta \in (0,1]$ and then appeal to the symmetry (1.18) to extend the result to $\theta \in [1, \infty )$. The extension to $\theta \in [1, \infty )$ can be carried out as follows: Assuming that Theorem 1.1 holds for $\theta \in (0,1]$, the invariance of the determinant in (1.8) under the symmetry (1.18) implies that, for any $\theta \in [1, \infty )$,

$$\begin{aligned} \ln \det \Big ( \left. 1-{\mathbb {K}} \right| _{[0,s]} \Big ) =&-a(\tfrac{1}{\theta },\alpha ^{\star }) s^{2\theta \rho (\frac{1}{\theta },\alpha ^{\star })}+b(\tfrac{1}{\theta },\alpha ^{\star }) s^{\theta \rho (\frac{1}{\theta },\alpha ^{\star })}+c(\tfrac{1}{\theta },\alpha ^{\star }) \ln s \\&+ C(\tfrac{1}{\theta },\alpha ^{\star }) +{{{\mathcal {O}}}}\Big (\frac{1}{s^{\theta \rho (\frac{1}{\theta },\alpha ^{\star })}} \Big ), \qquad s \rightarrow + \infty . \end{aligned}$$

Using the symmetries in (1.19), which we recall can be verified directly from the explicit expressions for $\rho$, *a*, *b*, *c*, *C* in (1.9)–(1.11) (see Remark 1.5), the statement of Theorem 1.1 follows also for $\theta \in [1, \infty )$. A similar argument applies to Theorem 1.7. The upshot is that it is enough to prove Theorem 1.1 and Theorem 1.7 for $\theta \in (0,1]$.

#### Comparison with the approach of [12]

Even though our approach has the major advantage of opening up a path to the evaluation of the constant *C*, there are several disadvantages of integrating with respect to $\theta$ instead of with respect to *s*. First, in [12] the authors were able to obtain expressions for the constants *a* and *b* at the hard edge not only for Muttalib–Borodin ensembles, but also for certain other limiting point processes arising from products of random matrices. This was feasible because *s* is a common parameter in all of these models and the associated differential identities could be analyzed in a similar way in all cases. Since the parameter $\theta$ is not present in the other models, our method of deforming with respect to $\theta$ can only be applied in the case of the Muttalib–Borodin ensembles. Second, integration with respect to $\theta$ requires significantly more computational effort than integration with respect to *s*. This can be seen by taking the logarithm of the asymptotic formula (1.8) and differentiating the resulting equation with respect to *s* and $\theta$ respectively:^3^

1.29 $$\begin{aligned} \partial _{s} \ln \det \bigg (1 - {\mathbb {K}}\Big |_{[0,s]}\bigg ) =&- 2 \rho a s^{2\rho -1} + \rho b s^{\rho -1} + \frac{c}{s} + {{{\mathcal {O}}}}(s^{-\rho -1}),\end{aligned}$$

1.30 $$\begin{aligned} \partial _{\theta } \ln \det \bigg (1 - {\mathbb {K}}\Big |_{[0,s]}\bigg ) =&- \frac{2a}{(1+\theta )^2} s^{2\rho } \ln {s} - (\partial _\theta a)s^{2\rho } + \frac{b}{(1+\theta )^2} s^\rho \ln {s} + (\partial _\theta b) s^{\rho }\nonumber \\&+ (\partial _\theta c) \ln {s} + \frac{\partial _\theta C}{C} + {{{\mathcal {O}}}}(s^{-\rho } \ln s), \end{aligned}$$

as $s \rightarrow + \infty$. Note that the differentiation with respect to $\theta$ generates additional terms proportional to $\ln s$. Moreover, the expansion in (1.30) involves the rather complicated first-order derivatives of *a*, *b*, *c*, and *C* with respect to $\theta$. Third, it turns out that the differential identity with respect to $\theta$ is more intricate to analyze: While (1.24) is expressed in terms of the first subleading term in the expansion of *Y*(*z*) as $z \rightarrow + \infty$ (see (2.35)), the analogous representation for (1.26) involves an integral whose integrand also contains the digamma function $\psi$ (see (6.1)). The infinitely many poles of the digamma function $\psi$ (which we recall is defined as the log-derivative of $\Gamma$, see e.g. [27, Eq. 5.2.2]) complicate the analysis considerably.

For all the above reasons, we will in Sect. 5 provide an independent derivation of the expression (1.10) for *c* by employing the differential identity in *s*. This derivation is significantly shorter than the derivation based on the differential identity in $\theta$ and it can also be generalized to other point processes. In particular, from the formulas we obtain we can straightforwardly determine the constants $c^{(1)}$ and $c^{(2)}$ of [12, formula 1.20] associated with point processes at the hard edge of certain product random matrices, see Remark 5.2. Furthermore, several important aspects of this alternative derivation of (1.10) will be useful in the proofs of Theorem 1.7 and the expression (1.11) for *C*.

Finally, we note that the fact that the approach based on the differential identity in $\theta$ yields the same expressions (1.9) and (1.10) for the coefficients *a*, *b*, *c* as the approach based on the differential identity in *s* provides a nontrivial consistency check of our results.

### Organization of the paper

In Sect. 2, we introduce some notation and recall some results from [12] that are needed for our analysis. In Sects. 3 and 4 , we establish the existence of large *s* asymptotics to all orders of three functions which play a pivotal role in the RH formulation. In Sect. 5, we use these expansions to prove Theorem 1.7 and to provide a first proof of the expression (1.10) for *c*.

In Sect. 6, we derive a differential identity with respect to the parameter $\theta$. This identity expresses the $\theta$-derivative of $\ln \det ( \left. 1-{\mathbb {K}} \right| _{[0,s]})$ as the sum of four integrals which we denote by $I_1$, $I_2$, $I_{3,K}$, and $I_{4,K}$. The arguments required to obtain the large *s* asymptotics of these integrals are rather long and are presented in Sects. 7–9.

We complete the proof of Theorem 1.1 in Sect. 10 by substituting the above asymptotics into the differential identity in $\theta$ and integrating the resulting equation with respect to $\theta$. In addition to yielding the expression (1.11) for *C*, this also provides independent derivations of the expressions (1.9) and (1.10) for the coefficients *a*, *b*, and *c*.

The proofs of Propositions 1.4 and 1.6 as well as the proofs of two lemmas (Lemma 7.2 and Lemma 8.4) are presented in the four appendices.

## Preliminary Results from [12]

All the results presented in this section are taken from [12]. We use the same notation as in [12] except that we use *G* to denote Barnes’ *G*-function and ${\mathcal {G}}$ to denote the function which is denoted by *G* in [12]. We start by recalling the RH problem for *Y*, which is central for this paper.

### RH problem for Y

- $Y : {\mathbb {C}}{{\setminus }} (\gamma \cup {\tilde{\gamma }}) \rightarrow {\mathbb {C}}^{2 \times 2}$ is analytic, where $\gamma$ and ${\tilde{\gamma }}$ are the oriented contours shown in Fig. 1.
- The limits of *Y*(*z*) as *z* approaches $\gamma \cup {\tilde{\gamma }}$ from the left (+) and from the right (–) exist, are continuous on $\gamma \cup {\tilde{\gamma }}$, and are denoted by $Y_+$ and $Y_-$, respectively. Furthermore, they are related by
  $$\begin{aligned}&Y_{+}(z) = Y_{-}(z) \begin{pmatrix} 1 &{} -s^{-z}F(z) \\ 0 &{} 1 \end{pmatrix},&z \in \gamma , \\&Y_{+}(z) = Y_{-}(z) \begin{pmatrix} 1 &{} 0 \\ s^{z}F(z)^{-1} &{} 1 \end{pmatrix},&z \in {\tilde{\gamma }}, \end{aligned}$$
  where *F* is given by (1.4).
- As $z \rightarrow \infty$, *Y* admits the expansion
  $$\begin{aligned} Y(z) = I + \frac{Y_{1}}{z} + {{{\mathcal {O}}}}(z^{-2}), \end{aligned}$$
  where the $2 \times 2$ matrix $Y_{1}$ depends on *s*, $\alpha$, and $\theta$ but not on *z*.

The solution of the RH problem for *Y* exists and is unique for any choice of the parameters $s > 0$, $\theta > 0$, and $\alpha > -1$, see [12, below (1.21)].

We choose the branch for $\ln F$ such that

2.1 $$\begin{aligned} \ln F(z) = \ln \Gamma \left( z + \frac{\alpha }{2} \right) - \ln \Gamma \left( \frac{\frac{\alpha }{2}+1-z}{\theta } \right) , \end{aligned}$$

where $z \mapsto \ln \Gamma (z)$ is the log-gamma function, which has a branch cut along $(-\infty ,0]$. Therefore, $z \mapsto \ln F(z)$ has a branch cut along $(-\infty ,-\frac{\alpha }{2}]\cup [1+\frac{\alpha }{2},+\infty )$. Following [12], we introduce a new complex variable $\zeta$ by

2.2 $$\begin{aligned} z = i s^{\rho } \zeta + \tfrac{1}{2}. \end{aligned}$$

As $s^{\rho } \zeta \rightarrow \infty$, we have the asymptotics

2.3 $$\begin{aligned} \ln F(i s^{\rho } \zeta + \tfrac{1}{2}) =&\; i s^{\rho } \ln (s) \zeta + i s^{\rho } [c_{1} \zeta \ln (i\zeta ) + c_{2} \zeta \ln (-i\zeta ) + c_{3} \zeta ] \nonumber \\&+ c_{4} \ln (s) + c_{5} \ln (i\zeta ) + c_{6} \ln (-i \zeta ) + c_{7} + \frac{c_{8}}{i s^{\rho } \zeta } + {{{\mathcal {O}}}}\left( \frac{1}{s^{2 \rho }\zeta ^{2}} \right) , \end{aligned}$$

where the logarithms on the right-hand side are defined using the principal branch. The real constants $c_{1},\ldots ,c_{8}$ are computed in [12, equation (3.12)] and are given by^4^

2.4 $$\begin{aligned}&c_{1} = 1,&c_{2} = \frac{1}{\theta }, \nonumber \\&c_{3} = - \frac{\theta + 1 + \ln \theta }{\theta },&c_{4} = \frac{\theta + (\theta -1)\alpha - 1}{2(\theta +1)}, \nonumber \\&c_{5} = \frac{\alpha }{2},&c_{6} = \frac{\theta - \alpha - 1}{2 \theta }, \nonumber \\&c_{7} = - \frac{\theta - \alpha -1}{2 \theta } \ln \theta ,&c_{8} = \frac{3(1+\alpha )^{2}-7\theta -6\alpha \theta +3\alpha ^2\theta +2\theta ^2}{24\theta }. \end{aligned}$$

We also define ${\mathcal {G}}(\zeta )$ by

2.5 $$\begin{aligned} {\mathcal {G}}(\zeta ) = F(is^{\rho } \zeta + \tfrac{1}{2})e^{-is^{\rho }(\ln (s)\zeta - h(\zeta ))}, \end{aligned}$$

where

2.6 $$\begin{aligned} h(\zeta ) = -c_{1} \zeta \ln (i \zeta ) - c_{2} \zeta \ln (-i\zeta ) - c_{3} \zeta . \end{aligned}$$

The function ${\mathcal {G}}$ above is denoted by *G* in [12, equation (3.13)], while in this paper *G* denotes Barnes’ *G*-function. Note that ${\mathcal {G}}$ also depends on *s*, $\theta$ and $\alpha$, but we omit this dependence in the notation. Following [12, Section 3.3], we define $b_{1},b_{2} \in {\mathbb {C}}$ by

2.7 $$\begin{aligned} b_{2} = - \overline{b_{1}} = |b_{2}|e^{i\phi }, \qquad \phi \in \left( - \frac{\pi }{2},\frac{\pi }{2} \right) , \end{aligned}$$

with

2.8 $$\begin{aligned}&-\mathrm{Re}\,b_{1} = \mathrm{Re}\,b_{2} = 2 \left( \frac{c_{2}}{c_{1}} \right) ^{-\frac{c_{2}-c_{1}}{2(c_{2}+c_{1})}}e^{-\frac{c_{1}+c_{2}+c_{3}}{c_{1}+c_{2}}} = 2 \theta ^{\frac{3-\theta }{2(1+\theta )}} > 0, \nonumber \\&\sin \phi = \frac{c_{2}-c_{1}}{c_{2}+c_{1}} = \frac{1-\theta }{1+\theta }. \end{aligned}$$

Thus $\phi \ge 0$ for $0 < \theta \le 1$ while $\phi \le 0$ for $\theta \ge 1$. As explained in Sect. 1.2.1, it is enough to prove Theorems 1.1 and 1.7 for $\theta \in (0,1]$ thanks to the symmetry (1.18). Therefore we will henceforth restrict ourselves to the case $0 < \theta \le 1$, for which we have $\phi \in [0, \pi /2)$.

**Fig. 2:**
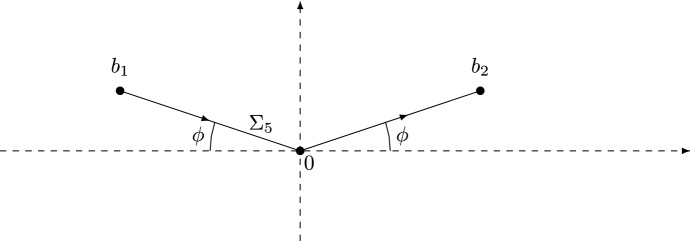
The points $b_{1}$ and $b_{2}$ lie in the upper half-plane for $0< \theta < 1$. The contour $\Sigma _{5}$ consists of the two line segments $[b_{1},0]$ and $[0,b_{2}]$

In the steepest descent analysis of the RH problem for *Y*, the so-called *g*-function plays an important role. Using this function, certain jumps of the RH problem can be made exponentially small as $s \rightarrow +\infty$. The *g*-function has a jump along the contour $\Sigma _{5}$, which consists of the two line segments $[b_{1},0]\cup [0,b_{2}]$ oriented to the right, see Fig. 2, and is defined as follows. Define the function $r(\zeta )$ by

2.9 $$\begin{aligned} r(\zeta ) = [(\zeta -b_{1})(\zeta -b_{2})]^{\frac{1}{2}}, \end{aligned}$$

where the branch is such that *r* is analytic in ${\mathbb {C}}{\setminus } \Sigma _{5}$ and $r(\zeta ) \sim \zeta$ as $\zeta \rightarrow \infty$. The second derivative of the *g*-function is given by

2.10 $$\begin{aligned} g''(\zeta ) = - i \frac{c_{1}+c_{2}}{2} \left( \frac{1}{\zeta } - \frac{1}{r(\zeta )} + \frac{i \mathrm{Im}\,b_{2}}{\zeta r(\zeta )} \right) . \end{aligned}$$

Hence

$$\begin{aligned} g_{+}''(\zeta ) + g_{-}''(\zeta ) = - i \frac{c_{1}+c_{2}}{\zeta }, \qquad \zeta \in \Sigma _{5}, \end{aligned}$$

and, as $$\zeta \rightarrow \infty $$,

$$\begin{aligned} g''(\zeta ) = \frac{2g_{1}}{\zeta ^{3}} + {{{\mathcal {O}}}}(\zeta ^{-4}), \qquad \text {where} \quad g_{1} = \frac{i (\mathrm{Re}\,b_{2})^{2}(c_{1}+c_{2})}{8}. \end{aligned}$$

The *g*-function is then obtained by

$$\begin{aligned} g'(\zeta ) = \int _{\infty }^{\zeta } g''(\xi )d\xi , \qquad g(\zeta ) = \int _{\infty }^{\zeta } g'(\xi )d\xi , \end{aligned}$$

where the integration paths lie in the complement of $\Sigma _{5}$. The *g*-function is analytic on ${\mathbb {C}} {\setminus } \Sigma _{5}$ and has the following jump across $\Sigma _5$:

2.11 $$\begin{aligned} g_{+}(\zeta ) + g_{-}(\zeta ) - i h(\zeta ) + \ell = 0, \qquad \zeta \in \Sigma _{5}, \end{aligned}$$

where $$\ell = ih(b_{1}) - 2 \int _{\infty }^{b_{1}}g'(\xi )d\xi $$.

### Steepest descent analysis

Let $\sigma _{1}$ and $\sigma _{3}$ denote the first and third Pauli matrices given by

2.12 $$\begin{aligned}&\sigma _{1} = \begin{pmatrix} 0 &{} 1 \\ 1 &{} 0 \end{pmatrix}, \qquad \sigma _{3} = \begin{pmatrix} 1 &{} 0 \\ 0 &{} -1 \end{pmatrix}. \end{aligned}$$

The steepest descent analysis of the RH problem for *Y* involves a sequence of transformations $Y \mapsto U \mapsto T \mapsto S \mapsto R$. The first transformation $Y \mapsto U$ is defined by

2.13 $$\begin{aligned} U(\zeta ) = s^{\frac{\sigma _{3}}{4}} Y \left( i s^{\rho } \zeta + \tfrac{1}{2} \right) s^{-\frac{\sigma _{3}}{4}}. \end{aligned}$$

The $2\times 2$ matrix-valued function *U* is analytic on ${\mathbb {C}}{\setminus } (\gamma _{U} \cup {\tilde{\gamma }}_{U})$, where

$$\begin{aligned} \gamma _{U} = \{\zeta \in {\mathbb {C}}: i s^{\rho } \zeta + \tfrac{1}{2} \in \gamma \}, \qquad {\tilde{\gamma }}_{U} = \{\zeta \in {\mathbb {C}}: i s^{\rho } \zeta + \tfrac{1}{2} \in {\tilde{\gamma }} \}, \end{aligned}$$

see also [12, Figure 2]. Let $\{\Sigma _i\}_1^4$ denote the contours defined by

**Fig. 3:**
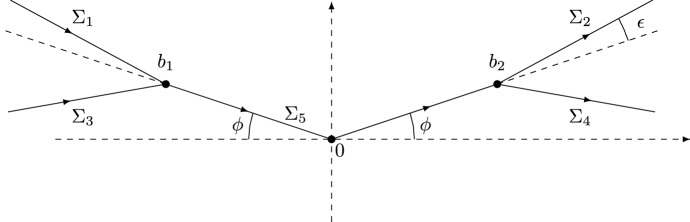
The jump contour $\cup _{i=1}^{5} \Sigma _{i}$ for the RH problem for *T*

2.14 $$\begin{aligned} \Sigma _{2} = - \overline{\Sigma _{1}} = b_{2} + e^{i(\phi + \epsilon )}{\mathbb {R}}_{\ge 0}, \qquad \Sigma _{4} = - \overline{\Sigma _{3}} = b_{2}+e^{-i \epsilon }{\mathbb {R}}_{\ge 0}, \end{aligned}$$

with $0< \epsilon < \pi /10$ and oriented from left to right, see Fig. 3. Recall that $\Sigma _{5} = [b_{1},0]\cup [0,b_{2}]$.

The second transformation $U \mapsto T$ consists of deforming the contour of the RH problem by considering an analytic continuation of *U* such that *T* is analytic in ${\mathbb {C}}{\setminus } \cup _{i=1}^{5} \Sigma _{i}$; we refer to [12, Section 3.2] for details.

The third transformation $T \mapsto S$ uses the *g*-function and is defined by

2.15 $$\begin{aligned} S(\zeta ) = e^{\frac{\ell }{2}s^{\rho }\sigma _{3}}T(\zeta ) e^{-s^{\rho }g(\zeta ) \sigma _{3}}e^{-\frac{\ell }{2}s^{\rho }\sigma _{3}}. \end{aligned}$$

The remainder of the steepest descent analysis of [12] consists of finding good approximations of *S* in different regions of the complex plane. Define the function $\gamma (\zeta )$ by

$$\begin{aligned} \gamma (\zeta ) = \left( \frac{\zeta -b_{1}}{\zeta -b_{2}} \right) ^{1/4}, \end{aligned}$$

where the branch is such that $\gamma (\zeta )$ is an analytic function of $\zeta \in {\mathbb {C}} {\setminus } \Sigma _{5}$ and $\gamma (\zeta ) \sim 1$ as $\zeta \rightarrow \infty$. Define also the function $p:{\mathbb {C}}{\setminus } \Sigma _{5} \rightarrow {\mathbb {C}}$ by

2.16 $$\begin{aligned}&p(\zeta ) = - \frac{r(\zeta )}{2\pi i} \int _{\Sigma _{5}} \frac{\ln {\mathcal {G}}(\xi )}{r_{+}(\xi )}\frac{d\xi }{\xi -\zeta }, \end{aligned}$$

where the branch for $\ln {\mathcal {G}}$ is such that

2.17 $$\begin{aligned} \ln {\mathcal {G}}(\zeta ) = \ln F(i s^{\rho } \zeta + \tfrac{1}{2}) - i s^{\rho } \left( \ln (s) \zeta - h(\zeta ) \right) \end{aligned}$$

with $\ln F$ defined as in (2.1). Outside small neighborhoods of $b_{1}$ and $b_{2}$, *S* is well approximated by the global parametrix $P^{\infty }$ defined by

2.18 $$\begin{aligned}&P^{\infty }(\zeta ) = e^{-p_{0} \sigma _{3}}Q^{\infty }(\zeta ) e^{p(\zeta )\sigma _{3}} \quad \text {with} \quad Q^{\infty }(\zeta ) = \begin{pmatrix} \frac{\gamma (\zeta )+\gamma (\zeta )^{-1}}{2} &{} \frac{\gamma (\zeta )-\gamma (\zeta )^{-1}}{2i} \\ \frac{\gamma (\zeta )-\gamma (\zeta )^{-1}}{-2i} &{} \frac{\gamma (\zeta )+\gamma (\zeta )^{-1}}{2} \end{pmatrix}. \end{aligned}$$

The function *p* satisfies $p(\zeta ) = \overline{p(-{\bar{\zeta }})}$ and

2.19 $$\begin{aligned}&p_{+}(\zeta ) + p_{-}(\zeta ) = - \ln {\mathcal {G}}(\zeta ),&\zeta \in \Sigma _{5}, \end{aligned}$$

2.20 $$\begin{aligned}&p(\zeta ) = p_{0} + \frac{p_{1}}{\zeta } + {{{\mathcal {O}}}}(\zeta ^{-2}),&\zeta \rightarrow \infty , \end{aligned}$$

where the constants $p_{0} \in {\mathbb {R}}$ and $p_{1} \in i{\mathbb {R}}$ are given by

$$\begin{aligned} p_{0}&= \frac{1}{2\pi i} \int _{\Sigma _{5}} \frac{\ln {\mathcal {G}}(\xi )}{r_{+}(\xi )}d\xi , \\ p_{1}&= - \frac{b_{1}+b_{2}}{4\pi i} \int _{\Sigma _{5}} \frac{\ln {\mathcal {G}}(\xi )}{r_{+}(\xi )}d\xi + \frac{1}{2\pi i} \int _{\Sigma _{5}} \frac{\xi \ln {\mathcal {G}}(\xi )}{r_{+}(\xi )}d\xi \nonumber \\&= \frac{1}{2\pi i} \int _{\Sigma _{5}} \frac{(\xi - i \mathrm{Im}\,(b_{2})) \ln {\mathcal {G}}(\xi )}{r_{+}(\xi )}d\xi . \end{aligned}$$

#### The local parametrix *P*

Near the points $b_{1}$ and $b_{2}$, *S* is no longer well approximated by $P^{\infty }$, and we need to construct local approximations to *S* (also called local parametrices and denoted by *P*). Following [12], these local parametrices are built out of Airy functions and are defined in small open disks ${\mathbb {D}}_{\delta }(b_1)$ and ${\mathbb {D}}_{\delta }(b_2)$ centered at $b_{1}$ and $b_{2}$, respectively:

$$\begin{aligned} {\mathbb {D}}_{\delta }(b_{j}) = \{z \in {\mathbb {C}} : |z-b_{j}|< \delta \}, \qquad j = 1,2, \end{aligned}$$

for some sufficiently small radius $\delta > 0$ which is independent of *s*. Furthermore, *P* satisfies the following matching condition with $P^{\infty }$ on the boundary $\partial {\mathbb {D}}_{\delta }(b_{1}) \cup \partial {\mathbb {D}}_{\delta }(b_{2})$:

$$\begin{aligned} e^{p_{0}\sigma _{3}}P(\zeta ) = \big (I + {{{\mathcal {O}}}}(s^{-\rho })\big ) e^{p_{0}\sigma _{3}}P^{\infty }(\zeta ), \qquad s \rightarrow + \infty , \end{aligned}$$

uniformly for $\zeta \in \partial {\mathbb {D}}_{\delta }(b_{1}) \cup \partial {\mathbb {D}}_{\delta }(b_{2})$. The local parametrix *P* obeys the symmetry

2.21 $$\begin{aligned} P(\zeta ) = \overline{P(- {\overline{\zeta }})}, \qquad \zeta \in {\mathbb {D}}_{\delta }(b_{1}) \cup {\mathbb {D}}_{\delta }(b_{2}), \end{aligned}$$

and therefore we can restrict attention to the construction of *P* in ${\mathbb {D}}_{\delta }(b_{1})$. There are a few minor typos in [12]: the factors $\sqrt{2\pi }$ in [12, equations (3.57)–(3.59)] should be $2 \sqrt{\pi }$ and the signs of the exponential factors in [12, equations (3.63), (3.65), (3.67)] should be modified. These typos have no repercussion on the results of [12], but will play a role for us. In what follows, we therefore give the definition of *P* in detail. First, define the complex-valued functions $\{y_{j}(\zeta )\}_1^3$ by

$$\begin{aligned} y_{j}(\zeta ) = e^{\frac{2\pi i j}{3}} \text{ Ai }(e^{\frac{2\pi i j}{3}}\zeta ), \qquad j = 0,1,2, \end{aligned}$$

and let the $2\times 2$-matrix valued functions $\{A_j(\zeta )\}_1^3$ be given by

2.22 $$\begin{aligned}&A_{1}(\zeta ) = -2 i \sqrt{\pi } \begin{pmatrix} -y_{2}(\zeta ) &{} -y_{0}(\zeta ) \\ -y_{2}'(\zeta ) &{} -y_{0}'(\zeta ) \end{pmatrix}, \end{aligned}$$

2.23 $$\begin{aligned}&A_{2}(\zeta ) = -2 i \sqrt{\pi } \begin{pmatrix} -y_{2}(\zeta ) &{} y_{1}(\zeta ) \\ -y_{2}'(\zeta ) &{} y_{1}'(\zeta ) \end{pmatrix}, \end{aligned}$$

2.24 $$\begin{aligned}&A_{3}(\zeta ) = -2 i \sqrt{\pi } \begin{pmatrix} y_{0}(\zeta ) &{} y_{1}(\zeta ) \\ y_{0}'(\zeta ) &{} y_{1}'(\zeta ) \end{pmatrix}. \end{aligned}$$

These functions satisfy

$$\begin{aligned}&A_{1}(\zeta ) = A_{2}(\zeta ) \begin{pmatrix} 1 &{} -1 \\ 0 &{} 1 \end{pmatrix}, \qquad A_{2}(\zeta ) = A_{3}(\zeta ) \begin{pmatrix} 1 &{} 0 \\ 1 &{} 1 \end{pmatrix}, \qquad A_{1}(\zeta ) = A_{3}(\zeta ) \begin{pmatrix} 1 &{} -1 \\ 1 &{} 0 \end{pmatrix}. \end{aligned}$$

Moreover,

2.25 $$\begin{aligned} A_{k}(\zeta ) = \zeta ^{-\frac{\sigma _{3}}{4}} \begin{pmatrix} 1 &{} i \\ 1 &{} -i \end{pmatrix} \Big [I + {{{\mathcal {O}}}}(\zeta ^{-3/2}) \Big ]e^{\frac{2}{3}\zeta ^{3/2}\sigma _{3}} \end{aligned}$$

as $\zeta \rightarrow \infty$ in the sector $S_{k}$ for $k = 1,2,3$, with

2.26 $$\begin{aligned} S_{k} = \left\{ \zeta \in {\mathbb {C}}: \frac{2k - 3}{3}\pi + \delta \le \arg \zeta \le \frac{2k+1}{3}\pi - \delta \right\} , \qquad k = 1,2,3, \end{aligned}$$

and the branches of the complex powers in (2.25) are such that $\zeta ^u = e^{u\ln |\zeta | + iu \arg \zeta }$ where $\arg \zeta$ belongs to $(-\pi /3,\pi )$, $(\pi /3,5\pi /3)$, and $(\pi ,7\pi /3)$ for $\zeta$ in $S_1, S_2, S_3$, respectively. The local parametrix *P* is defined for $\zeta \in {\mathbb {D}}_{\delta }(b_{1}) {\setminus } \cup _{i=1}^{5} \Sigma _{i}$ by

**Fig. 4:**
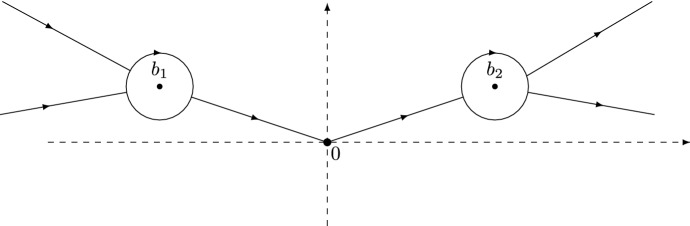
The contour $\Gamma _{R}$ for the RH problem *R*

2.27 $$\begin{aligned} P(\zeta ) = E(\zeta )A_{k} \big (s^{\frac{2}{3}\rho }f(\zeta )\big ) e^{-s^{\rho }q(\zeta ) \sigma _{3}} {\mathcal {G}}(\zeta )^{-\frac{\sigma _{3}}{2}}, \qquad \zeta \in [k], \;\; k = 1,2,3, \end{aligned}$$

where [*k*], $k = 1,2,3$, denote the three components of ${\mathbb {D}}_{\delta }(b_{1}) {\setminus } \cup _{i=1}^{5} \Sigma _{i}$ as shown in [12, Figure 4], *q* is the analytic function on ${\mathbb {D}}_\delta (b_{1}){\setminus } \Sigma _{5}$ given by

2.28 $$\begin{aligned} q(\zeta ) = g(\zeta ) - \frac{i}{2}h(\zeta ) + \frac{\ell }{2}, \end{aligned}$$

the function *f* is defined by

2.29 $$\begin{aligned} f(\zeta ) = \left( \frac{3}{2}q(\zeta ) \right) ^{\frac{2}{3}}, \end{aligned}$$

and *E* denotes the $2 \times 2$-matrix valued function analytic on ${\mathbb {D}}_\delta (b_{1})$ defined by

2.30 $$\begin{aligned} E(\zeta ) = P^{\infty }(\zeta ){\mathcal {G}}(\zeta )^{\frac{\sigma _{3}}{2}}\begin{pmatrix} 1 &{} i \\ 1 &{} -i \end{pmatrix}^{-1} \Big ( s^{\frac{2}{3}\rho }f(\zeta ) \Big )^{\frac{\sigma _{3}}{4}}. \end{aligned}$$

It is shown in [12, equation (3.71)] that, as $\zeta \rightarrow b_1$,

2.31 $$\begin{aligned} q(\zeta ) = -\frac{2}{3} \frac{(c_1+c_2)}{\sqrt{2}} \frac{\sqrt{|\mathrm{Re}\,b_1|}}{b_1} (\zeta -b_1)^{\frac{3}{2}} +{{{\mathcal {O}}}}\Big ( (\zeta -b_1)^{\frac{5}{2}}\Big ), \end{aligned}$$

where the branch cut for $(\zeta -b_1)^{\frac{3}{2}}$ runs along $\Sigma _5$ and $(\zeta -b_1)^{\frac{3}{2}} > 0$ for $\zeta -b_1 > 0$. Hence *f* is a conformal map from ${\mathbb {D}}_\delta (b_{1})$ to a neighborhood of 0 such that $\arg f'(b_1) = 2\phi /3 \in [0, \pi /3)$.

#### The solution *R*

In view of (2.3) and (2.17), the function ${\mathcal {G}}(\zeta )$ is not bounded as $s^{\rho }\zeta \rightarrow \infty$. Therefore, in the definition of the last transformation $S \mapsto R$, we need to multiply by a conjugation matrix $e^{p_{0}\sigma _{3}}$ in order for *R* to be uniformly bounded on ${\mathbb {C}}$.^5^ More precisely, we define *R* by

2.32 $$\begin{aligned} R(\zeta ) = e^{p_0 \sigma _3} S(\zeta ) \times {\left\{ \begin{array}{ll} P(\zeta )^{-1} e^{- p_0 \sigma _3}, &{}\text { if } \zeta \in {\mathbb {D}}_\delta (b_1)\cup {\mathbb {D}}_\delta (b_2), \\ P^{\infty }(\zeta )^{-1} e^{-p_0 \sigma _3}, &{}\text { elsewhere}. \end{array}\right. } \end{aligned}$$

Then $R(\zeta )$ is analytic for $\zeta \in {\mathbb {C}}{\setminus } \Gamma _{R}$ where $\Gamma _R$ consists of the parts of $\cup _{i=1}^5 \Sigma _i$ lying outside the disks ${\mathbb {D}}_\delta (b_j)$, $j = 1,2$, as well as the two clockwise circles $\partial {\mathbb {D}}_\delta (b_j)$, $j = 1,2$, see Fig. 4. We will show in Sect. 4 that *R* satisfies a small norm RH problem and that

2.33 $$\begin{aligned}&R(\zeta ) = I + \frac{R_{1}}{\zeta } + {{{\mathcal {O}}}}(\zeta ^{-2}) \qquad \text{ as } \zeta \rightarrow \infty , \end{aligned}$$

where the matrix $R_{1}$ possesses the asymptotics

2.34 $$\begin{aligned} R_{1} = \frac{R_{1}^{(1)}}{s^{\rho }} + {{{\mathcal {O}}}}(s^{-2\rho }) \qquad \text{ as } s \rightarrow + \infty , \end{aligned}$$

for a certain matrix $R_{1}^{(1)}$ independent of *s* and $\zeta$.

### Differential identity in *s*

It was proved in [12] that, for all $s > 0$,

2.35 $$\begin{aligned} \partial _{s} \ln \det \big ( 1-{\mathbb {K}}\big |_{[0,s]} \big )&\; = - \frac{1}{s}(Y_{1})_{2,2}\nonumber \\&\; = -\frac{(\mathrm{Re}\,b_2)^2 (c_1+c_2)}{8} s^{2\rho -1}-is^{\rho -1}\big ( -p_1(s)+(R_1(s))_{2,2} \big ). \end{aligned}$$

Furthermore, it was shown in [12, Section 4.3] that

2.36 $$\begin{aligned} \partial _{s} \ln \det \big ( 1-{\mathbb {K}}\big |_{[0,s]} \big ) =&- \frac{(\mathrm{Re}\,(b_{2}))^{2}(c_{1}+c_{2})}{8}s^{2 \rho -1}\nonumber \\&- \left( -c_{5} |b_{2}| + \frac{c_{5}+c_{6}}{2}(|b_{2}|-\mathrm{Im}\,(b_{2})) \right) s^{\rho -1} \nonumber \\&+ \frac{-{\mathcal {K}} + (R_{1}^{(1)})_{2,2}}{is} + {{{\mathcal {O}}}}(s^{-\rho -1}), \qquad \text{ as } s \rightarrow + \infty , \end{aligned}$$

where ${\mathcal {K}}$ is defined via the expansion (see [12, Eq. (4.15)])

2.37 $$\begin{aligned} p_1=-ic_{5}|b_{2}| + i \frac{c_{5}+c_{6}}{2}(|b_{2}|-\mathrm{Im}\,{b_2}) +\frac{{\mathcal {K}}}{s^\rho } + {{{\mathcal {O}}}}\bigg ( \frac{1}{s^{2\rho }} \bigg ), \qquad s \rightarrow + \infty . \end{aligned}$$

Integration of (2.36) yields the expressions in (1.9) for the first two coefficients *a* and *b*. Moreover, comparing (2.36) with (1.29) , we infer that the coefficient *c* can be expressed as

2.38 $$\begin{aligned} c = \frac{-{\mathcal {K}} + (R_{1}^{(1)})_{2,2}}{i}. \end{aligned}$$

Thus, to compute *c* it is enough to compute ${\mathcal {K}}$ and the (2, 2) entry of $R_{1}^{(1)}$.

## Asymptotics of ${\mathcal {G}} (\zeta )$ and $p(\zeta )$

In this section, we establish asymptotic formulas for the functions ${\mathcal {G}} (\zeta )$ and $p(\zeta )$ defined in (2.5) and (2.16) as $s \rightarrow + \infty$ with $\zeta$ such that $s^{\rho }\zeta \rightarrow \infty$. More precisely, we will prove that $\ln {\mathcal {G}} (\zeta )$ and $p(\zeta )$ admit expansions to all orders in inverse powers of $s^{\rho }\zeta$ and we will compute the coefficients of the expansion for $p(\zeta )$ explicitly up to and including the term of order $s^{-\rho }\zeta ^{-1}$ (this term plays a role in the derivation of the expressions for both *c* and *C*). The results are summarized in the followings two propositions whose proofs are presented in Sects. 3.1 and 3.2 , respectively. We let $\{c_j = c_j(\theta , \alpha )\}_1^8$ and $\{b_j = b_j(\theta , \alpha )\}_1^2$ be the constants defined in (2.4) and (2.7), respectively.

### Proposition 3.1

(Asymptotics of $\ln {\mathcal {G}} (\zeta )$). Let $N\ge 1$ be an integer. Let $\alpha > -1$ and $\theta \in (0, 1]$. There exist coefficients $\{{\mathcal {G}}_n = {\mathcal {G}}_n(\theta , \alpha )\}_1^N \subset {\mathbb {C}}$ such that the function ${\mathcal {G}}$ defined in (2.5) satisfies the asymptotic expansion

3.1 $$\begin{aligned} \ln {\mathcal {G}} (\zeta ) = c_4 \ln s + c_5\ln (i\zeta )+c_6\ln (-i\zeta )+c_7 + \frac{{\mathcal {G}}_{1}}{s^{\rho }\zeta } + \sum _{n=2}^{N} \frac{{\mathcal {G}}_n}{(s^\rho \zeta )^n} + {{{\mathcal {O}}}}\bigg ( \frac{1}{(s^\rho \zeta )^{N+1}}\bigg ) \end{aligned}$$

as $s^\rho \zeta \rightarrow \infty$ uniformly for $\theta$ in compact subsets of (0, 1] and $\zeta \in {\mathbb {C}}$ such that $|\arg (\zeta ) - \tfrac{\pi }{2}| > \epsilon$ and $|\arg (\zeta ) + \tfrac{\pi }{2}| > \epsilon$ for any fixed $\epsilon > 0$. The first coefficient is given by ${\mathcal {G}}_1= -ic_8$.

### Proposition 3.2

(Asymptotics of $p(\zeta )$). Let $N\ge 1$ be an integer. Let $\alpha > -1$ and $\theta \in (0, 1]$. There exist holomorphic functions ${\mathcal {A}}_n:{\mathbb {C}}{\setminus } \Sigma _5 \rightarrow {\mathbb {C}}$, $n=1,\ldots N$, with ${\mathcal {A}}_n(\zeta ) = {{{\mathcal {O}}}}(\zeta ^n)$ as $\zeta \rightarrow \infty$, such that

3.2 $$\begin{aligned} p(\zeta )=&-\frac{c_4}{2} \ln (s) -\frac{c_5}{2} \ln (i\zeta )-\frac{c_6}{2}\ln (-i\zeta )-\frac{c_7}{2} + \frac{{\mathcal {R}}(\zeta )}{2} \nonumber \\&+ \frac{{\mathcal {A}}_{1}(\zeta )}{s^{\rho } \zeta } + \sum _{n=2}^{N} \frac{{\mathcal {A}}_n(\zeta )}{(s^\rho \zeta )^n} + {{{\mathcal {O}}}}\bigg ( \frac{1}{(s^\rho \zeta )^{N+1}}\bigg ) + {{{\mathcal {O}}}}\bigg ( \frac{1}{s^{(N+1)\rho }}\bigg ) \qquad \text{ as } s \rightarrow +\infty , \end{aligned}$$

uniformly with respect to $\zeta \in {\mathbb {C}}{\setminus } \Sigma _5$ such that $s^{\rho }\zeta \rightarrow \infty$ and $\theta$ in compact subsets of (0, 1], where the functions ${\mathcal {R}}(\zeta )$ and ${\mathcal {A}}_1(\zeta )$ are given by

3.3 $$\begin{aligned} {\mathcal {R}}(\zeta )&= -c_5 \ln \bigg (\frac{|b_2|^2 + i\zeta \mathrm{Im}\,{b_2} - i|b_2| r(\zeta )}{(r(\zeta ) + \zeta - i\mathrm{Im}\,{b_2})\zeta }\bigg )-c_6\ln \bigg (\frac{|b_2|^2 + i\zeta \mathrm{Im}\,{b_2} +i|b_2| r(\zeta )}{(r(\zeta ) + \zeta - i\mathrm{Im}\,{b_2})\zeta }\bigg ) \end{aligned}$$

and

3.4 $$\begin{aligned} {\mathcal {A}}_1(\zeta ) = \frac{ic_8}{2}+\frac{c_8-\frac{3\alpha ^2-1}{12}}{2|b_2|}r(\zeta ). \end{aligned}$$

### Remark 3.3

The expansion in (3.2) is well-defined also for $\zeta \in i{\mathbb {R}}{\setminus } \{ 0 \}$ even though several of the coefficients have jumps across the imaginary axis. Indeed, it can be seen from (3.3) (and more easily from the integral representation (3.38) of ${\mathcal {R}}$) that ${\mathcal {R}}$ has the following jump across the imaginary axis:

$$\begin{aligned} {\mathcal {R}}_+(\zeta ) - {\mathcal {R}}_-(\zeta ) = {\left\{ \begin{array}{ll} 2\pi i c_5, &{} \zeta \in (i\infty , 0), \\ 2\pi i c_6, &{} \zeta \in (-i\infty , 0), \end{array}\right. } \end{aligned}$$

where $(i\infty , 0)$ and $(-i\infty , 0)$ are oriented towards the origin. It follows that the function

$$\begin{aligned} -\frac{c_5}{2} \ln (i\zeta )-\frac{c_6}{2}\ln (-i\zeta )-\frac{c_7}{2} + \frac{{\mathcal {R}}(\zeta )}{2} \end{aligned}$$

has no jump across the imaginary axis and hence extends to an analytic function on ${\mathbb {C}}{\setminus } \Sigma _5$.

### Remark 3.4

The expansion of $\ln {\mathcal {G}}(\zeta )$ as $s^{\rho } \zeta \rightarrow \infty$ up to and including the term of order $s^{-\rho }\zeta ^{-1}$ is easily obtained from (2.3) and (2.17), see [12, Eq. (3.15)]. The extension of this expansion to all orders is not straightforward and is the content of Proposition 3.1.

### Remark 3.5

The assumption that $0 < \theta \le 1$ implies that $\phi = \arg b_{2}$ satisfies $0\le \phi <\frac{\pi }{2}$, see Fig. 2.

### Proof of Proposition 3.1

We will employ the following exact representation for $\ln \Gamma (z)$ (see [30, Eq. (6.34) with $r=N$ and Eq. (6.38)]):

3.5 $$\begin{aligned} \ln \Gamma (z)&= z\ln z-z-\frac{1}{2} \ln (z) + \ln \sqrt{2\pi } +\frac{1}{12z} +\sum _{n=2}^{N}\frac{B_{2n}}{(2n-1)2n} \frac{1}{z^{2n-1}} + {\mathcal {D}}_{N}(z), \end{aligned}$$

3.6 $$\begin{aligned} {\mathcal {D}}_{N}(z)&= -\frac{1}{2N+1}\int _{0}^{\infty } \frac{B_{2N+1}(\{t\})}{(z+t)^{2N+1}} dt, \end{aligned}$$

which is valid for $|\arg z|<\pi$, with *N* an arbitrary (but fixed) positive integer and where $\{ t \}$ denotes the fractional part of *t*, i.e., $\{t\} = t - \lfloor t \rfloor$ where $\lfloor t \rfloor$ is the largest integer smaller than or equal to *t*. Here $B_{n}$ is the *n*th Bernoulli number and $B_{N}(x)$ the *N*th Bernoulli polynomial given by (see e.g. [1, p. 804])

$$\begin{aligned} B_{N}(x) = \sum _{n=0}^{N} \left( {\begin{array}{c}N\\ n\end{array}}\right) B_{N-n}x^n. \end{aligned}$$

The first terms on the right-hand side of (3.5) are the same as in Stirling’s approximation formula; however (3.5) is an exact identity which is valid for all $z \in {\mathbb {C}}$ such that $|\arg z| < \pi$. It is straightforward to verify that (see [30, last equation on page 78] for details)

3.7 $$\begin{aligned} {\mathcal {D}}_N(z)= {{{\mathcal {O}}}}(z^{-2N-1}), \qquad z \rightarrow \infty , \; |\arg z| < \pi - \epsilon , \end{aligned}$$

for any fixed $\epsilon > 0$. Using the short-hand notation

3.8 $$\begin{aligned} x(\xi )=x(\xi ,s,\theta , \alpha )=is^\rho \xi + \frac{1+\alpha }{2}, \qquad y(\xi )=y(\xi ,s,\theta ,\alpha )=\frac{\frac{1+\alpha }{2}-is^\rho \xi }{\theta },\qquad \end{aligned}$$

we have

3.9 $$\begin{aligned}&x(\xi ) \le 0 \qquad \text{ if } \text{ and } \text{ only } \text{ if } \qquad \xi \in \big [ \tfrac{1+\alpha }{2}is^{-\rho },i\infty \big ), \end{aligned}$$

3.10 $$\begin{aligned}&y(\xi ) \le 0 \qquad \text{ if } \text{ and } \text{ only } \text{ if } \qquad \xi \in \big [ -\tfrac{1+\alpha }{2}is^{-\rho },-i\infty \big ). \end{aligned}$$

Therefore, for all

$$\begin{aligned} \xi \in {\mathbb {C}}{\setminus } \big ( \big [ \tfrac{1+\alpha }{2}is^{-\rho },i\infty \big ) \cup \big [ -\tfrac{1+\alpha }{2}is^{-\rho },-i\infty \big )\big ), \end{aligned}$$

we can use (3.5) together with (2.1) to write

$$\begin{aligned}&\ln F(is^\rho \xi +1/2)= \ln \Gamma (x(\xi )) - \ln \Gamma (y(\xi )) \\&\quad = \; x(\xi )\ln (x(\xi ))-x(\xi )-\frac{1}{2}\ln (x(\xi ))+\ln \sqrt{2\pi }+\frac{1}{12x(\xi )} \\&\qquad +\sum _{n=2}^{N}\frac{B_{2n}}{(2n-1)2n} \frac{1}{x(\xi )^{2n-1}}\\&\qquad +{\mathcal {D}}_N(x(\xi )) \\&\qquad -y(\xi )\ln (y(\xi ))+y(\xi )+\frac{1}{2}\ln (y(\xi ))-\ln \sqrt{2\pi }-\frac{1}{12y(\xi )}\\&\qquad -\sum _{n=2}^{N}\frac{B_{2n}}{(2n-1)2n} \frac{1}{y(\xi )^{2n-1}}\\&\qquad -{\mathcal {D}}_N(y(\xi )). \end{aligned}$$

Hence, by (2.17) and (2.6) we have, for any fixed $N\ge 1$,

3.11 $$\begin{aligned} \ln {\mathcal {G}}(\xi )= {\hat{f}}(\xi )+{\tilde{f}}(\xi ) +{\mathcal {D}}_N(x(\xi ))-{\mathcal {D}}_N(y(\xi )), \end{aligned}$$

where the functions ${\hat{f}}(\xi )$ and ${\tilde{f}}(\xi )$ are defined by

3.12a $$\begin{aligned} {\hat{f}}(\xi )&= x(\xi )\ln (x(\xi ))-x(\xi )-\frac{1}{2}\ln (x(\xi ))+\frac{1}{12x(\xi )}-is^{\rho }\xi \big (a_1\ln (s)+c_1\ln (i\xi )+a_2\big ) \nonumber \\&\quad +\sum _{n=2}^{N}\frac{B_{2n}}{(2n-1)2n} \frac{1}{x(\xi )^{2n-1}}, \\ {\tilde{f}}(\xi )&= -\,y(\xi )\ln (y(\xi ))+y(\xi )+\frac{1}{2}\ln (y(\xi ))-\frac{1}{12y(\xi )}\nonumber \\&\quad -\,is^{\rho }\xi \big ({\tilde{a}}_1\ln (s)+c_2\ln (-i\xi )+{\tilde{a}}_2\big ) \nonumber \\&\quad -\,\sum _{n=2}^{N}\frac{B_{2n}}{(2n-1)2n} \frac{1}{y(\xi )^{2n-1}} , \end{aligned}$$

with the real constants $a_1, a_2, {\tilde{a}}_1, {\tilde{a}}_2$ defined by

$$\begin{aligned} a_1=\frac{\theta }{\theta +1}, \quad a_2=-1, \quad {\tilde{a}}_1=1-a_1=\frac{1}{\theta +1}, \quad {\tilde{a}}_2=c_3-a_2=-\frac{1+\ln \theta }{\theta }. \end{aligned}$$

The functions ${\hat{f}}(\xi )$, ${\mathcal {D}}_N(x(\xi ))$ and ${\tilde{f}}(\xi )$, ${\mathcal {D}}_N(y(\xi ))$ are analytic for

3.12b $$\begin{aligned} \xi \in {\mathbb {C}}{\setminus } \big [ \tfrac{1+\alpha }{2}is^{-\rho },i\infty \big ) \quad \text { and } \quad \xi \in {\mathbb {C}}{\setminus } \big [- \tfrac{1+\alpha }{2}is^{-\rho },-i\infty \big ), \end{aligned}$$

respectively. The asymptotics of ${\hat{f}}$ and ${\tilde{f}}$ as $s^{\rho }\xi \rightarrow \infty$ are easily obtained from (3.12):

3.13 $$\begin{aligned} {\hat{f}}(\xi )&= a_3\ln (s)+c_5\ln (i\xi )+a_4+\sum _{n=1}^{N} \frac{{\hat{f}}_n}{(s^{\rho }\xi )^{n}}+{{{\mathcal {O}}}}\bigg ( \frac{1}{(s^\rho \xi )^{N+1}} \bigg ), \end{aligned}$$

3.14a $$\begin{aligned} {\tilde{f}}(\xi )&={\tilde{a}}_3\ln (s)+c_6\ln (-i\xi )+{\tilde{a}}_4+\sum _{n=1}^{N} \frac{{\tilde{f}}_n}{(s^{\rho }\xi )^{n}}+{{{\mathcal {O}}}}\bigg ( \frac{1}{(s^\rho \xi )^{N+1}}\bigg ), \end{aligned}$$

as $s^\rho \xi \rightarrow \infty$ uniformly for $\theta$ in compact subsets of (0, 1], where the constants $\{a_j, {\tilde{a}}_j\}_3^4 \subset {\mathbb {R}}$ and ${\hat{f}}_1, {\tilde{f}}_1 \in i{\mathbb {R}}$ are defined by

3.14b $$\begin{aligned} a_3&= \frac{\alpha \theta }{2(\theta +1)},&a_4&=0,&{\hat{f}}_1= -i\frac{3\alpha ^2-1}{24}, \end{aligned}$$

3.15 $$\begin{aligned} {\tilde{a}}_3&=c_4-a_3,&{\tilde{a}}_4&=c_7,&{\tilde{f}}_1= -ic_8-{\hat{f}}_1, \end{aligned}$$

and $\{{\hat{f}}_n,{\tilde{f}}_n\}_{n=2}^N \subset {\mathbb {C}}$ are constants whose exact expressions are unimportant for us. However, we note that ${\hat{f}}_n(\theta ,\alpha )$ and ${\tilde{f}}_n(\theta ,\alpha )$ are continuous functions of $\alpha$ and $\theta$. From (3.7) and (3.8), we infer that

3.16 $$\begin{aligned}&{\mathcal {D}}_{N}(x(\xi )) = {{{\mathcal {O}}}}\big ( (s^\rho \xi )^{-2N-1}\big )&\text{ as } s^{\rho }\xi \rightarrow \infty , \quad |\arg (\xi ) - \tfrac{\pi }{2}| > \epsilon , \end{aligned}$$

3.17 $$\begin{aligned}&{\mathcal {D}}_{N}(y(\xi )) = {{{\mathcal {O}}}}\big ( (s^\rho \xi )^{-2N-1}\big )&\text{ as } s^{\rho }\xi \rightarrow \infty , \quad |\arg (\xi ) + \tfrac{\pi }{2}| > \epsilon , \end{aligned}$$

for any $\epsilon > 0$ uniformly for $\theta$ in compact subsets of (0, 1]. Substituting (3.14)–(3.18) into (3.11), we obtain (3.1) where the coefficients ${\mathcal {G}}_{n}$ are given by ${\mathcal {G}}_n ={\hat{f}}_n+{\tilde{f}}_n$; in particular, ${\mathcal {G}}_1= -ic_8$. This completes the proof of Proposition 3.1.

### Proof of Proposition 3.2

Recall that $p(\zeta )$ is defined by

3.18 $$\begin{aligned} p(\zeta )= - \frac{r(\zeta )}{2\pi i} \int _{\Sigma _{5}} \frac{\ln {\mathcal {G}}(\xi )}{r_{+}(\xi )}\frac{d\xi }{\xi -\zeta }. \end{aligned}$$

Since $\Sigma _{5}$ passes through the origin, the large *s* asymptotics for *p* cannot be straightforwardly obtained from the asymptotics (3.1) of $\ln {\mathcal {G}}(\zeta )$. We instead use formula (3.11) to be able to deform the contour $\Sigma _5$. Substituting (3.11) into the definition (3.19) of $p(\zeta )$ yields

3.19 $$\begin{aligned} p(\zeta )=&- \frac{r(\zeta )}{2\pi i} \int _{\Sigma _{5}} \frac{{\hat{f}}(\xi )}{r_{+}(\xi )}\frac{d\xi }{\xi -\zeta }- \frac{r(\zeta )}{2\pi i} \int _{\Sigma _{5}} \frac{{\tilde{f}}(\xi )}{r_{+}(\xi )}\frac{d\xi }{\xi -\zeta } \nonumber \\&-\frac{r(\zeta )}{2\pi i} \int _{\Sigma _{5}} \frac{{\mathcal {D}}_N(x(\xi ))}{r_{+}(\xi )(\xi -\zeta )} d\xi +\frac{r(\zeta )}{2\pi i} \int _{\Sigma _{5}} \frac{{\mathcal {D}}_N(y(\xi ))}{r_{+}(\xi )(\xi -\zeta )} d\xi . \end{aligned}$$

The remainder of the proof is divided into three lemmas. The first lemma shows that the two integrals in (3.20) involving ${\mathcal {D}}_N$ are small whenever $s^\rho$ and $s^{\rho }\zeta$ are large.

#### Lemma 3.6

For any integer $N\ge 1$, it holds that

3.20 $$\begin{aligned}&\frac{r(\zeta )}{2\pi i} \int _{\Sigma _{5}} \frac{{\mathcal {D}}_N(x(\xi ))}{r_{+}(\xi )(\xi -\zeta )} d\xi = {{{\mathcal {O}}}}\big ((s^{\rho }\zeta )^{-2N-1}\big ) + {{{\mathcal {O}}}}\big (s^{-\rho (2N+1)}\big ) \qquad \text{ as } s \rightarrow +\infty , \end{aligned}$$

3.21a $$\begin{aligned}&\frac{r(\zeta )}{2\pi i} \int _{\Sigma _{5}} \frac{{\mathcal {D}}_N(y(\xi ))}{r_{+}(\xi )(\xi -\zeta )} d\xi = {{{\mathcal {O}}}}\big ((s^{\rho }\zeta )^{-2N-1}\big ) + {{{\mathcal {O}}}}\big (s^{-\rho (2N+1)}\big ) \qquad \text{ as } s \rightarrow +\infty , \end{aligned}$$

uniformly for $\zeta \in {\mathbb {C}}{\setminus } \Sigma _5$ such that $s^{\rho }\zeta \rightarrow \infty$ and $\theta$ in compact subsets of (0, 1].

#### Proof

Given $\zeta \in {\mathbb {C}}{\setminus } \Sigma _5$, the integrand in (3.21a) is an analytic function of

$$\begin{aligned} \xi \in {\mathbb {C}}{\setminus } \Big (\Sigma _5 \cup \big [ \tfrac{1+\alpha }{2}is^{-\rho },i\infty \big )\cup \{\zeta \}\Big ), \end{aligned}$$

see (3.13). Using that $r_{+}(\xi ) + r_{-}(\xi ) = 0$ for $\xi \in \Sigma _{5}$, we can deform the contour $\Sigma _{5}$ into another contour ${\hat{\sigma }}$ which crosses the imaginary axis below the origin such that

**Fig. 5:**
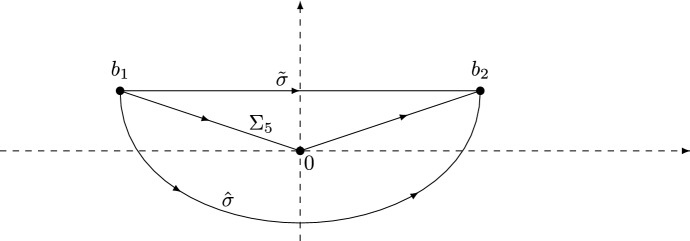
The contours ${\hat{\sigma }}$ and ${\tilde{\sigma }}$ for $\zeta \notin {\mathbb {D}}_{\delta /2}(b_1)\cup {\mathbb {D}}_{\delta /2}(b_2)$

3.21b $$\begin{aligned}&|\xi |> \epsilon \text{ and } |\arg (\xi ) - \tfrac{\pi }{2}|> \epsilon \quad \text{ for } \text{ all } \xi \in {\hat{\sigma }}, \quad \text{ for } \text{ a } \text{ certain } \epsilon > 0, \end{aligned}$$

and such that ${{\,\mathrm{dist}\,}}(\zeta , {\hat{\sigma }}) \ge \delta /2$. If $\zeta \notin {\mathbb {D}}_{\delta /2}(b_1)\cup {\mathbb {D}}_{\delta /2}(b_2)$, a representative choice of ${\hat{\sigma }}$ is shown in Fig. 5, and we obtain

**Fig. 6:**
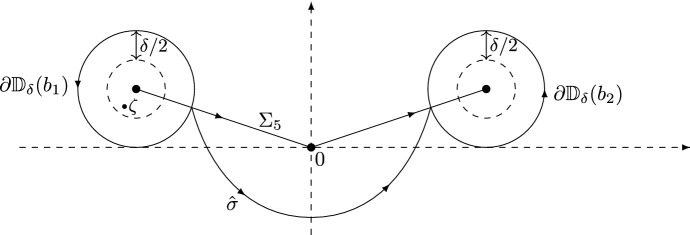
The contour ${\hat{\sigma }}$ for $\zeta \in {\mathbb {D}}_{\delta /2}(b_1)\cup {\mathbb {D}}_{\delta /2}(b_2)$

3.22 $$\begin{aligned}&\frac{r(\zeta )}{2\pi i} \int _{\Sigma _{5}} \frac{{\mathcal {D}}_N(x(\xi ))}{r_{+}(\xi )(\xi -\zeta )} d\xi \nonumber \\&\quad = \left\{ \begin{array}{l l} \displaystyle -\frac{r(\zeta )}{2\pi i} \int _{{\hat{\sigma }}} \frac{{\mathcal {D}}_N(x(\xi ))}{r(\xi )(\xi -\zeta )} d\xi , &{} \zeta \in \text{ ext }({\hat{\sigma }} \cup \Sigma _{5}), \\ \displaystyle -\frac{r(\zeta )}{2\pi i} \int _{{\hat{\sigma }}} \frac{{\mathcal {D}}_N(x(\xi ))}{r(\xi )(\xi -\zeta )} d\xi + {\mathcal {D}}_{N}(x(\zeta )), &{} \zeta \in \text{ int }({\hat{\sigma }} \cup \Sigma _{5}), \end{array} \right. \end{aligned}$$

where, for a simple closed curve $\gamma \subset {\mathbb {C}}$, we write $\text{ int }(\gamma )$ and $\text{ ext }(\gamma )$ for the open subsets of ${\mathbb {C}}$ interior and exterior to $\gamma$, respectively. If $\zeta \in {\mathbb {D}}_{\delta /2}(b_1)\cup {\mathbb {D}}_{\delta /2}(b_2)$, then we use the jump relation of $r(\zeta )$ to open up the parts of ${\hat{\sigma }}$ close to the points $b_1$ and $b_2$ to two circles in such a way that $\partial {\mathbb {D}}_{\delta }(b_1)\cup \partial {\mathbb {D}}_{\delta }(b_2) \subset {\hat{\sigma }}$, see Fig. 6, and instead of (3.23) we obtain

$$\begin{aligned}&\frac{r(\zeta )}{2\pi i} \int _{\Sigma _{5}} \frac{{\mathcal {D}}_N(x(\xi ))}{r_{+}(\xi )(\xi -\zeta )} d\xi \nonumber \\&\quad =-\frac{r(\zeta )}{4\pi i} \int _{\partial {\mathbb {D}}_{\delta }} \frac{{\mathcal {D}}_N(x(\xi ))}{r(\xi )(\xi -\zeta )} d\xi -\frac{r(\zeta )}{2\pi i} \int _{{\hat{\sigma }}{\setminus } \partial {\mathbb {D}}_{\delta }} \frac{{\mathcal {D}}_N(x(\xi ))}{r(\xi )(\xi -\zeta )} d\xi \\&\quad + \frac{{\mathcal {D}}_{N}(x(\zeta ))}{2}, \end{aligned}$$

where $\partial {\mathbb {D}}_{\delta }:=\partial {\mathbb {D}}_{\delta }(b_1)\cup \partial {\mathbb {D}}_{\delta }(b_2)$. The cases $\zeta \notin {\mathbb {D}}_{\delta /2}(b_1)\cup {\mathbb {D}}_{\delta /2}(b_2)$ and $\zeta \in {\mathbb {D}}_{\delta /2}(b_1)\cup {\mathbb {D}}_{\delta /2}(b_2)$ can be treated similarly. Since (3.22) holds, we can apply (3.17), which implies the estimate ${\mathcal {D}}_N(x(\xi )) = {{{\mathcal {O}}}}(s^{-\rho (2N+1)})$ as $s \rightarrow +\infty$ uniformly for $\xi \in {\hat{\sigma }}$. Since $r(\zeta ) \sim \zeta$ as $\zeta \rightarrow \infty$ and $\text {dist}(\zeta ,{\hat{\sigma }}) \ge \delta /2$, we find

$$\begin{aligned} {\left\{ \begin{array}{ll} \displaystyle -\frac{r(\zeta )}{2\pi i} \int _{{\hat{\sigma }}} \frac{{\mathcal {D}}_N(x(\xi ))}{r(\xi )(\xi -\zeta )} d\xi = {{{\mathcal {O}}}}(s^{-\rho (2N+1)}), &{} \displaystyle \text{ if } \zeta \notin {\mathbb {D}}_{\delta /2}(b_1)\cup {\mathbb {D}}_{\delta /2}(b_2) \\ \displaystyle -\frac{r(\zeta )}{4\pi i} \int _{\partial {\mathbb {D}}_{\delta }} \frac{{\mathcal {D}}_N(x(\xi ))}{r(\xi )(\xi -\zeta )} d\xi -\frac{r(\zeta )}{2\pi i} \int _{{\hat{\sigma }}{\setminus } \partial {\mathbb {D}}_{\delta }} \frac{{\mathcal {D}}_N(x(\xi ))}{r(\xi )(\xi -\zeta )} d\xi = {{{\mathcal {O}}}}(s^{-\rho (2N+1)}) &{} \displaystyle \text{ if } \zeta \in {\mathbb {D}}_{\delta /2}(b_1)\cup {\mathbb {D}}_{\delta /2}(b_2) \end{array}\right. } \end{aligned}$$

as $s\rightarrow +\infty$ uniformly for $\zeta \in {\mathbb {C}}{\setminus } \Sigma _{5}$ and $\theta$ in compact subsets of (0, 1]. The term ${\mathcal {D}}_N(x(\zeta ))$ is present in the case $\zeta \in {\mathbb {D}}_{\delta /2}(b_1)\cup {\mathbb {D}}_{\delta /2}(b_2)$, and also in (3.23) if $\zeta \in \text{ int }({\hat{\sigma }} \cup \Sigma _{5})$. Since $|\arg (\zeta )- \tfrac{\pi }{2}|>\epsilon$ for a certain $\epsilon > 0$, and since $s^{\rho }\zeta \rightarrow \infty$ by assumption, we can apply (3.17) to obtain

$$\begin{aligned} {\mathcal {D}}_N(x(\zeta )) = {{{\mathcal {O}}}}((s^{\rho }\zeta )^{-2N-1}). \end{aligned}$$

This proves (3.21a). A similar argument based on deforming $\Sigma _{5}$ into a contour ${\tilde{\sigma }}$ which crosses the imaginary axis above the origin (see Fig. 5 in the case when $\zeta \notin {\mathbb {D}}_{\delta /2}(b_1)\cup {\mathbb {D}}_{\delta /2}(b_2)$) yields (3.21b). $\quad \square$

It remains to compute the asymptotics of the two integrals in (3.20) involving ${\hat{f}}$ and ${\tilde{f}}$. Since $\Sigma _{5}$ passes through the origin, we cannot immediately use the asymptotic formulas (3.14) for ${\hat{f}}$ and ${\tilde{f}}$. However, since ${\hat{f}}$ and ${\tilde{f}}$ are analytic in the regions (3.13), we can deform the contours in the same way as in the proof of Lemma 3.6 and then use (3.14).

#### Definition 3.7

Let $\epsilon > 0$ be sufficiently small but fixed. We define contours ${\widehat{\Sigma }}_{5,\pm } = {\widehat{\Sigma }}_{5,\pm }(\epsilon )$ as follows:

$$\begin{aligned}&{\widehat{\Sigma }}_{5,+}(\epsilon ) = \big ( \Sigma _{5} \cap \{|\xi |> \epsilon \} \big ) \cup \{ \xi : |\xi | = \epsilon \text{ and } \phi< \arg \xi< \pi - \phi \}, \\&{\widehat{\Sigma }}_{5,-}(\epsilon ) = \big ( \Sigma _{5} \cap \{|\xi | > \epsilon \} \big ) \cup \{ \xi : |\xi | = \epsilon \text{ and } -\pi -\phi< \arg \xi < \phi \}, \end{aligned}$$

with an orientation chosen from $b_{1}$ to $b_{2}$. Thus ${\widehat{\Sigma }}_{5,\pm }$ coincide with $\Sigma _{5}$ outside the disks $\{|\xi | \le \epsilon \}$, and inside this disk, they differ from $\Sigma _{5}$ and instead coincide with the part of the circle $\{|\xi | = \epsilon \}$ lying above (resp. below) $\Sigma _{5}$.

#### Lemma 3.8

For each integer $N\ge 1$, it holds that

3.23 $$\begin{aligned} p(\zeta ) =&-(c_{4} \ln (s) + c_{7}) \frac{r(\zeta )}{2\pi i} \int _{\Sigma _{5}} \frac{1}{r_{+}(\xi )}\frac{d\xi }{\xi -\zeta } \nonumber \\&-r(\zeta ) \bigg \{\frac{c_{5}}{2\pi i} \int _{\Sigma _{5}} \frac{\ln (i\xi )}{r_{+}(\xi )}\frac{d\xi }{\xi -\zeta } + \frac{c_6}{2\pi i} \int _{\Sigma _{5}} \frac{\ln (-i\xi )}{r_{+}(\xi )}\frac{d\xi }{\xi -\zeta } \bigg \} \nonumber \\&+\sum _{n=1}^{N} \frac{1}{s^{n\rho }} \bigg \{{\hat{f}}_n \frac{r(\zeta )}{2\pi i} \int _{\Sigma _{5,-}} \frac{ 1}{\xi ^{n}r_{-}(\xi )}\frac{d\xi }{\xi -\zeta } - {\tilde{f}}_n \frac{r(\zeta )}{2\pi i} \int _{\Sigma _{5,+}} \frac{ 1}{\xi ^{n}r_{+}(\xi )}\frac{d\xi }{\xi -\zeta } \bigg \} \nonumber \\&+ {{{\mathcal {O}}}}\big (( s^{\rho }\zeta )^{-N-1}\big ) + {{{\mathcal {O}}}}\big (s^{-\rho (N+1)}\big ), \qquad s \rightarrow +\infty , \end{aligned}$$

uniformly for $\zeta \in {\mathbb {C}}{\setminus } \Sigma _5$ such that $s^{\rho }\zeta \rightarrow \infty$ and $\theta$ in compact subsets of (0, 1], and where the contours $\Sigma _{5,\pm }$ depend on $\epsilon$ and $\zeta$ and are given by

$$\begin{aligned} \Sigma _{5,\pm }:= {\widehat{\Sigma }}_{5,\pm }(\min \{\epsilon , \tfrac{\zeta }{2}\}) \end{aligned}$$

with ${\widehat{\Sigma }}_{5,\pm }(\min \{\epsilon , \frac{\zeta }{2}\})$ as in Definition 3.7.

#### Proof

Let us assume that $\zeta \notin {\mathbb {D}}_{\delta /2}(b_1)\cup {\mathbb {D}}_{\delta /2}(b_2)$. For the integral involving ${\hat{f}}$ (resp. ${\tilde{f}}$), we deform $\Sigma _{5}$ into ${\hat{\sigma }}$ (resp. ${\tilde{\sigma }}$), and we pick up a residue if $\zeta \in \text{ int }( {\hat{\sigma }} \cup \Sigma _{5})$ (resp. if $\zeta \in \text{ int }( {\tilde{\sigma }} \cup \Sigma _{5})$). This gives

3.24 $$\begin{aligned} - \frac{r(\zeta )}{2\pi i} \int _{\Sigma _{5}} \frac{{\hat{f}}(\xi )}{r_{+}(\xi )}\frac{d\xi }{\xi -\zeta }&= \frac{r(\zeta )}{2\pi i} \int _{{\hat{\sigma }}} \frac{{\hat{f}}(\xi )}{r(\xi )}\frac{d\xi }{\xi -\zeta } - \chi _{\text{ int }({\hat{\sigma }} \cup \Sigma _5)}(\zeta ) {\hat{f}}(\zeta ), \end{aligned}$$

3.25a $$\begin{aligned} - \frac{r(\zeta )}{2\pi i} \int _{\Sigma _{5}} \frac{{\tilde{f}}(\xi )}{r_{+}(\xi )}\frac{d\xi }{\xi -\zeta }&= - \frac{r(\zeta )}{2\pi i} \int _{{\tilde{\sigma }}} \frac{{\tilde{f}}(\xi )}{r(\xi )}\frac{d\xi }{\xi -\zeta } - \chi _{\text{ int }({\tilde{\sigma }} \cup \Sigma _5)}(\zeta ) {\tilde{f}}(\zeta ). \end{aligned}$$

Since $|\xi | > \epsilon$ for $\xi \in {\hat{\sigma }} \cup {\tilde{\sigma }}$, the expansions (3.14) imply that the integrals on the right-hand side of (3.25) satisfy

3.25b $$\begin{aligned} \frac{r(\zeta )}{2\pi i} \int _{{\hat{\sigma }}} \frac{{\hat{f}}(\xi )}{r(\xi )}\frac{d\xi }{\xi -\zeta } =&\; (a_{3} \ln (s)+a_{4}) \frac{r(\zeta )}{2\pi i} \int _{{\hat{\sigma }}} \frac{1}{r(\xi )}\frac{d\xi }{\xi -\zeta }+ c_{5}\frac{r(\zeta )}{2\pi i} \int _{{\hat{\sigma }}} \frac{\ln (i\xi )}{r(\xi )}\frac{d\xi }{\xi -\zeta }\nonumber \\&\; +\sum _{n=1}^{N} \frac{{\hat{f}}_n}{s^{n\rho }} \frac{r(\zeta )}{2\pi i} \int _{{\hat{\sigma }}} \frac{ 1}{\xi ^{n}r(\xi )}\frac{d\xi }{\xi -\zeta } + {{{\mathcal {O}}}}(s^{-\rho (N+1)}), \end{aligned}$$

3.26a $$\begin{aligned} - \frac{r(\zeta )}{2\pi i} \int _{{\tilde{\sigma }}} \frac{{\tilde{f}}(\xi )}{r(\xi )}\frac{d\xi }{\xi -\zeta } =&-({\tilde{a}}_{3} \ln (s)+{\tilde{a}}_{4}) \frac{r(\zeta )}{2\pi i} \int _{{\tilde{\sigma }}} \frac{1}{r(\xi )}\frac{d\xi }{\xi -\zeta }- c_{6}\frac{r(\zeta )}{2\pi i} \int _{{\tilde{\sigma }}} \frac{\ln (-i\xi )}{r(\xi )}\frac{d\xi }{\xi -\zeta } \nonumber \\&-\sum _{n=1}^{N} \frac{{\tilde{f}}_n}{s^{n\rho }} \frac{r(\zeta )}{2\pi i} \int _{{\tilde{\sigma }}} \frac{ 1}{\xi ^{n}r(\xi )}\frac{d\xi }{\xi -\zeta } + {{{\mathcal {O}}}}(s^{-\rho (N+1)}), \end{aligned}$$

as $s \rightarrow + \infty$ uniformly for $\zeta \in {\mathbb {C}}{\setminus } \Sigma _5$ and $\theta$ in compact subsets of (0, 1]. Substituting (3.25) into (3.20) and utilizing (3.26) and Lemma 3.6 in the resulting expression for $p(\zeta )$, we conclude that

3.26b $$\begin{aligned} p(\zeta )=&\; {\mathfrak {p}}_1(\zeta ) +{\mathfrak {p}}_2(\zeta ) + {\mathfrak {p}}_3(\zeta ) - \chi _{\text{ int }({\hat{\sigma }} \cup \Sigma _5)}(\zeta ) {\hat{f}}(\zeta ) - \chi _{\text{ int }({\tilde{\sigma }} \cup \Sigma _5)}(\zeta ) {\tilde{f}}(\zeta )\nonumber \\&+ {{{\mathcal {O}}}}\big ((s^{\rho }\zeta )^{-2N-1}\big ) + {{{\mathcal {O}}}}\big (s^{-\rho (N+1)}\big ). \end{aligned}$$

where the functions $\{{\mathfrak {p}}_j(\zeta )\}_1^3$ are given by

3.27 $$\begin{aligned} {\mathfrak {p}}_1(\zeta ) =&\; (a_{3} \ln (s)+a_{4}) \frac{r(\zeta )}{2\pi i} \int _{{\hat{\sigma }}} \frac{1}{r(\xi )}\frac{d\xi }{\xi -\zeta } -({\tilde{a}}_{3} \ln (s)+{\tilde{a}}_{4}) \frac{r(\zeta )}{2\pi i} \int _{{\tilde{\sigma }}} \frac{1}{r(\xi )}\frac{d\xi }{\xi -\zeta }, \end{aligned}$$

3.28 $$\begin{aligned} {\mathfrak {p}}_2(\zeta ) =&\; c_{5}\frac{r(\zeta )}{2\pi i} \int _{{\hat{\sigma }}} \frac{\ln (i\xi )}{r(\xi )}\frac{d\xi }{\xi -\zeta } - c_{6}\frac{r(\zeta )}{2\pi i} \int _{{\tilde{\sigma }}} \frac{\ln (-i\xi )}{r(\xi )}\frac{d\xi }{\xi -\zeta }, \end{aligned}$$

3.29 $$\begin{aligned} {\mathfrak {p}}_3(\zeta ) =&\; \sum _{n=1}^{N} \frac{{\hat{f}}_n}{s^{n\rho }} \frac{r(\zeta )}{2\pi i} \int _{{\hat{\sigma }}} \frac{ 1}{\xi ^{n}r(\xi )}\frac{d\xi }{\xi -\zeta } -\sum _{n=1}^{N} \frac{{\tilde{f}}_n}{s^{n\rho }} \frac{r(\zeta )}{2\pi i} \int _{{\tilde{\sigma }}} \frac{ 1}{\xi ^{n}r(\xi )}\frac{d\xi }{\xi -\zeta }. \end{aligned}$$

Now, we deform the contours ${\hat{\sigma }}$ and ${\tilde{\sigma }}$ appearing in (3.28)–(3.29) back to $\Sigma _{5}$. The integrands in the right-hand side of (3.30) have a non-integrable singularity at 0, and therefore for these integrals we instead deform ${\hat{\sigma }}$ into $\Sigma _{5,-}$ and ${\tilde{\sigma }}$ into $\Sigma _{5,+}$, and we find that

3.30 $$\begin{aligned} {\mathfrak {p}}_1(\zeta ) =&-(c_{4} \ln (s) + c_{7}) \frac{r(\zeta )}{2\pi i} \int _{\Sigma _{5}} \frac{1}{r_{+}(\xi )}\frac{d\xi }{\xi -\zeta }\nonumber \\&+ (a_{3} \ln (s) + a_{4}) \chi _{\text{ int }({\hat{\sigma }} \cup \Sigma _5)}(\zeta ) + ({\tilde{a}}_{3} \ln (s) + {\tilde{a}}_{4}) \chi _{\text{ int }({\tilde{\sigma }} \cup \Sigma _5)}(\zeta ), \end{aligned}$$

3.31a $$\begin{aligned} {\mathfrak {p}}_2(\zeta ) =&-c_{5}\frac{r(\zeta )}{2\pi i} \int _{\Sigma _{5}} \frac{\ln (i\xi )}{r_{+}(\xi )}\frac{d\xi }{\xi -\zeta } - c_{6}\frac{r(\zeta )}{2\pi i} \int _{\Sigma _{5}} \frac{\ln (-i\xi )}{r_{+}(\xi )}\frac{d\xi }{\xi -\zeta }\nonumber \\&+ c_{5} \ln (i\zeta ) \chi _{\text{ int }({\hat{\sigma }} \cup \Sigma _5)}(\zeta ) + c_{6} \ln (-i\zeta ) \chi _{\text{ int }({\tilde{\sigma }} \cup \Sigma _5)}(\zeta ), \end{aligned}$$

3.31b $$\begin{aligned} {\mathfrak {p}}_3(\zeta ) =&\sum _{n=1}^{N} \frac{{\hat{f}}_n}{s^{n\rho }} \frac{r(\zeta )}{2\pi i} \int _{\Sigma _{5,-}} \frac{ 1}{\xi ^{n}r_{-}(\xi )}\frac{d\xi }{\xi -\zeta } -\sum _{n=1}^{N} \frac{{\tilde{f}}_n}{s^{n\rho }} \frac{r(\zeta )}{2\pi i} \int _{\Sigma _{5,+}} \frac{ 1}{\xi ^{n}r_{+}(\xi )}\frac{d\xi }{\xi -\zeta }\nonumber \\&+ \sum _{n=1}^{N} \frac{{\hat{f}}_n}{{(s^{\rho }\zeta )^n}}\chi _{\text{ int }({\hat{\sigma }} \cup \Sigma _5)}(\zeta ) + \sum _{n=1}^{N} \frac{{\tilde{f}}_n}{{(s^{\rho }\zeta )^n}} \chi _{\text{ int }({\tilde{\sigma }} \cup \Sigma _5)}(\zeta ). \end{aligned}$$

Substituting (3.31) into (3.20), the terms proportional to $\chi _{\text{ int }({\hat{\sigma }} \cup \Sigma _5)}$ and $\chi _{\text{ int }({\tilde{\sigma }} \cup \Sigma _5)}$ in the resulting expression for $p(\zeta )$ are given by

3.31c $$\begin{aligned} \chi _{\text{ int }({\hat{\sigma }} \cup \Sigma _5)}(\zeta ) \bigg \{ - {\hat{f}}(\zeta ) + a_{3} \ln (s) + c_{5} \ln (i\zeta ) + a_{4} + \sum _{n=1}^{N} \frac{{\hat{f}}_{n}}{(s^{\rho }\zeta )^n} \bigg \} \end{aligned}$$

and

3.32a $$\begin{aligned} \chi _{\text{ int }({\tilde{\sigma }} \cup \Sigma _5)}(\zeta ) \bigg \{ - {\tilde{f}}(\zeta ) + {\tilde{a}}_{3} \ln (s) + c_{6} \ln (-i\zeta ) + {\tilde{a}}_{4} + \sum _{n=1}^{N} \frac{{\tilde{f}}_{n}}{(s^{\rho }\zeta )^n} \bigg \}, \end{aligned}$$

respectively. Recalling (3.14), we see that the expressions in (3.32) are ${{{\mathcal {O}}}}\big ((s^{\rho } \zeta )^{-N-1}\big )$ as $s \rightarrow +\infty$ uniformly for $\zeta \in {\mathbb {C}}{\setminus } \Sigma _5$ such that $s^{\rho }\zeta \rightarrow \infty$ and $\theta$ in compact subsets of (0, 1]. The expansion (3.24) then follows from (3.27).

The case $\zeta \in {\mathbb {D}}_{\delta /2}(b_1)\cup {\mathbb {D}}_{\delta /2}(b_2)$ only requires minor adaptations of the above arguments, which are similar to those done in the proof of Lemma 3.6, and we omit them here. $\quad \square$

It remains to compute the coefficients in the expansion (3.24) of Lemma 3.8 more explicitly.

#### Lemma 3.9

Let ${\mathcal {R}}$ be defined by (3.3) and let ${\mathcal {G}}_n$ be the *n*th coefficient in the expansion of ${\mathcal {G}}$ given in Proposition 3.1. Then the following identities hold:

3.32b $$\begin{aligned}&\frac{r(\zeta )}{2\pi i} \int _{\Sigma _5} \frac{d\xi }{r_+(\xi )(\xi -\zeta )} = \frac{1}{2}, \end{aligned}$$

3.33 $$\begin{aligned}&r(\zeta ) \bigg \{\frac{c_{5}}{2\pi i} \int _{\Sigma _{5}} \frac{\ln (i\xi )}{r_{+}(\xi )}\frac{d\xi }{\xi -\zeta } + \frac{c_6}{2\pi i} \int _{\Sigma _{5}} \frac{\ln (-i\xi )}{r_{+}(\xi )}\frac{d\xi }{\xi -\zeta } \bigg \} = \frac{c_5\ln (i\zeta )}{2} + \frac{c_6\ln (i\zeta )}{2} - {\mathcal {R}}(\zeta ), \end{aligned}$$

3.34 $$\begin{aligned}&{\hat{f}}_n \frac{r(\zeta )}{2\pi i} \int _{\Sigma _{5,-}} \frac{ 1}{\xi ^{n}r_{-}(\xi )}\frac{d\xi }{\xi -\zeta }-{\tilde{f}}_n \frac{r(\zeta )}{2\pi i} \int _{\Sigma _{5,+}} \frac{ 1}{\xi ^{n}r_{+}(\xi )}\frac{d\xi }{\xi -\zeta } = \frac{{\mathcal {A}}_n(\zeta )}{\zeta ^n}, \qquad n = 1, \dots , N, \end{aligned}$$

where the functions ${\mathcal {A}}_n(\zeta )$ are defined by

3.35 $$\begin{aligned} {\mathcal {A}}_n(\zeta ) = -\frac{{\mathcal {G}}_n}{2}+\frac{ {\mathcal {G}}_n-2{\hat{f}}_n}{2 } \sum _{k=0}^{n-1} \frac{r(\zeta )\zeta ^{k}}{k!} \frac{d^{k}}{d\xi ^{k}}\big (r(\xi )^{-1}\big )\bigg |_{\xi =0-}, \qquad n = 1, \dots , N. \end{aligned}$$

In particular, ${\mathcal {A}}_1(\zeta )$ is given explicitly by (3.4), the functions ${\mathcal {A}}_n(\zeta )$ are holomorphic on ${\mathbb {C}}{\setminus } \Sigma _5$, satisfy ${\mathcal {A}}_n = {{{\mathcal {O}}}}(\zeta ^n)$ as $\zeta \rightarrow \infty$ uniformly for $\theta$ in compact subsets of (0, 1], and depend continuously on $\alpha$ and $\theta$.

#### Proof

Using that $r_{+}(\zeta ) + r_{-}(\zeta ) = 0$ for $\zeta \in \Sigma _{5}$, we obtain

$$\begin{aligned} \int _{\Sigma _5} \frac{d\xi }{r_+(\xi )(\xi -\zeta )}&= \frac{1}{2}\int _{\Sigma _5} \frac{d\xi }{r_+(\xi )(\xi -\zeta )} -\frac{1}{2}\int _{\Sigma _5} \frac{d\xi }{r_-(\xi )(\xi -\zeta )} = \frac{1}{2}\int _{{\mathcal {L}}}\frac{d\xi }{r(\xi )(\xi -\zeta )}, \end{aligned}$$

where ${\mathcal {L}}$ is a clockwise loop which encircles $\Sigma _5$ but which does not encircle $\zeta$. Deforming ${\mathcal {L}}$ to infinity, picking up a residue at $\xi = \zeta$, and using that $\frac{1}{r(\xi )(\xi -\zeta )} = {{{\mathcal {O}}}}(\xi ^{-2})$ as $\xi \rightarrow \infty$, we get

$$\begin{aligned} \int _{\Sigma _5} \frac{d\xi }{r_+(\xi )(\xi -\zeta )} = \frac{\pi i}{r(\zeta )}, \end{aligned}$$

which proves (3.33).

In order to prove (3.34), we first establish the identities

3.36 $$\begin{aligned}&\int _{\Sigma _5} \frac{\ln (i\xi ) d\xi }{r_+(\xi )(\xi -\zeta )} = \pi i \int _{0}^{i\infty } \frac{d\xi }{r(\xi )(\xi -\zeta )} + \pi i \frac{\ln (i\zeta )}{r(\zeta )}, \end{aligned}$$

3.37a $$\begin{aligned}&\int _{\Sigma _5} \frac{\ln (-i\xi ) d\xi }{r_+(\xi )(\xi -\zeta )} = \pi i\int _{0}^{-i\infty }\frac{d\xi }{r(\xi )(\xi -\zeta )} +\pi i \frac{\ln (-i\zeta )}{r(\zeta )}. \end{aligned}$$

**Fig. 7:**
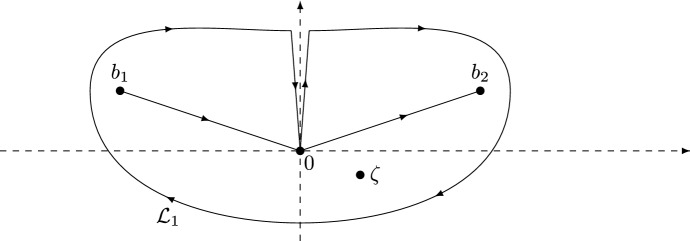
The contour $${\mathcal {L}}_{1}$$

The function $\ln (i\xi )$ is not analytic on $(0,i\infty )$. Therefore, to prove (3.37a), we first open up the contour $\Sigma _5$ and deform it into a loop ${\mathcal {L}}_1$ which encircles $\zeta$ but which avoids the positive imaginary axis as shown in Fig. 7. This gives

$$\begin{aligned} \int _{\Sigma _5} \frac{\ln (i\xi ) d\xi }{r_+(\xi )(\xi -\zeta )}= & {} \frac{1}{2}\int _{\Sigma _5} \frac{\ln (i\xi ) d\xi }{r_+(\xi )(\xi -\zeta )} - \frac{1}{2}\int _{\Sigma _5} \frac{\ln (i\xi ) d\xi }{r_-(\xi )(\xi -\zeta )} \\= & {} \frac{1}{2}\int _{{\mathcal {L}}_1} \frac{\ln (i\xi ) d\xi }{r(\xi )(\xi -\zeta )} + \pi i \frac{\ln (i\zeta )}{r(\zeta )}. \end{aligned}$$

Deforming the circular part of ${\mathcal {L}}_1$ to infinity and using that $\ln (i\xi )$ jumps by $2\pi i$ across the positive imaginary axis, the identity (3.37a) follows. The identity (3.37b) follows in a similar way by deforming the contour to a loop which encircles $\zeta$ but which does not encircle the negative imaginary axis.

Using that

$$\begin{aligned} \frac{\partial }{\partial \xi } \Bigg ( -\frac{1}{r(\zeta )}\ln \bigg (\frac{b_2(\zeta +\xi )+b_1(-2b_2+\zeta +\xi )-2(\zeta \xi +r(\zeta ) r(\xi ))}{\xi -\zeta } \bigg ) \Bigg )= \frac{1}{r(\xi )(\xi -\zeta )} \end{aligned}$$

and $r_+(0)=i|b_2|=-r_-(0)$, we can write

$$\begin{aligned}&-\ln \bigg (\frac{b_2(\zeta +\xi )+b_1(-2b_2+\zeta +\xi )-2(\zeta \xi +r(\zeta ) r(\xi ))}{\xi -\zeta } \bigg )\Bigg |_{\xi = 0^+}^{i\infty } \\&\quad = \ln \bigg (\frac{|b_2|^2 + i\zeta \mathrm{Im}\,{b_2} - i|b_2| r(\zeta )}{(r(\zeta ) + \zeta - i\mathrm{Im}\,{b_2})\zeta }\bigg ),\\&-\ln \bigg (\frac{b_2(\zeta +\xi )+b_1(-2b_2+\zeta +\xi )-2(\zeta \xi +r(\zeta ) r(\xi ))}{\xi -\zeta } \bigg )\Bigg |_{\xi = 0^-}^{-i\infty } \\&\quad = \ln \bigg (\frac{|b_2|^2 + i\zeta \mathrm{Im}\,{b_2} +i|b_2| r(\zeta )}{(r(\zeta ) + \zeta - i\mathrm{Im}\,{b_2})\zeta }\bigg ), \end{aligned}$$

which shows that

3.37b $$\begin{aligned} {\mathcal {R}}(\zeta ) = -r(\zeta )\bigg ( c_5 \int _{0}^{i \infty } \frac{d\xi }{r(\xi )(\xi -\zeta )} + c_6 \int _{0}^{-i\infty } \frac{d\xi }{r(\xi )(\xi -\zeta )} \bigg ). \end{aligned}$$

The identity (3.34) follows from (3.37) and (3.38).

To prove (3.35), we write $\Sigma _{5,-}=(\Sigma _{5,-} \cup -\Sigma _{5,+}) \cup \Sigma _{5,+}$ and deform $\Sigma _{5,-} \cup -\Sigma _{5,+}$ into a clockwise loop ${\mathcal {L}}$ which encircles $\Sigma _5$ but which does not encircle $\zeta$. Taking into account the fact that ${\mathcal {L}}$ and $\Sigma _{5,-} \cup -\Sigma _{5,+}$ have opposite orientations, this shows that the left-hand side of (3.35) equals

3.38 $$\begin{aligned} -\frac{r(\zeta )}{2\pi i} {\hat{f}}_n \int _{{\mathcal {L}}} \frac{d\xi }{\xi ^n r(\xi )(\xi -\zeta )}-\frac{r(\zeta )}{2\pi i} ({\tilde{f}}_n-{\hat{f}}_n) \int _{\Sigma _{5,+}} \frac{d\xi }{\xi ^n r_+(\xi )(\xi -\zeta )}. \end{aligned}$$

Deforming ${\mathcal {L}}$ to infinity (picking up a residue contribution from $\zeta$ but no contribution from infinity), we can write the first term in (3.39) as

3.39 $$\begin{aligned} -\frac{r(\zeta )}{2\pi i} {\hat{f}}_n \int _{{\mathcal {L}}} \frac{d\xi }{\xi ^n r(\xi )(\xi -\zeta )} = -\frac{{\hat{f}}_n }{\zeta ^{n}}. \end{aligned}$$

On the other hand, using the jump relation of *r* on $\Sigma _5$ to open up the contour $\Sigma _{5,+}$ and picking up a residue contribution from $\xi = 0$, we can write the second term in (3.39) as

3.40 $$\begin{aligned}&\qquad -\,\frac{r(\zeta )}{2\pi i} ({\tilde{f}}_n-{\hat{f}}_n) \int _{\Sigma _{5,+}} \frac{d\xi }{\xi ^n r_+(\xi )(\xi -\zeta )} \nonumber \\&\quad =\, -\frac{r(\zeta )}{4\pi i} ({\tilde{f}}_n-{\hat{f}}_n) \int _{{\mathcal {L}}} \frac{d\xi }{\xi ^n r(\xi )(\xi -\zeta )}\nonumber \\&\qquad -\, ({\tilde{f}}_n-{\hat{f}}_n)\frac{r(\zeta )}{2(n-1)!} \frac{d^{n-1}}{d\xi ^{n-1}} \bigg (\frac{1}{r(\xi )(\xi -\zeta )}\bigg )\bigg |_{\xi = 0^-}. \end{aligned}$$

Deforming ${\mathcal {L}}$ to infinity (picking up a residue contribution from $\zeta$ but no contribution from infinity), the first term on the right-hand side of (3.41) can be written as

3.41 $$\begin{aligned} -\frac{r(\zeta )}{4\pi i} ({\tilde{f}}_n-{\hat{f}}_n) \int _{{\mathcal {L}}} \frac{d\xi }{\xi ^n r(\xi )(\xi -\zeta )}=-\frac{({\tilde{f}}_n-{\hat{f}}_n)}{2 \zeta ^{n}}. \end{aligned}$$

Substituting (3.40)–(3.42) into (3.39) and recalling that ${\mathcal {G}}_n ={\hat{f}}_n+{\tilde{f}}_n$, it follows that the left-hand side of (3.35) equals

$$\begin{aligned}&-\frac{{\hat{f}}_n }{\zeta ^{n}}-\frac{({\tilde{f}}_n-{\hat{f}}_n)}{2 \zeta ^{n}}-({\tilde{f}}_n-{\hat{f}}_n)\frac{r(\zeta )}{2(n-1)!} \frac{d^{n-1}}{d\xi ^{n-1}} \bigg (\frac{1}{r(\xi )(\xi -\zeta )}\bigg )\bigg |_{\xi = 0^-} \\&\quad = -\frac{{\mathcal {G}}_n}{2\zeta ^{n}}+\frac{ {\tilde{f}}_n-{\hat{f}}_n}{2\zeta ^n } \sum _{k=0}^{n-1} \frac{r(\zeta )\zeta ^{k}}{k!}\frac{d^{k}}{d\xi ^{k}}\big (r^{-1}(\xi )\big )\bigg |_{\xi =0^-} = \frac{{\mathcal {A}}_n(\zeta )}{\zeta ^n}, \end{aligned}$$

which proves (3.35). Recalling that ${\mathcal {G}}_1= -ic_8$, ${\hat{f}}_1=-i\frac{3\alpha ^2-1}{24}$, and $\tilde{f_1}=-ic_8-{\hat{f}}_1$ and using that $r_-(0)=-i|b_2|$, we find the explicit expression (3.4) for the coefficient ${\mathcal {A}}_1(\zeta )$. Since $r(\zeta ) \sim \zeta$ as $\zeta \rightarrow \infty$, we see from (3.36) that ${\mathcal {A}}_n(\zeta )$ is analytic for $\zeta \in {\mathbb {C}}{\setminus } \Sigma _5$ and of order ${{{\mathcal {O}}}}(\zeta ^n)$ as $\zeta \rightarrow \infty$. Furthermore, since ${\hat{f}}_n$ and ${\mathcal {G}}_n$ depend continuously on $\alpha$ and $\theta$, so does ${\mathcal {A}}_n(\zeta )$. $\quad \square$

The asymptotic formula (3.2) for $p(\zeta )$ follows by substituting the identities of Lemma 3.9 into the expansion (3.24) of Lemma 3.8. This completes the proof of Proposition 3.2.

## Asymptotics of $R(\zeta )$

In this section, we establish the existence of an expansion to all orders of $R(\zeta )$ as $s \rightarrow +\infty$ and derive an explicit expression for the first coefficient $R^{(1)}(\zeta )$ of this expansion. By expanding $R^{(1)}(\zeta )$ as $\zeta \rightarrow \infty$, we can compute the matrix $R_1^{(1)}$ defined by (2.34). Even though only the (2, 2) entry of $R_1^{(1)}$ is needed to compute *c*, we compute the full matrix $R^{(1)}(\zeta )$, because it will be needed later for the evaluation of *C*. The results are summarized in the following proposition.

### Proposition 4.1

(Asymptotics of *R*). Let $N \ge 1$ be an integer. Suppose $\alpha > -1$ and $0 < \theta \le 1$. Let $\{c_j\}_1^8$ and $b_1, b_2$ be the complex constants expressed in terms of the parameters $\alpha$ and $\theta$ by (2.4) and (2.7), respectively.

There exist holomorphic functions $R^{(n)}:{\mathbb {C}}{\setminus } (\partial {\mathbb {D}}_\delta (b_1)\cup \partial {\mathbb {D}}_\delta (b_2)) \rightarrow {\mathbb {C}}$, $n=1,\ldots N$, such that the matrix valued function $R(\zeta )$ defined in (2.32) admits the expansion

3.42 $$\begin{aligned} R(\zeta ) = I+\sum _{n=1}^{N} \frac{R^{(n)}(\zeta )}{s^{n\rho }} + {{{\mathcal {O}}}}\bigg (\frac{1}{s^{(N+1)\rho }(1+ |\zeta |)}\bigg ), \qquad s\rightarrow +\infty , \end{aligned}$$

uniformly for $\zeta \in {\mathbb {C}}{\setminus } \Gamma _R$ and $\theta$ in compact subsets of (0, 1]. As $\zeta \rightarrow \infty$, $R^{(n)}(\zeta ) = O(\zeta ^{-1})$ for each $n = 1, \dots , N$. The expansion (4.1) can be differentiated with respect to $\zeta$ in the sense that

4.1 $$\begin{aligned} R'(\zeta ) = \sum _{n=1}^{N} \frac{R^{(n)\prime }(\zeta )}{s^{n\rho }} + {{{\mathcal {O}}}}\bigg (\frac{1}{s^{(N+1)\rho }(1+ |\zeta |)^2}\bigg ), \qquad s\rightarrow +\infty , \end{aligned}$$

uniformly for $\zeta \in {\mathbb {C}}{\setminus } \Gamma _R$ and $\theta$ in compact subsets of (0, 1]. For any $N \ge 1$,

4.2 $$\begin{aligned} R_{+}^{-1}(\zeta )R_{+}'(\zeta )-R_{-}^{-1}(\zeta )R_{-}'(\zeta ) = {{{\mathcal {O}}}}\bigg (\frac{1}{s^{N\rho }(1+ |\zeta |)^N}\bigg ) \qquad \text{ as } s \rightarrow +\infty , \end{aligned}$$

uniformly for $\zeta \in \cup _{i=1}^4 \Sigma _i {\setminus } ({\mathbb {D}}_\delta (b_1)\cup {\mathbb {D}}_\delta (b_2))$ and $\theta$ in compact subsets of (0, 1]. Moreover, the first coefficient $R^{(1)}(\zeta )$ is given explicitly by

4.3 $$\begin{aligned} R^{(1)}(\zeta )=\frac{A}{\zeta -b_1}+\frac{B}{(\zeta -b_1)^2} - \frac{{{\bar{A}}}}{\zeta -b_2} + \frac{{{\bar{B}}}}{(\zeta -b_2)^2}, \qquad \zeta \in {\mathbb {C}}{\setminus } ({\overline{{\mathbb {D}}_\delta (b_1)}} \cup {\overline{{\mathbb {D}}_\delta (b_2)}}), \end{aligned}$$

where the constant matrices *A* and *B* are defined by

4.4 $$\begin{aligned} A = \begin{pmatrix} A_{1,1} &{} A_{1,2} \\ A_{2,1} &{} A_{2,2} \end{pmatrix}, \qquad B = - \frac{5b_1}{48(c_1+c_2)} \begin{pmatrix} i &{} 1 \\ 1 &{} -i \end{pmatrix}, \end{aligned}$$

with

$$\begin{aligned} A_{1,1}&=\frac{3 \mathrm{Im}\,b_2 +2i\mathrm{Re}\,b_2-12(|b_2|(c_5-c_6)(c_5+c_6)+(c_5^2+c_6^2)\mathrm{Im}\,b_2+2ic_5c_6 \mathrm{Re}\,b_2)}{48 (c_1+c_2) \mathrm{Re}\,b_2}, \\ A_{1,2}&=\frac{4i(3|b_2|(c_5-c_6)(1+c_5+c_6)+\mathrm{Im}\,b_2 + 3(c_5+c_5^2+c_6+c_6^2)\mathrm{Im}\,b_2)}{48(c_1+c_2)\mathrm{Re}\,b_2} \\&\quad -\frac{(5+12c_6+12c_5(1+2c_6))}{48(c_1+c_2)}, \\ A_{2,1}&= \frac{12i|b_2|(c_5-c_6)(-1+c_5+c_6)+4i(1+3(c_5-1)c_5+3(c_6-1)c_6) \mathrm{Im}\,b_2}{48(c_1+c_2)\mathrm{Re}\,b_2} \\&\quad +\frac{-5+12(c_5+c_6-2c_5c_6)}{48(c_1+c_2)}, \\ A_{2,2}&=-A_{1,1}. \end{aligned}$$

In particular, the matrix $R_1^{(1)}$ in (2.34) is given by $R_1^{(1)} = A-{\bar{A}}$ and has (2, 2) element

4.5 $$\begin{aligned} (R_1^{(1)})_{2,2} = -i\frac{1- 12c_5c_6}{12(c_1+c_2)}. \end{aligned}$$

The remainder of this section is devoted to the proof of Proposition 4.1. We start by obtaining an asymptotic expansion of the jump matrix $J_R$ for the RH problem satisfied by *R*.

### Asymptotics of $J_R$

We recall from (2.32) that *R* is given by

$$\begin{aligned} R(\zeta ) = e^{p_0 \sigma _3} S(\zeta ) \times {\left\{ \begin{array}{ll} P(\zeta )^{-1} e^{-p_0 \sigma _3}, &{}\text { if } \zeta \in {\mathbb {D}}_\delta (b_1)\cup {\mathbb {D}}_\delta (b_2), \\ P^{\infty }(\zeta )^{-1} e^{-p_0 \sigma _3}, &{}\text { elsewhere}, \end{array}\right. } \end{aligned}$$

where *S*, *P*, and $P^{\infty }$ have been defined in Sect. 2. For $\zeta \in \Gamma _R$, *R* satisfies the jump condition $R_+ = R_- J_R$ where

4.6 $$\begin{aligned} J_R(\zeta )= {\left\{ \begin{array}{ll} e^{p_0 \sigma _3} P_-^\infty (\zeta )J_S(\zeta )P_+^\infty (\zeta )^{-1} e^{-p_0 \sigma _3}, &{}\text { if } \zeta \in \Gamma _R {\setminus } (\partial {\mathbb {D}}_\delta (b_1)\cup \partial {\mathbb {D}}_\delta (b_2)), \\ e^{p_0 \sigma _3}P(\zeta )P^\infty (\zeta )^{-1} e^{-p_0 \sigma _3}, &{}\text { if } \zeta \in \partial {\mathbb {D}}_\delta (b_1)\cup \partial {\mathbb {D}}_\delta (b_2), \end{array}\right. } \end{aligned}$$

and $J_S$ denotes the jump matrix for *S* (see [12, Eq. (3.21)]):

4.7 $$\begin{aligned} J_S(\zeta ) = {\left\{ \begin{array}{ll} \begin{pmatrix} 1 &{} -{\mathcal {G}}(\zeta ) e^{s^\rho (2g(\zeta ) - i h(\zeta ) + \ell )} \\ 0 &{} 1 \end{pmatrix}, &{} \zeta \in \Sigma _1 \cup \Sigma _2, \\ \begin{pmatrix} 1 &{} 0 \\ {\mathcal {G}}(\zeta )^{-1} e^{-s^\rho (2g(\zeta ) - i h(\zeta ) + \ell )} &{} 1 \end{pmatrix}, &{} \zeta \in \Sigma _3 \cup \Sigma _4, \\ \begin{pmatrix} e^{-s^\rho (g_+(\zeta ) - g_-(\zeta ))} &{} -{\mathcal {G}}(\zeta ) \\ {\mathcal {G}}(\zeta )^{-1} &{} 0 \end{pmatrix},&\zeta \in \Sigma _5. \end{array}\right. } \end{aligned}$$

The symmetries

$$\begin{aligned}&{\mathcal {G}}(\zeta )=\overline{{\mathcal {G}}(-{\bar{\zeta }})}, \quad g(\zeta )=\overline{g(-{\bar{\zeta }})}, \quad h(\zeta ) = -\overline{h(-{\bar{\zeta }})}, \quad \\&P^\infty (\zeta )=\overline{P^\infty (-{\bar{\zeta }})}, \quad P(\zeta )=\overline{P(-{\bar{\zeta }})}, \end{aligned}$$

together with the fact that $p_0, \ell \in {\mathbb {R}}$ imply that the jump matrix $J_R$ obeys the symmetry

4.8 $$\begin{aligned} J_R(\zeta )=\overline{J_R(-{\bar{\zeta }})}, \qquad \zeta \in \Gamma _R. \end{aligned}$$

From (4.9) and the symmetry of the behavior of *R* near the points of self-intersection of $\Gamma _{R}$ and infinity, as well as the uniqueness of the solution of the RH problem for *R*, we conclude that *R* obeys the symmetry

$$\begin{aligned} R(\zeta ) = \overline{R(-{\overline{\zeta }})}, \qquad \zeta \in {\mathbb {C}}{\setminus } \Gamma _{R}. \end{aligned}$$

Note that $|b_2| = (1+\theta )\theta ^{\frac{1-\theta }{1+\theta }}$ by (2.7) and (2.8), so that $b_1$ and $b_2$ approach the origin only as $\theta \downarrow 0$.

The next lemma establishes the existence of an asymptotic expansion to all orders of the jump matrix $J_R(\zeta )$ as $s \rightarrow + \infty$.

#### Lemma 4.2

(Asymptotics of $J_R$). Let $N\ge 1$ be an integer and let $\alpha > -1$. There exists an asymptotic expansion

4.9 $$\begin{aligned} J_R(\zeta ) = I + \sum _{n=1}^N \frac{J_R^{(n)}(\zeta )}{s^{n \rho }} + {{{\mathcal {O}}}}\bigg ( \frac{1}{s^{(N+1)\rho }}\bigg ) \qquad \text{ as } s \rightarrow +\infty , \end{aligned}$$

where the error term is uniform for $\zeta \in \partial {\mathbb {D}}_\delta (b_1)\cup \partial {\mathbb {D}}_\delta (b_2)$ and for $\theta$ in compact subsets of (0, 1], and

$$\begin{aligned} J_R^{(n)}: ({\overline{{\mathbb {D}}_\delta (b_1)}} \cup {\overline{{\mathbb {D}}_\delta (b_2)}} ) {\setminus } \{b_{1},b_{2}\} \rightarrow {\mathbb {C}}^{2\times 2}, \quad n = 1, \dots , N, \end{aligned}$$

are holomorphic functions which satisfy the symmetry

4.10 $$\begin{aligned} J_R^{(n)}(\zeta )=\overline{J_R^{(n)}(-{\bar{\zeta }})}, \qquad \zeta \in {\overline{{\mathbb {D}}_\delta (b_1)}} \cup {\overline{{\mathbb {D}}_\delta (b_2)}}, \ n = 1, \dots , N. \end{aligned}$$

For $\zeta \in {\overline{{\mathbb {D}}_\delta (b_1)}}$, $J_{R}^{(1)}(\zeta )$ is explicitly given by

4.11 $$\begin{aligned} J_R^{(1)}(\zeta )&\; = \frac{1}{72q(\zeta )}Q^\infty (\zeta )e^{\frac{{\mathcal {R}}(\zeta )}{2}\sigma _3}\begin{pmatrix} -1 &{} -6i \\ -6i &{} 1 \end{pmatrix} e^{-\frac{{\mathcal {R}}(\zeta )}{2}\sigma _3} \big ( Q^\infty (\zeta ) \big )^{-1}, \end{aligned}$$

i.e.,

4.12 $$\begin{aligned} J_R^{(1)}(\zeta )_{1,1}&=-J_R^{(1)}(\zeta )_{2,2}, \nonumber \\ J_R^{(1)}(\zeta )_{2,1}&= \frac{1}{72 q(\zeta )}\bigg ( i\frac{-\mathrm{Re}\,b_2+6(i\mathrm{Im}\,b_2-\zeta )\cosh ({\mathcal {R}}(\zeta ))}{r(\zeta )} +6i\sinh ({\mathcal {R}}(\zeta )) \bigg ), \nonumber \\ J_R^{(1)}(\zeta )_{1,2}&=\frac{1}{72 q(\zeta )}\bigg (i \frac{-\mathrm{Re}\,b_2+6(i\mathrm{Im}\,b_2-\zeta )\cosh ({\mathcal {R}}(\zeta ))}{r(\zeta )} -6i\sinh ({\mathcal {R}}(\zeta )) \bigg ), \nonumber \\ J_R^{(1)}(\zeta )_{2,2}&=\frac{1}{72 q(\zeta )}\bigg ( \frac{-(i\mathrm{Im}\,b_2-\zeta )+6 \mathrm{Re}\,(b_2)\cosh ({\mathcal {R}}(\zeta ))}{r(\zeta )} \bigg ). \end{aligned}$$

#### Proof

Substituting the expressions (2.18), (2.27) and (2.30) for $P^\infty$, *P*, and *E* into the expression (4.7) for $J_{R}$ on $\partial {\mathbb {D}}_\delta (b_1)$, we find

4.13 $$\begin{aligned} J_{R}(\zeta ) =&\; Q^{\infty }(\zeta ) e^{p(\zeta )\sigma _{3}} {\mathcal {G}}(\zeta )^{\frac{\sigma _{3}}{2}} \begin{pmatrix} 1 &{} i \\ 1 &{} -i \end{pmatrix}^{-1} \Big ( s^{\frac{2}{3}\rho }f(\zeta ) \Big )^{\frac{\sigma _{3}}{4}} \nonumber \\&\times A_{k}\big ( s^{\frac{2}{3}\rho }f(\zeta ) \big )e^{-s^{\rho }q(\zeta )\sigma _{3}}{\mathcal {G}}(\zeta )^{-\frac{\sigma _{3}}{2}}e^{-p(\zeta )\sigma _{3}}Q^{\infty }(\zeta )^{-1}, \qquad \zeta \in \partial {\mathbb {D}}_\delta (b_1). \end{aligned}$$

We can extend the asymptotic formula (2.25) for $A_{k}(\zeta )$ to all orders as follows. The Airy function admits the following well-known uniform asymptotic expansions to all orders (see [27, Eqs. 9.7.5 and 9.7.6]):

4.14 $$\begin{aligned} \mathrm{Ai}(\zeta )&\sim \frac{e^{-\frac{2}{3}\zeta ^\frac{3}{2}}}{2\sqrt{\pi } \zeta ^{1/4}} \sum _{l=0}^\infty \frac{(-1)^lu_l}{(\frac{2}{3}\zeta ^{3/2})^l},&\mathrm{Ai}'(\zeta )\sim -\frac{e^{-\frac{2}{3}\zeta ^\frac{3}{2}}\zeta ^{1/4}}{2\sqrt{\pi } } \sum _{l=0}^\infty \frac{(-1)^lv_l}{(\frac{2}{3}\zeta ^{3/2})^l}, \end{aligned}$$

as $\zeta \rightarrow \infty$, $|\arg \zeta |<\pi - \delta '$ for any $\delta ' >0$, where the coefficients $\{u_l, v_l\}_{l=0}^{\infty }$ are given by

$$\begin{aligned}&u_{l} = \frac{(6l-5)(6l-3)(6l-1)}{(2l-1)216l}u_{l-1},&l \ge 1, \\&v_{l} = \frac{6l+1}{1-6l}u_{l},&l \ge 1, \end{aligned}$$

and $u_{0} = v_{0} = 1$. Substituting the asymptotic expansions (4.15) into (2.22)–(2.24), it follows that, for $k = 1,2,3$,

4.15 $$\begin{aligned} A_{k}(\zeta )&\sim \zeta ^{-\frac{\sigma _3}{4}}\sum _{l=0}^{\infty }\frac{1}{(\frac{2}{3}\zeta ^{3/2})^l} \begin{pmatrix} u_l &{} i(-1)^lu_l \\ v_l &{} -i(-1)^lv_l \end{pmatrix}e^{\frac{2}{3}\zeta ^{3/2}\sigma _3} \nonumber \\&\sim \zeta ^{-\frac{\sigma _3}{4}} \begin{pmatrix} 1 &{} i \\ 1 &{} -i \end{pmatrix} \left( I + \sum _{l=1}^{\infty } \frac{1}{(\frac{2}{3}\zeta ^{3/2})^l} \frac{u_{l}}{1-6l}\begin{pmatrix} 1 &{} (-1)^{l+1}6 l i \\ 6 l i &{} (-1)^{l} \end{pmatrix} \right) e^{\frac{2}{3}\zeta ^{3/2}\sigma _3} \end{aligned}$$

uniformly in the sector $S_k$ defined in (2.26), where the branches of complex powers are as in (2.25).

Next note that by combining the expansions (3.1) and (3.2), we find

4.16 $$\begin{aligned} e^{p(\zeta )\sigma _3}{\mathcal {G}}(\zeta )^{\frac{\sigma _3}{2}}&= \exp \left( \left[ \frac{{\mathcal {R}}(\zeta )}{2} + \sum _{n=1}^{N} \frac{\tilde{{\mathcal {A}}}_n(\zeta )}{(s^\rho \zeta )^n}+{{{\mathcal {O}}}}\bigg ( \frac{1}{(s^\rho \zeta )^{N+1}}\bigg ) + {{{\mathcal {O}}}}\bigg ( \frac{1}{s^{(N+1)\rho }}\bigg )\right] \sigma _{3} \right) \end{aligned}$$

as $s \rightarrow +\infty$ uniformly for $\theta$ in compact subsets of (0, 1] and uniformly for $\zeta \in {\mathbb {C}}{\setminus } \Sigma _{5}$ such that $s^{\rho }\zeta \rightarrow \infty$, $|\arg (\zeta ) - \tfrac{\pi }{2}| > \epsilon$ and $|\arg (\zeta ) + \tfrac{\pi }{2}| > \epsilon$ for any fixed $\epsilon > 0$, where ${\mathcal {R}}(\zeta )$ is defined by (3.3) and $\tilde{{\mathcal {A}}}_n(\zeta )$ are holomorphic functions of $\zeta \in {\mathbb {C}}{\setminus } \Sigma _{5}$ defined by

$$\begin{aligned}&\tilde{{\mathcal {A}}}_n(\zeta ) = {\mathcal {A}}_n(\zeta ) +\frac{{\mathcal {G}}_n}{2}, \qquad n \ge 1. \end{aligned}$$

Utilizing the large *s* expansions (4.16) and (4.17) in the expression (4.14) for $J_R(\zeta )$, we obtain

4.17 $$\begin{aligned} J_R(\zeta )=&\; I + Q^\infty (\zeta )\exp \left( \left[ \frac{{\mathcal {R}}(\zeta )}{2} + \sum _{n=1}^{N} \frac{\tilde{{\mathcal {A}}}_n(\zeta )}{(s^\rho \zeta )^n} +{{{\mathcal {O}}}}\big ( s^{-(N+1)\rho }\big ) \right] \sigma _{3} \right) \nonumber \\&\times \left( \sum _{l=1}^{N} \frac{1}{(s^{\rho } q(\zeta ))^l} \frac{u_{l}}{1-6l}\begin{pmatrix} 1 &{} (-1)^{l+1}6 l i \\ 6 l i &{} (-1)^{l} \end{pmatrix} + {{{\mathcal {O}}}}\big ((s^\rho q(\zeta ))^{-(N+1)}\big ) \right) \nonumber \\&\times \exp \left( - \left[ \frac{{\mathcal {R}}(\zeta )}{2} + \sum _{n=1}^{N} \frac{\tilde{{\mathcal {A}}}_n(\zeta )}{(s^\rho \zeta )^n}+{{{\mathcal {O}}}}\big ( s^{-(N+1)\rho }\big ) \right] \sigma _{3} \right) Q^\infty (\zeta )^{-1}, \end{aligned}$$

as $s \rightarrow +\infty$ uniformly for $\zeta \in \partial {\mathbb {D}}_\delta (b_1)$ and $\theta$ in compact subsets of (0, 1]. The error term ${{{\mathcal {O}}}}((s^\rho q(\zeta ))^{-(N+1)})$ can be replaced by ${{{\mathcal {O}}}}(s^{-(N+1)\rho })$, because $|q(\zeta )|$ is uniformly bounded away from zero on $\partial {\mathbb {D}}_\delta (b_1)$ by (2.31). It follows that $J_R$ admits an expansion of the form (4.10) with coefficients $J_R^{(n)}(\zeta )$, $n = 1, \dots , N$, which can be computed explicitly from (4.18) by straightforward algebra. In particular, this gives the explicit expression (4.12) for the first coefficient $J_{R}^{(1)}(\zeta )$; using the definition (2.18) of $Q^\infty (\zeta )$ and the fact that $r(\zeta ) = (\zeta -b_2)\gamma (\zeta )^2$, the relations in (4.13) follow.

We finally show that $J_R^{(n)}(\zeta )$, $n = 1, \dots , N$, are analytic functions of $\zeta \in {\overline{{\mathbb {D}}_\delta (b_1)}} {\setminus } \{b_1\}$. This will complete the proof of the lemma because the expansion (4.10) for $\zeta \in \partial {\mathbb {D}}_\delta (b_2)$ and the symmetry (4.11) then follow from (4.9). Clearly, the coefficients $J_R^{(n)}$ are analytic on ${\overline{{\mathbb {D}}_\delta (b_1)}} {\setminus } \Sigma _{5}$. In fact, it follows from (4.18) that they have no jump across $\Sigma _{5}$, because for $\zeta \in \Sigma _{5}$ we have

$$\begin{aligned}&{\mathcal {R}}_{+}(\zeta ) + {\mathcal {R}}_{-}(\zeta ) = 0, \qquad q_{+}(\zeta ) + q_{-}(\zeta ) = 0, \qquad Q_{+}^{\infty }(\zeta ) = Q_{-}^{\infty }(\zeta )\begin{pmatrix} 0 &{} -1 \\ 1 &{} 0 \end{pmatrix}, \\&\tilde{{\mathcal {A}}}_{n,+}(\zeta ) + \tilde{{\mathcal {A}}}_{n,-}(\zeta ) = 0 \qquad \text{ for } \text{ all } n \ge 1, \\&\frac{q_{-}(\zeta )^{l}}{q_{+}(\zeta )^{l}} \begin{pmatrix} 1 &{} (-1)^{l+1}6 l i \\ 6 l i &{} (-1)^{l} \end{pmatrix}^{-1} \begin{pmatrix} 0 &{} -1 \\ 1 &{} 0 \end{pmatrix} \begin{pmatrix} 1 &{} (-1)^{l+1}6 l i \\ 6 l i &{} (-1)^{l} \end{pmatrix} = \begin{pmatrix} 0 &{} -1 \\ 1 &{} 0 \end{pmatrix} \quad \text{ for } \text{ all } l \ge 1. \end{aligned}$$

This shows that the coefficients $J_R^{(n)}(\zeta )$ are analytic on ${\overline{{\mathbb {D}}_\delta (b_1)}} {\setminus } \{b_1\}$ (note however that the $J_R^{(n)}(\zeta )$ may have poles at $b_1$ because $q(\zeta )\rightarrow 0$ as $\zeta \rightarrow b_1$). $\quad \square$

### Existence of an expansion to all orders

In the following lemma, we show that the $L^{p}$ norm of $w_R := J_R-I$ on $\Gamma _{R}$ is small for any $1 \le p \le \infty$ uniformly for $\theta$ in compact subsets of (0, 1], whenever *s* is large enough.

#### Lemma 4.3

(Estimates of $w_R$). Let $N \ge 1$ be an integer and let *K* be a compact subset of (0, 1]. For each $1 \le p \le \infty$ and each $M \ge 0$, there exist positive constants $C'$ and $c'$ such that the following estimates hold:

4.18 $$\begin{aligned} \sup _{\theta \in K} \left\Vert w_R - \sum _{n=1}^{N}\frac{J_R^{(n)}}{s^{n\rho }} \right\Vert _{L^p(\partial {\mathbb {D}}_\delta (b_1)\cup \partial {\mathbb {D}}_\delta (b_2))}&\le \frac{C'}{s^{(N+1)\rho }}, \end{aligned}$$

4.19a $$\begin{aligned} \sup _{\theta \in K} \Vert (1+ |\zeta |)^M w_R \Vert _{L^p(\Gamma _R {\setminus } (\partial {\mathbb {D}}_\delta (b_1)\cup \partial {\mathbb {D}}_\delta (b_2)))}&\le C'e^{-c's^\rho }, \end{aligned}$$

4.19b $$\begin{aligned} \sup _{\theta \in K} \Vert (1+ |\zeta |)^M \partial _\zeta w_R \Vert _{L^\infty (\cup _{i=1}^4 \Sigma _i {\setminus } ( {\mathbb {D}}_\delta (b_1)\cup {\mathbb {D}}_\delta (b_2)))}&\le C'e^{-c's^\rho }. \end{aligned}$$

#### Proof

In this proof, $c'$ and $C'$ denote generic positive constant which may change within a computation. Since $\partial {\mathbb {D}}_\delta (b_1)\cup \partial {\mathbb {D}}_\delta (b_2)$ is compact, the estimate (4.19a) follows from Lemma 4.2.

Assume $\zeta \in (\Sigma _1 \cup \Sigma _2){\setminus } ({\mathbb {D}}_\delta (b_1)\cup {\mathbb {D}}_\delta (b_2))$. By (2.18), (4.7), and (4.8), we have

4.19c $$\begin{aligned} w_R(\zeta )=Q^{\infty }(\zeta ) \begin{pmatrix} 0 &{} -e^{2p(\zeta )}{\mathcal {G}}(\zeta ) e^{s^{\rho }(2g(\zeta ) - i h(\zeta ) + \ell )} \\ 0 &{} 0 \end{pmatrix}Q^{\infty }(\zeta )^{-1}. \end{aligned}$$

We see from the expression (3.3) for ${\mathcal {R}}(\zeta )$ that $|\mathrm{Re}\,{{\mathcal {R}}(\zeta )}| = {{{\mathcal {O}}}}(\ln |\zeta |)$ as $\zeta \rightarrow \infty$ and hence

$$\begin{aligned} |e^{{\mathcal {R}}(\zeta )/2}| = {{{\mathcal {O}}}}\big ((1 + |\zeta |)^{C'}\big ) \end{aligned}$$

uniformly for $\zeta \in \Sigma _1\cup \Sigma _2$ and $\theta \in K$. It then follows from Propositions 3.1 and 3.2 (see (4.17)) that

4.20 $$\begin{aligned} \big |e^{2p(\zeta )}{\mathcal {G}}(\zeta )\big | = {{{\mathcal {O}}}}\big ((1 + |\zeta |)^{C'}\big ) \end{aligned}$$

uniformly for $\zeta \in \Sigma _1\cup \Sigma _2$, $\theta \in K$, and $s \ge 1$. Furthermore, a minor modification of the proof of [12, Lemma 3.1]^6^ together with the fact that $h(\zeta ) = {{{\mathcal {O}}}}(\zeta \ln \zeta )$ as $\zeta \rightarrow \infty$ yields

4.21 $$\begin{aligned} \mathrm{Re}\,(2g(\zeta ) - i h(\zeta ) + \ell )< -c' |\zeta | <0, \qquad \zeta \in \Sigma _{1} \cup \Sigma _{2}, \end{aligned}$$

for some $c' >0$ for all $\theta \in K$. Equations (4.20), (4.21), and (4.22) imply that, for any $M \ge 0$,

4.22 $$\begin{aligned} \sup _{\theta \in K} \Vert (1+ |\zeta |)^M w_R \Vert _{L^p((\Sigma _{1}\cup \Sigma _{2}) {\setminus } ({\mathbb {D}}_\delta (b_1)\cup {\mathbb {D}}_\delta (b_2)))} \le C'e^{-c's^\rho }, \end{aligned}$$

and a similar argument shows that

4.23 $$\begin{aligned} \sup _{\theta \in K} \Vert (1+ |\zeta |)^M w_R \Vert _{L^p((\Sigma _{3}\cup \Sigma _{4}) {\setminus } ({\mathbb {D}}_\delta (b_1)\cup {\mathbb {D}}_\delta (b_2)))} \le C'e^{-c's^\rho }. \end{aligned}$$

Let now $\zeta \in \Sigma _5 {\setminus } ({\mathbb {D}}_\delta (b_1)\cup {\mathbb {D}}_\delta (b_2))$. Then, from (4.7) and (4.8), we obtain

4.24 $$\begin{aligned} J_{R}(\zeta ) = e^{p_0 \sigma _3} P_-^\infty (\zeta )\begin{pmatrix} e^{-s^\rho (g_+(\zeta )-g_-(\zeta ))}&{} -{\mathcal {G}}(\zeta ) \\ {\mathcal {G}}(\zeta )^{-1} &{} 0 \end{pmatrix}P_+^\infty (\zeta )^{-1}e^{-p_0 \sigma _3}. \end{aligned}$$

Using the jump relation of $P^\infty$, given in [12, Eq. (3.47)], and (2.18) this becomes

$$\begin{aligned} J_{R}(\zeta )&=e^{p_0 \sigma _3} P_-^\infty (\zeta )\begin{pmatrix} e^{-s^\rho (g_+(\zeta )-g_-(\zeta ))}&{}- {\mathcal {G}}(\zeta ) \\ {\mathcal {G}}(\zeta )^{-1} &{} 0 \end{pmatrix}\begin{pmatrix} 0&{} {\mathcal {G}}(\zeta ) \\ -{\mathcal {G}}(\zeta )^{-1} &{} 0 \end{pmatrix}P_-^\infty (\zeta )^{-1}e^{-p_0 \sigma _3} \\&=e^{p_0 \sigma _3} P_-^\infty (\zeta )\begin{pmatrix} 1&{}{\mathcal {G}}(\zeta )e^{-s^\rho (g_+(\zeta )-g_-(\zeta ))} \\ 0 &{} 1 \end{pmatrix}P_-^\infty (\zeta )^{-1}e^{-p_0 \sigma _3} \\&=Q_{-}^{\infty }(\zeta ) \begin{pmatrix} 1&{} -e^{2p_-(\zeta )}{\mathcal {G}}(\zeta ) e^{-s^\rho (g_+(\zeta )-g_-(\zeta ))} \\ 0 &{} 1 \end{pmatrix}Q_{-}^{\infty }(\zeta )^{-1}. \end{aligned}$$

Note that $Q^{\infty }_{-}(\zeta )$ and $Q^{\infty }_{-}(\zeta )^{-1}$ are independent of *s* and bounded from above and from below for $\zeta \in \Sigma _{5}{\setminus } ({\mathbb {D}}_\delta (b_1)\cup {\mathbb {D}}_\delta (b_2))$. Combining Proposition 3.2 with [12, Lemma 3.1], we have

$$\begin{aligned} |e^{2p_-(\zeta )}{\mathcal {G}}(\zeta ) e^{-s^\rho (g_+(\zeta )-g_-(\zeta ))}| \le C' e^{-c' s^{\rho }} \qquad \text{ for } \zeta \in \Sigma _{5} \text{ such } \text{ that } s^{\rho }\zeta \ge M \end{aligned}$$

for a certain large constant *M*, uniformly for $\theta \in K$. For $\zeta \in \Sigma _{5}$ such that $s^{\rho }\zeta \le M$, the same estimate still holds; this follows from [12, Lemma 3.1] together with the fact that

$$\begin{aligned} e^{2p_-(\zeta )}{\mathcal {G}}(\zeta ) = {{{\mathcal {O}}}}(1) \qquad \text{ for } \zeta \in \Sigma _{5} \text{ such } \text{ that } s^{\rho }\zeta \le M. \end{aligned}$$

Therefore, we have

$$\begin{aligned} \sup _{\theta \in K} \Vert w_R \Vert _{L^p(\Sigma _{5}{\setminus } ({\mathbb {D}}_\delta (b_1)\cup {\mathbb {D}}_\delta (b_2)))} \le C'e^{-c's^\rho }, \end{aligned}$$

which together with (4.23) and (4.24) finishes the proof of (4.19b).

The estimates (4.23) and (4.24) can clearly be extended to narrow open sectors containing the rays $\cup _{i=1}^4 \Sigma _i {\setminus } ( {\mathbb {D}}_\delta (b_1)\cup {\mathbb {D}}_\delta (b_2))$. The estimate (4.19c) then follows from the analyticity of the jump matrix $J_R$ and Cauchy’s estimate. $\quad \square$

For the reader’s convenience, we recall some well-known facts from the theory of singular integral operators. For a function $u \in L^2(\Gamma _R)$ we define the Cauchy integral ${\mathcal {C}}u$ by

$$\begin{aligned} {\mathcal {C}}u(\zeta ) = \frac{1}{2\pi i} \int _{\Gamma _R} \frac{u(\xi )}{\xi -\zeta }d\xi ,\qquad \zeta \in {\mathbb {C}}\backslash \Gamma _R, \end{aligned}$$

and we denote the non-tangential limits of ${\mathcal {C}}u$ from the left- and right-hand side of $\Gamma _R$ by ${\mathcal {C}}_{+} u$ and ${\mathcal {C}}_{-}u$, respectively. The Cauchy operator ${\mathcal {C}}_{w_R}:L^2(\Gamma _R) \rightarrow L^2(\Gamma _R)$ is defined by

4.25 $$\begin{aligned} {\mathcal {C}}_{w_R}u={\mathcal {C}}_-(w_R u). \end{aligned}$$

This operator is bounded and linear and, assuming that $I-{\mathcal {C}}_{w_R}:L^2(\Gamma _R) \rightarrow L^2(\Gamma _R)$ is invertible, the solution of the RH problem for *R* is given by (see e.g. [16, Section 7])

4.26 $$\begin{aligned} R= I + {\mathcal {C}}(\mu _R w_R), \end{aligned}$$

where

4.27 $$\begin{aligned} \mu _R=I+(I-{\mathcal {C}}_{w_R})^{-1}{\mathcal {C}}_{w_R}(I). \end{aligned}$$

In particular, if ${\mathcal {C}}_{w_R}$ has sufficiently small $L^2$-operator norm, $I-{\mathcal {C}}_{w_R}$ can be inverted in terms of a Neumann series, that is,

4.28 $$\begin{aligned} (I-{\mathcal {C}}_{w_R})^{-1}= \sum _{n=0}^{\infty } {\mathcal {C}}_{w_R}^n. \end{aligned}$$

Hence it follows from Lemma 4.3 and the estimate

4.29 $$\begin{aligned} \Vert {\mathcal {C}}_{w_R} \Vert _{L^2(\Gamma _R)\rightarrow L^2(\Gamma _R)} \le \Vert \mathcal {C_-} \Vert _{L^2(\Gamma _R)\rightarrow L^2(\Gamma _R)} \Vert w_R\Vert _{L^\infty (\Gamma _R)}, \end{aligned}$$

that $I-{\mathcal {C}}_{w_R}$ is invertible for all sufficiently large *s*. Here $\Vert \cdot \Vert _{L^2(\Gamma _R)\rightarrow L^2(\Gamma _R)}$ denotes the operator norm of bounded linear operators $L^2(\Gamma _R)\rightarrow L^2(\Gamma _R)$.

The standard theory for asymptotics of small norm RH problems (see e.g. [16]) together with Lemma 4.3 implies that *R* satisfies (4.1) and that this expansion can be differentiated with respect to $\zeta$. The basic idea here is to combine (4.27)–(4.29) and the expansion (4.10) of the jump matrix. This immediately gives (4.1) uniformly for $\zeta$ bounded away from the contour $\Gamma _R$. For $\zeta$ close to $\Gamma _R$, one uses analyticity of the jump matrix in a neighborhood of $\Gamma _R$ to deform the contour in such a way that $\zeta$ is bounded away from the deformed contour.

Using the jump relation $R_+ = R_- J_R$, the left-hand side of (4.3) can be written for $\zeta \in \cup _{i=1}^4 \Sigma _i {\setminus } ({\mathbb {D}}_\delta (b_1)\cup {\mathbb {D}}_\delta (b_2))$ as

$$\begin{aligned} J_R^{-1}(\zeta ) R_{-}^{-1}(\zeta )R_{-}'(\zeta )J_R(\zeta ) + J_R^{-1}(\zeta )J_R'(\zeta ) - R_{-}^{-1}(\zeta )R_{-}'(\zeta ). \end{aligned}$$

The estimate (4.3) is then a consequence of the estimates (4.19b) and (4.19c) of $J_R(\zeta )$ and $J_R'(\zeta )$, as well as the expansions (4.1) and (4.2) of $R(\zeta )$ and $R'(\zeta )$.

### Explicit expression for $R^{(1)}(\zeta )$

We next derive the explicit expression (4.4) for the coefficient $R^{(1)}(\zeta )$. We have $R=I+{\mathcal {C}}(\mu _R w_R)$ and, by Lemma 4.3 and (4.28),

$$\begin{aligned} w_R(\zeta ) = \frac{J_R^{(1)}(\zeta )}{s^\rho }+ {{{\mathcal {O}}}}(s^{-2\rho } (1+|\zeta |)^{-2}), \qquad \mu _R(\zeta ) = I+{{{\mathcal {O}}}}(s^{-\rho }), \end{aligned}$$

as $s\rightarrow +\infty$, where the error terms are uniform with respect to $\zeta \in \Gamma _R$ and $\theta$ in compact subsets of (0, 1]. This implies

4.30 $$\begin{aligned} R^{(1)}(\zeta )= {\mathcal {C}}J_R^{(1)}(\zeta ) = \frac{1}{2\pi i} \int _{\partial {\mathbb {D}}_\delta (b_1)\cup \partial {\mathbb {D}}_\delta (b_2)} \frac{J_R^{(1)}(\xi )}{\xi -\zeta } d\xi , \end{aligned}$$

where $\partial {\mathbb {D}}_\delta (b_1)$ and $\partial {\mathbb {D}}_\delta (b_2)$ are oriented clockwise. From Lemma 4.2 and (2.31), $J_R^{(1)}$ is analytic on $({\overline{{\mathbb {D}}_\delta (b_1)}} \cup {\overline{{\mathbb {D}}_\delta (b_2)}}){\setminus } \{ b_1,b_2 \}$ with a double pole at each of the points $b_1$ and $b_2$. Furthermore, by (4.11) we have $J_R^{(1)}(\zeta ) = \overline{J_R^{(1)}(-{{\bar{\zeta }}})}$ and hence

4.31 $$\begin{aligned} \frac{1}{2\pi i} \int _{\partial {\mathbb {D}}_\delta (b_1)\cup \partial {\mathbb {D}}_\delta (b_2)} \frac{J_R^{(1)}(\xi )}{\xi -\zeta } d\xi =\frac{1}{2\pi i} \int _{\partial {\mathbb {D}}_\delta (b_1)} \frac{J_R^{(1)}(\xi )}{\xi -\zeta } d\xi +\overline{\frac{1}{2\pi i}\int _{\partial {\mathbb {D}}_\delta (b_1)} \frac{J_R^{(1)}(\xi )}{\xi +{{\bar{\zeta }}}} d\xi }. \end{aligned}$$

By Cauchy’s formula, if $\zeta \notin {\overline{{\mathbb {D}}_\delta (b_1)}}$, we have

4.32 $$\begin{aligned} \frac{1}{2\pi i} \int _{\partial {\mathbb {D}}_\delta (b_1)} \frac{J_R^{(1)}(\xi )}{\xi -\zeta } d\xi&= \frac{A}{\zeta -b_1} + \frac{B}{(\zeta -b_1)^2}, \end{aligned}$$

where the matrices *A* and *B* are defined by

4.33 $$\begin{aligned} A = \frac{d}{d\xi }\big ( (\xi -b_1)^2J_R^{(1)}(\xi )\big )\big |_{\xi =b_1}, \qquad B = \lim _{\xi \rightarrow b_1}\big ( (\xi -b_1)^2 J_R^{(1)}(\xi ) \big ), \end{aligned}$$

so that

$$\begin{aligned} J_R^{(1)}(\xi ) = \frac{B}{(\xi -b_1)^2} + \frac{A}{\xi - b_1} + {{{\mathcal {O}}}}(1), \qquad \xi \rightarrow b_1. \end{aligned}$$

It follows from equations (4.31)–(4.33) that $R^{(1)}(\zeta )$ satisfies (4.4) with *A* and *B* given by (4.34).

We next show that the matrices *A* and *B* can be written as in (4.5). Expanding (2.10) in powers of $\sqrt{\zeta -b_1}$ and recalling the definition (2.28) of *q*, we obtain

4.34 $$\begin{aligned} q(\zeta ) =&-\frac{2}{3} \frac{c_1+c_2}{\sqrt{2}} \frac{\sqrt{\mathrm{Re}\,b_2}}{b_1} (\zeta -b_1)^{\frac{3}{2}} \nonumber \\&+\frac{(c_1 + c_2)(3i\mathrm{Im}\,b_2 +\mathrm{Re}\,b_2) }{30 \sqrt{2}\, b_1^2 \sqrt{\mathrm{Re}\,b_2}}(\zeta -b_1)^{\frac{5}{2}} +{{{\mathcal {O}}}}\big ((\zeta -b_1)^{3}\big ), \qquad \zeta \rightarrow b_1. \end{aligned}$$

Expansion of (3.3) gives

4.35 $$\begin{aligned} {\mathcal {R}}(\zeta ) =-\frac{\sqrt{2}}{\sqrt{\mathrm{Re}\,b_2} } \bigg ( i(c_5+c_6)+(c_5-c_6)\frac{b_2}{|b_2|} \bigg ) \sqrt{\zeta -b_1} + {{{\mathcal {O}}}}\big ( (\zeta -b_1)^{\frac{3}{2}} \big ). \end{aligned}$$

Substituting (4.35) and (4.36) into (4.12) a straightforward calculation shows that *A* and *B* can be written as in (4.5).

Finally, it follows from (4.27) and Lemma 4.3 that the order in which the expansions in *s* and $\zeta$ are computed is irrelevant for the evaluation of the coefficient $R_1^{(1)}$ defined in (2.34). Thus, from (4.1) and (4.4), we have $R_1^{(1)} = A-{\bar{A}}$ and a straightforward computation then gives the expression (4.6) for $(R_1^{(1)})_{2,2}$. This completes the proof of Proposition 4.1.

## Proof of Theorem 1.7 and of the Expression (1.10) for *c*

In this section, we use the expansions of *p* and *R* derived in Sects. 3 and 4 to prove Theorem 1.7 and to provide a first proof of the expression (1.10) for the constant *c*.

### Proof of Theorem 1.7

Propositions 3.2 and 4.1 yield expansions for $p_{1}(s)$ and $R_1(s)$ in negative powers of $s^\rho$ to all orders uniformly for $\theta$ in compact subsets of (0, 1]. Indeed, since *p* is analytic at $\zeta = \infty$, (2.20) implies

$$\begin{aligned} p_1(s) = \frac{1}{2\pi i} \int _{|\zeta |=r} p(\zeta ) d\zeta \end{aligned}$$

where *r* is any fixed large radius; substituting in (3.2), this gives the following extension of (2.37) to all orders as $s \rightarrow + \infty$:

4.36 $$\begin{aligned} p_1(s)= & {} -ic_{5}|b_{2}| + i \frac{c_{5}+c_{6}}{2}(|b_{2}|-\mathrm{Im}\,{b_{2}}) \nonumber \\&+ \sum _{n=1}^{N} \frac{\frac{1}{2\pi i} \int _{|\zeta |=r} \zeta ^{-n} {\mathcal {A}}_n(\zeta ) d\zeta }{s^{n\rho }} + {{{\mathcal {O}}}}\bigg ( \frac{1}{s^{(N+1)\rho }} \bigg ). \end{aligned}$$

Similarly, by the definition (2.33) of $R_1(s)$ and the expansion (4.1) of $R(\zeta )$,

$$\begin{aligned} R_1(s)= & {} \lim _{r \rightarrow +\infty } \frac{1}{2\pi i} \int _{|\zeta |=r} R(\zeta ) d\zeta \nonumber \\= & {} \lim _{r \rightarrow +\infty } \bigg \{\frac{1}{2\pi i} \int _{|\zeta |=r} \sum _{n=1}^{N} \frac{R^{(n)}(\zeta )}{s^{n\rho }}d\zeta + \int _{|\zeta |=r} {\mathfrak {g}}(s, \zeta ) d\zeta \bigg \}, \end{aligned}$$

where the function ${\mathfrak {g}}$ obeys the bound $|{\mathfrak {g}}(\zeta , s)| \le C' s^{-(N+1)\rho }(1+ |\zeta |)^{-1}$. The coefficients $R^{(n)}(\zeta )$ are analytic at $\zeta = \infty$ by Proposition 4.1. Hence

$$\begin{aligned} R_1(s) = \sum _{n=1}^{N} \frac{R_1^{(n)}}{s^{n\rho }} + {{{\mathcal {O}}}}(s^{-(N+1)\rho }), \qquad s \rightarrow +\infty , \end{aligned}$$

where $R_1^{(n)}$ denotes the coefficient of $\zeta ^{-1}$ in the large $\zeta$ expansion of $R^{(n)}(\zeta )$, and we have used that

$$\begin{aligned} \bigg |\lim _{r \rightarrow +\infty } \int _{|\zeta |=r} {\mathfrak {g}}(s, \zeta ) d\zeta \bigg | \le \limsup _{r \rightarrow +\infty } 2\pi r C' s^{-(N+1)\rho }(1+ r)^{-1} = 2\pi C' s^{-(N+1)\rho }. \end{aligned}$$

Since (2.35) expresses $\partial _{s} \ln \det \big ( 1- {\mathbb {K}}\big |_{[0,s]}\big )$ identically in terms of $p_{1}(s)$ and $(R_1(s))_{2,2}$, we deduce the existence of an asymptotic expansion to all orders of $\det (1- \left. {\mathbb {K}}\right| _{[0,s]})$ as $s \rightarrow +\infty$ for each $\theta \in (0,1]$. This proves Theorem 1.7.

### Proof of the expression (1.10) for *c*

Comparing (2.37) and (5.1), we see that

$$\begin{aligned} {\mathcal {K}} = \frac{1}{2\pi i} \int _{|\zeta |=r} \frac{{\mathcal {A}}_{1}(\zeta )}{\zeta } d\zeta , \end{aligned}$$

where $r > 0$ is any large radius, i.e., ${\mathcal {K}}$ is the term of order 1 in the large $\zeta$ expansion of the function ${\mathcal {A}}_{1}(\zeta )$ defined in (3.4). A direct computation shows that

$$\begin{aligned} {\mathcal {A}}_{1}(\zeta )=\frac{c_8- \frac{3\alpha ^2-1}{12}}{2|b_2|}\zeta + \frac{i}{2}\bigg \{c_8- \bigg (c_8- \frac{3\alpha ^2-1}{12}\bigg )\frac{\mathrm{Im}\,b_2 }{|b_2|}\bigg \} + O(\zeta ^{-1}), \qquad \zeta \rightarrow \infty , \end{aligned}$$

and therefore

5.1 $$\begin{aligned} {\mathcal {K}} = \frac{i}{2}\bigg \{ c_8- \bigg (c_8- \frac{3\alpha ^2-1}{12}\bigg )\frac{\mathrm{Im}\,b_2 }{|b_2|}\bigg \}. \end{aligned}$$

Substituting the expressions (4.6) and (5.2) for $(R_1^{(1)})_{2,2}$ and ${\mathcal {K}}$ into (2.38) and recalling the definition (2.4) of the constants $\{c_j\}_1^8$, we obtain the expression (1.10) for *c*.

#### Remark 5.1

The above evaluation of the constant *c* is based on the differential identity (2.35) in *s*. In Sect. 10, we will obtain an independent second proof of (1.10) by using a differential identity in $\theta$.

#### Remark 5.2

(The constant *c* for two other models). Our approach to obtain the constant *c* presented in Sects. 3 and 4 is based on the differential identity in *s* derived in [12]. Hence, it also applies to two other random matrix models studied in [12]. The first model consists of random matrices of the form

$$\begin{aligned} M^{(1)}= (G_r \ldots G_1)^*G_r\ldots G_1, \end{aligned}$$

where ${}^*$ denotes the complex conjugate transpose operator, and each $G_j$ is an independent $(n+\nu _j)\times (n+\nu _{j-1})$ complex Ginibre matrix, with integers $r\ge 1$, $\nu _0=0$, and $\nu _j\ge 0$, $j=1,\ldots ,r$. The second model consists of products of the form

$$\begin{aligned} M^{(2)}=(T_r\ldots T_1)^*T_r\ldots T_1, \end{aligned}$$

where each $T_{j}$ is an $(n+\nu _j)\times (n+\nu _{j-1})$ upper left truncation of an $\ell _j \times \ell _j$ Haar distributed unitary matrix $U_j$. Here $U_1,\ldots ,U_r$ are assumed to be independent and $\nu _0=0$, $r\ge 1$, and $\nu _j\ge 0$, $j=1,\ldots ,r$, are integers. Furthermore, it is assumed that $\ell _j \ge n+\nu _j+1$ and $\sum _{j=1}^{r}(\ell _j-n-\nu _j)\ge n$. In the second model, a subset $J\subset \{2,\ldots ,r\}$ of cardinality $q<r$ is fixed such that $\mu _j:=\ell _{k_j}-n>\nu _j$ for $k_j \in J$ and $\ell _k -n \rightarrow +\infty$ for $k\in \{ 1,\ldots r \} {\setminus } J$ as *n* and $\ell _1,\ldots ,\ell _r$ go to infinity. In [12], it is shown that these two models admit large gap asymptotics for the eigenvalues of the form

$$\begin{aligned} {\mathbb {P}}^{(j)}(\text{ gap } \text{ on } [0,s])&= C^{(j)} \exp \left( -a^{(j)} s^{2\rho ^{(j)}} + b^{(j)} s^{\rho } + c^{(j)} \ln s \right) (1 + o(1)) \qquad \\&\quad \text{ as } s \rightarrow + \infty , \end{aligned}$$

where the first and second model corresponds to $j=1$ and $j=2$, respectively. Moreover, explicit expressions are derived for the constants $\rho ^{(j)}$, $a^{(j)}$, and $b^{(j)}$.

A straightforward modification of our approach yields the existence of constants $C_1^{(j)},\ldots ,C_N^{(j)} \in {\mathbb {R}}$ such that

5.2 $$\begin{aligned}&{\mathbb {P}}^{(j)}(\text{ gap } \text{ on } [0,s]) \nonumber \\&\quad = C^{(j)} \exp \Big ( -a^{(j)} s^{2\rho ^{(j)}}+b^{(j)} s^{\rho }+c^{(j)} \ln s + \sum _{j=1}^{N}C_j^{(j)} s^{-j\rho } + {{{\mathcal {O}}}}\big (s^{-(N+1)\rho }\big ) \Big ), \end{aligned}$$

as $s\rightarrow +\infty$ for $j=1,2$, and shows that the constants $c^{(1)}$ and $c^{(2)}$ are given explicitly by

5.3 $$\begin{aligned} c^{(1)}&= \frac{r-1}{12(r+1)} - \frac{1}{2(r+1)} \sum _{j=1}^{r} \nu _j^2, \nonumber \\ c^{(2)}&= \frac{r-q-1}{12(r-q+1)}- \frac{1}{2(r-q+1)} \bigg ( \sum _{j=1}^r \nu _j^2 - \sum _{j=1}^{q} \mu _j^2 \bigg ). \end{aligned}$$

Let ${\mathbb {K}}^{(1)}$ be the hard edge limiting kernel for the eigenvalues associated to the first model presented above (this is the same notation as in [12]). For certain particular choices of the parameters $\nu _{1}, \ldots ,\nu _{r}$ and $\theta$, the kernel ${\mathbb {K}}^{(1)}$ defines the same point process (up to rescaling) as the one associated to ${\mathbb {K}}$^7^–this is a result of Kuijlaars and Stivigny, see [25, Theorem 5.1]. More precisely, if $r \ge 1$ is an integer, $\alpha > -1$ and

5.4 $$\begin{aligned}&\theta = \frac{1}{r},&\nu _{j} = \alpha + \frac{j-1}{r}, \qquad j = 1,\ldots ,r, \end{aligned}$$

then the kernels ${\mathbb {K}}^{(3)}$ and ${\mathbb {K}}$ are related by

$$\begin{aligned} \left( \frac{x}{y} \right) ^{\alpha }{\mathbb {K}}^{(1)}(x,y) = r^{r} {\mathbb {K}}^{(3)}(r^{r}x,r^{r}y). \end{aligned}$$

Therefore, if the parameters satisfy (5.5), we obtain the following relations:^8^

5.5 $$\begin{aligned}&\rho ^{(1)} = \rho , \qquad a^{(1)} = a r^{2r\rho }, \qquad b^{(1)} = b r^{r\rho }, \end{aligned}$$

5.6 $$\begin{aligned}&c^{(1)} = c, \qquad C^{(1)} = r^{rc}C. \end{aligned}$$

The three relations in (5.6) can be verified from [12], and the relation $c^{(1)} = c$ can be verified directly from (1.10) and (5.4). This provides a non-trivial consistency check of the results from [12] and of our result for *c* and $c^{(1)}$.

## Differential Identity in $\theta$

In this section, we derive an identity for the derivative of $\ln \det (1-{\mathbb {K}}|_{[0,s]})$ with respect to $\theta$. As explained in Sect. 1.2, this differential identity is needed for the derivation of the expression (1.11) for *C*. Our proof of Lemma 6.1 below is inspired by the derivation of the differential identity (2.35) given in [12].

### Lemma 6.1

(Differential identity in $\theta$, 1st version). For every $\alpha > -1$, $\theta > 0$ and $s>0$, the following identity holds:

5.7 $$\begin{aligned}&\partial _{\theta } \ln \det \Big ( \left. 1-{\mathbb {K}} \right| _{[0,s]} \Big ) = \frac{1}{2} \int _{\gamma \cup {\widetilde{\gamma }}} \partial _{\theta } \ln \Gamma \Big ( \frac{\frac{\alpha }{2}+1-z}{\theta } \Big ) {{\,\mathrm{Tr}\,}}[Y_{+}^{-1}(z)Y_{+}^{\prime }(z)\sigma _{3} \nonumber \\&\qquad \qquad \qquad \qquad \qquad \qquad \qquad - Y_{-}^{-1}(z)Y_{-}^{\prime }(z)\sigma _{3}] \frac{dz}{2\pi i} \nonumber \\&= \frac{-1}{2\theta ^{2}} \int _{\gamma \cup {\widetilde{\gamma }}} \Big (\frac{\alpha }{2}+1-z\Big ) \psi \Big ( \frac{\frac{\alpha }{2}+1-z}{\theta } \Big ) {{\,\mathrm{Tr}\,}}[Y_{+}^{-1}(z)Y_{+}^{\prime }(z)\sigma _{3} - Y_{-}^{-1}(z)Y_{-}^{\prime }(z)\sigma _{3}] \frac{dz}{2\pi i}. \end{aligned}$$

where $\psi = (\ln \Gamma )^{\prime }$ is the di-gamma function.

### Proof

From [4, Theorem 2.1] and [12, Eq. (2.21)], letting $\theta$ play the role of the deformation parameter, we deduce that

6.1 $$\begin{aligned} \partial _{\theta } \ln \det \Big ( \left. 1-{\mathbb {K}} \right| _{[0,s]} \Big ) = \int _{\gamma \cup {\widetilde{\gamma }}} {{\,\mathrm{Tr}\,}}[Y_{-}^{-1}(z)Y_{-}^{\prime }(z)\partial _{\theta }J(z)J^{-1}(z)] \frac{dz}{2\pi i}, \end{aligned}$$

where $$J(z) := Y_{-}^{-1}(z)Y_{+}(z)$$, i.e.,

$$\begin{aligned} J(z) = \left\{ \begin{array}{l l} \displaystyle \begin{pmatrix} 1 &{} -s^{-z}F(z) \\ 0 &{} 1 \end{pmatrix}, &{} z \in \gamma , \\ \displaystyle \begin{pmatrix} 1 &{} 0 \\ s^{z}F(z)^{-1} &{} 1 \end{pmatrix},&z \in {\tilde{\gamma }}. \end{array} \right. \end{aligned}$$

A computation gives

$$\begin{aligned} \partial _{\theta }J(z)J(z)^{-1} = \partial _{\theta } \ln \Gamma \Big ( \frac{\frac{\alpha }{2}+1-z}{\theta } \Big ) (J(z)-I)\sigma _{3}, \end{aligned}$$

from which it follows that

6.2 $$\begin{aligned} \partial _{\theta } \ln \det \Big ( \left. 1-{\mathbb {K}} \right| _{[0,s]} \Big ) = \int _{\gamma \cup {\widetilde{\gamma }}} \partial _{\theta } \ln \Gamma \Big ( \frac{\frac{\alpha }{2}+1-z}{\theta } \Big ) {{\,\mathrm{Tr}\,}}[Y_{-}^{-1}Y_{-}^{\prime }(J-I)\sigma _{3}] \frac{dz}{2\pi i}.\nonumber \\ \end{aligned}$$

Since *J* is triangular and $J-I$ is off-diagonal we infer that

$$\begin{aligned} J\sigma _{3}J^{-1} = (2J-I)\sigma _{3}, \end{aligned}$$

using also the jump relations for *Y*, from which we obtain

6.3 $$\begin{aligned} {{\,\mathrm{Tr}\,}}[Y_{+}^{-1}Y_{+}^{\prime }\sigma _{3}] = 2 {{\,\mathrm{Tr}\,}}[Y_{-}^{-1}Y_{-}^{\prime }J\sigma _{3}]-{{\,\mathrm{Tr}\,}}[Y_{-}^{-1}Y_{-}^{\prime }\sigma _{3}]. \end{aligned}$$

A similar computation yields

6.4 $$\begin{aligned} {{\,\mathrm{Tr}\,}}[JY_{+}^{-1}Y_{+}^{\prime }\sigma _{3}] = {{\,\mathrm{Tr}\,}}[Y_{-}^{-1}Y_{-}^{\prime }J\sigma _{3}]. \end{aligned}$$

By substituting (6.5) in (6.4), we obtain

6.5 $$\begin{aligned} {{\,\mathrm{Tr}\,}}[(I-J)Y_{+}^{-1}Y_{+}^{\prime }\sigma _{3}] = {{\,\mathrm{Tr}\,}}[Y_{-}^{-1}Y_{-}^{\prime }(J-I)\sigma _{3}]. \end{aligned}$$

Using (6.5) and (6.6), we arrive at

$$\begin{aligned} {{\,\mathrm{Tr}\,}}[Y_{-}^{-1}Y_{-}^{\prime }(J-I)\sigma _{3}]&= \displaystyle \frac{1}{2} \Big ( {{\,\mathrm{Tr}\,}}[(I-J)Y_{+}^{-1}Y_{+}^{\prime }\sigma _{3}] + {{\,\mathrm{Tr}\,}}[Y_{-}^{-1}Y_{-}^{\prime }(J-I)\sigma _{3}] \Big )\\&= \displaystyle \frac{1}{2} \Big ( {{\,\mathrm{Tr}\,}}[Y_{+}^{-1}Y_{+}^{\prime }\sigma _{3}] - {{\,\mathrm{Tr}\,}}[Y_{-}^{-1}Y_{-}^{\prime }\sigma _{3}] \Big ). \end{aligned}$$

Substitution of the above identity into (6.3) finishes the proof. $\quad \square$

In the following lemma, we rewrite the differential identity (6.1) in a form which is more convenient for the asymptotic analysis. Let us define the sequence $\{\zeta _j\}_0^\infty \subset i{\mathbb {R}}$ by

6.6 $$\begin{aligned} \zeta _j = -i\frac{\frac{1 +\alpha }{2} + j\theta }{s^\rho }, \qquad j = 0,1,2, \dots , \end{aligned}$$

and the meromorphic function $H(\zeta )$ by

6.7 $$\begin{aligned} H(\zeta ) = \frac{1}{\theta ^{2}} \left( \frac{1+\alpha }{2}- i s^{\rho }\zeta \right) \psi \bigg (\frac{\frac{1+\alpha }{2}-is^{\rho }\zeta }{\theta } \bigg ). \end{aligned}$$

Note that *H* has a simple pole at each of the points $\zeta _j$, $j = 1,2, \dots$, and no other poles in ${\mathbb {C}}$; the point $\zeta _0$ is a simple pole of $\psi (\frac{\frac{1+\alpha }{2}-is^{\rho }\zeta }{\theta })$ but not of *H*.

**Fig. 8:**
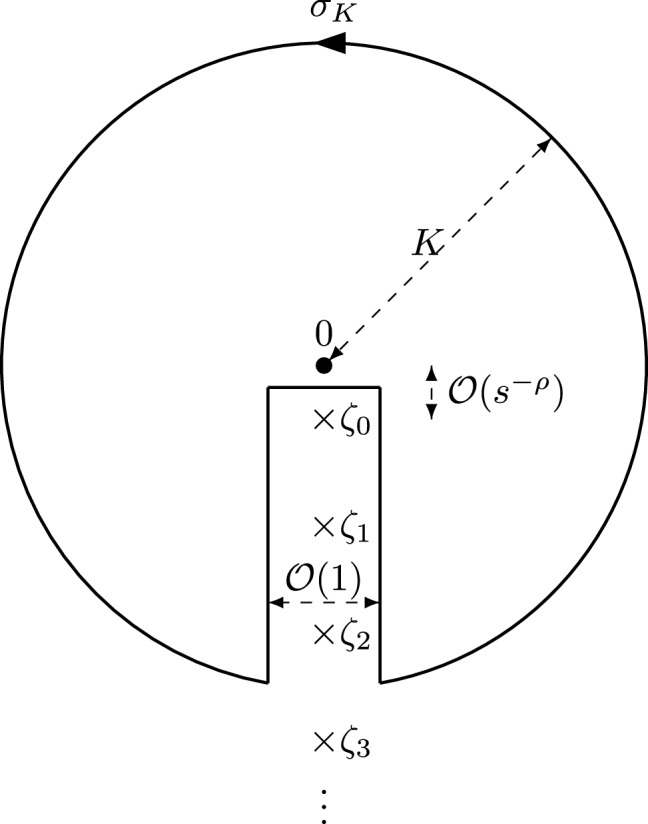
The contour $\sigma _{K}$ and the poles $\{\zeta _{j}\}_0^\infty$ of $\psi (\frac{\frac{1+\alpha }{2}-is^{\rho }\zeta }{\theta })$ in the complex $\zeta$-plane. The uppermost pole $\zeta _0$ lies a distance ${{{\mathcal {O}}}}(s^{-\rho })$ from the origin as $s \rightarrow + \infty$. The horizontal line segment has a length of order ${{{\mathcal {O}}}}(1)$ as $s \rightarrow +\infty$ and crosses the imaginary axis half-way between the origin and $\zeta _0$

Given $K > |b_1|$, we let $\sigma _K$ denote the closed *s*-dependent counterclockwise contour displayed in Fig. 8. The contour $\sigma _{K}$ surrounds $\Sigma _{5}$ once in the positive direction, but does not surround any of the poles $\zeta _{j}$ of *H*. The circular part of $\sigma _{K}$ has radius *K* and its horizontal part has a length of order ${{{\mathcal {O}}}}(1)$ as $s \rightarrow +\infty$ and crosses the imaginary axis at the point $\zeta _0/2$. If $K = 2|b_1|$, we write $\sigma$ for $\sigma _K$, i.e., $\sigma = \sigma _{2|b_1|}$. We also define the contour ${\widetilde{\Sigma }}_{K}$ as the union of the parts exterior to $\sigma _K$ of the rays $\{\Sigma _{i}\}_1^4$ defined in (2.14), i.e.,

$$\begin{aligned} {\widetilde{\Sigma }}_{K} = \bigcup _{i=1}^{4} \Sigma _{i} {\setminus } \{|\zeta | \le K\}. \end{aligned}$$

### Lemma 6.2

(Differential identity in $\theta$, 2nd version). Let *K* be such that $K > 2|b_{1}|$. Then

6.8 $$\begin{aligned}&\partial _{\theta } \ln \det \Big ( \left. 1-{\mathbb {K}} \right| _{[0,s]} \Big ) = I_{1} + I_{2} + I_{3,K} + I_{4,K}, \end{aligned}$$

where

6.9 $$\begin{aligned} I_{1}&= s^{\rho } \int _{\sigma } H(\zeta ) g'(\zeta ) \frac{d\zeta }{2\pi i}, \end{aligned}$$

6.10 $$\begin{aligned} I_{2}&= \frac{1}{2} \int _{\sigma } H(\zeta ) {{\,\mathrm{Tr}\,}}\Big [ P^{\infty }(\zeta )^{-1}P^{\infty }(\zeta )' \sigma _{3} \Big ] \frac{d\zeta }{2\pi i}, \end{aligned}$$

6.11 $$\begin{aligned} I_{3,K}&= \frac{1}{2} \int _{\sigma _{K}} H(\zeta ) {{\,\mathrm{Tr}\,}}\Big [P^{\infty }(\zeta )^{-1}e^{-p_{0}\sigma _{3}}R^{-1}(\zeta )R'(\zeta )e^{p_{0}\sigma _{3}}P^{\infty }(\zeta )\sigma _{3}\Big ] \frac{d\zeta }{2\pi i}, \end{aligned}$$

6.12 $$\begin{aligned} I_{4,K}&= -\frac{1}{2} \int _{{\widetilde{\Sigma }}_{K}} H(\zeta ) {{\,\mathrm{Tr}\,}}\Big [P^{\infty }(\zeta )^{-1}e^{-p_{0}\sigma _{3}}\Big (R_{+}^{-1}(\zeta )R_{+}'(\zeta ) \nonumber \\&\quad -R_{-}^{-1}(\zeta )R_{-}'(\zeta )\Big )e^{p_{0}\sigma _{3}}P^{\infty }(\zeta )\sigma _{3}\Big ] \frac{d\zeta }{2\pi i}. \end{aligned}$$

### Proof

Using the change of variable $z = is^{\rho } \zeta + \frac{1}{2}$ in (6.1), we obtain an integral over $\gamma _{U}\cup {\tilde{\gamma }}_{U}$ whose integrand is expressed in terms of *U* via (2.13). By deforming the contour of this integral using the analytic continuations of $U_{+}$ and $U_{-}$ (i.e., using *T*), we arrive at

$$\begin{aligned}&\partial _{\theta } \ln \det \Big ( \left. 1-{\mathbb {K}} \right| _{[0,s]} \Big ) \\&\quad = -\frac{1}{2} \int _{\cup _{i=1}^{5}\Sigma _{i}} H(\zeta ) {{\,\mathrm{Tr}\,}}[T_{+}^{-1}(\zeta )T_{+}^{\prime }(\zeta )\sigma _{3} - T_{-}^{-1}(\zeta )T_{-}^{\prime }(\zeta )\sigma _{3}] \frac{d\zeta }{2\pi i}. \end{aligned}$$

Another contour deformation gives

6.13 $$\begin{aligned} \partial _{\theta } \ln \det \Big ( \left. 1-{\mathbb {K}} \right| _{[0,s]} \Big ) =&\; \frac{1}{2} \int _{\sigma _{K}} H(\zeta ) {{\,\mathrm{Tr}\,}}[T^{-1}(\zeta )T^{\prime }(\zeta )\sigma _{3}] \frac{d\zeta }{2\pi i}\nonumber \\&-\frac{1}{2} \int _{{\widetilde{\Sigma }}_{K} } H(\zeta ) {{\,\mathrm{Tr}\,}}[T_{+}^{-1}(\zeta )T_{+}^{\prime }(\zeta )\sigma _{3} - T_{-}^{-1}(\zeta )T_{-}^{\prime }(\zeta )\sigma _{3}] \frac{d\zeta }{2\pi i}, \end{aligned}$$

where we have used that $\zeta \mapsto H(\zeta ) {{\,\mathrm{Tr}\,}}[T^{-1}(\zeta )T^{\prime }(\zeta )\sigma _{3}]$ is analytic for all $\zeta$ in the interior region of $\sigma _{K}$ such that $\zeta \notin \cup _{i=1}^{5}\Sigma _{i}$.

For $\zeta \in \sigma _{K}$, we have $\zeta \notin {\mathbb {D}}_\delta (b_1)\cup {\mathbb {D}}_\delta (b_2)$. Therefore, inverting the transformations $T \mapsto S \mapsto R$ for $\zeta \in \sigma _{K}$, we find

6.14 $$\begin{aligned} {{\,\mathrm{Tr}\,}}[T^{-1}(\zeta )T^{\prime }(\zeta )\sigma _{3}] =&\; 2s^{\rho } g'(\zeta ) + {{\,\mathrm{Tr}\,}}[P^{\infty }(\zeta )^{-1}P^{\infty }(\zeta )'\sigma _{3}] \nonumber \\&+ {{\,\mathrm{Tr}\,}}[P^{\infty }(\zeta )^{-1}e^{-p_{0}\sigma _{3}}R^{-1}(\zeta )R'(\zeta )e^{p_{0}\sigma _{3}}P^{\infty }(\zeta )\sigma _{3}]. \end{aligned}$$

The first two terms on the right-hand side of (6.15) are analytic in the region between $\sigma$ and $\sigma _{K}$. Therefore, substituting (6.15) into the first term on the right-hand side of (6.14) and deforming the contour from $\sigma _K$ to $\sigma$ in the integrals involving the first two terms on the right-hand side of (6.15), we find that this term equals $I_{1} + I_{2} + I_{3,K}$.

Similarly, by inverting the transformations $T \mapsto S \mapsto R$ for $\zeta \in {\widetilde{\Sigma }}_{K}$, we find that the second term on the right-hand side of (6.14) equals $I_{4,K}$. $\quad \square$

### Remark 6.3

In the application of the differential identity (6.9) to the proof of Theorem 1.1, we will choose $K = s^\rho$; that is, the radius *K* will be *s*-dependent and growing to infinity as $s \rightarrow +\infty$.

The remainder of the paper is devoted to the proof of Theorem 1.1. The proof is divided into two steps. The first step consists of obtaining large *s* asymptotics of the differential identity (6.9) uniformly for $\theta$ in compact subsets of (0, 1]. This is achieved by computing the large *s* asymptotics of each of the four terms $I_1$, $I_2$, $I_{3,K}$, and $I_{4,K}$ on the right-hand side of (6.9). These computations are presented in Sects. 7–9. The second step is presented in Sect. 10 and consists of integrating the resulting asymptotic expansion from $\theta = 1$ to an arbitrary $\theta \in (0,1]$.

## Asymptotics of $I_{1}$

In this section, we prove the following proposition which establishes the large *s* asymptotics of $I_{1}$.

### Proposition 7.1

(Large *s* asymptotics of $I_{1}$). Let $\alpha > -1$. As $s \rightarrow +\infty$, the function $I_1$ defined in (6.10) satisfies

6.15 $$\begin{aligned} I_{1}&= {\mathcal {I}}_{1}^{(1)} s^{2\rho } \ln (s^\rho ) + I_{1}^{(1)} s^{2\rho } + {\mathcal {I}}_{1}^{(2)} s^{\rho } \ln (s^\rho )\nonumber \\&\quad + I_{1}^{(2)} s^{\rho } + {\mathcal {I}}_{1}^{(3)}\ln (s^\rho ) + I_{1}^{(3)} + {{{\mathcal {O}}}}(s^{-\rho }\ln (s^{\rho })) \end{aligned}$$

uniformly for $\theta$ in compact subsets of (0, 1], where the coefficients ${\mathcal {I}}_{1}^{(1)}$, $I_{1}^{(1)}$, ${\mathcal {I}}_{1}^{(2)}$, $I_{1}^{(2)}$, ${\mathcal {I}}_{1}^{(3)}$, $I_{1}^{(3)}$ are given by

7.1 $$\begin{aligned} {\mathcal {I}}_{1}^{(1)} =&- \frac{2a}{\rho (1+\theta )^2}, \end{aligned}$$

7.2a $$\begin{aligned} I_1^{(1)} =&- \partial _\theta a, \end{aligned}$$

7.2b $$\begin{aligned} {\mathcal {I}}_{1}^{(2)} =&\; 0, \end{aligned}$$

7.2c $$\begin{aligned} I_{1}^{(2)} =&-\frac{(1+\theta )(1+\alpha )}{2\theta }\theta ^{-\frac{2\theta }{1+\theta }}, \end{aligned}$$

7.2d $$\begin{aligned} {\mathcal {I}}_{1}^{(3)} =&\; \frac{3(1+\alpha )^{2}-2\theta ^{2}}{24\theta ^{2}}, \end{aligned}$$

7.2e $$\begin{aligned} I_{1}^{(3)} =&\; \frac{1+\alpha }{4\theta } \ln (2\pi ) + \frac{3(1+\alpha )^{2}-2\theta ^{2}}{24\theta ^{2}} \left( - \frac{2\theta }{\theta +1}\ln \theta + \frac{\theta -1}{\theta } \ln (1+\theta ) \right) \nonumber \\&+ \zeta ^{\prime }(-1) - \ln G \Big ( \frac{1+\alpha +2\theta }{2\theta } \Big ). \end{aligned}$$

### Proof

Recall from (6.7) that $\zeta _0 = -i\frac{1 +\alpha }{2}s^{-\rho }$. Define $\Psi (\zeta ) = \Psi (\zeta , s, \theta , \alpha )$ by

7.2f $$\begin{aligned} \Psi (\zeta ) = s^{\rho } \int _{\zeta _{\star }}^\zeta H(\xi ) d\xi , \end{aligned}$$

where $\zeta _{\star } \in {\mathbb {C}}{\setminus } (-i\infty , \zeta _0]$ is some point at which $\Psi$ is normalized to vanish; we will choose this normalization point below. Then $\Psi$ is analytic in ${\mathbb {C}}{\setminus } (-i\infty , \zeta _0]$. In particular, $\Psi$ is analytic on $\sigma$. Using the explicit expression (2.10) for $g''$, an integration by parts therefore gives

$$\begin{aligned} I_{1}&= - \int _{\sigma } \Psi (\zeta ) g''(\zeta ) \frac{d\zeta }{2\pi i} \\&= i\frac{c_1 + c_2}{2}\int _{\sigma } \Psi (\zeta ) \bigg (\frac{1}{\zeta } - \frac{1}{r(\zeta )} + \frac{i \mathrm{Im}\,{b_1}}{\zeta r(\zeta )}\bigg ) \frac{d\zeta }{2\pi i} \\&= i\frac{c_1 + c_2}{2}\Psi (0) + i\frac{c_1 + c_2}{2}\int _{\sigma } \Psi (\zeta ) \bigg (- \frac{1}{r(\zeta )} + \frac{i \mathrm{Im}\,{b_1}}{\zeta r(\zeta )}\bigg ) \frac{d\zeta }{2\pi i}. \end{aligned}$$

We assume that $\sigma$ is big enough to enclose the straight line segment $[b_1, b_2]$ and move the branch cut for $r(\zeta )$ upwards from $\Sigma _5$ to the horizontal line segment $[b_1, b_2]$; this does not change the value of the integral. We let ${\tilde{r}}$ denote the analytic continuation of *r* defined by

7.3 $$\begin{aligned} {\tilde{r}}(\zeta ) = [(\zeta -b_{1})(\zeta -b_{2})]^{\frac{1}{2}}, \end{aligned}$$

where the branch is such that ${\tilde{r}}$ is analytic in ${\mathbb {C}}{\setminus } [b_{1},b_{2}]$ and ${\tilde{r}}(\zeta ) \sim \zeta$ as $\zeta \rightarrow \infty$. Then ${\tilde{r}}(\zeta )$ is equal to $r(\zeta )$ except for $\zeta$ in the region enclosed by $\Sigma _5 \cup [b_{1},b_{2}]$ where we instead have ${\tilde{r}}(\zeta ) = -r(\zeta )$. Deforming $\sigma$ upwards through the origin, a residue contribution is generated by the simple pole of $i \mathrm{Im}\,{b_1}/(\zeta {\tilde{r}}(\zeta ))$ at $\zeta = 0$. We find

$$\begin{aligned} I_{1} =&\; i\frac{c_1 + c_2}{2}\Psi (0) + i\frac{c_1 + c_2}{2}\bigg \{\Psi (0) \frac{i \mathrm{Im}\,{b_1}}{{\tilde{r}}(0)} \\&+ \int _{[b_1, b_2]} \Psi (\zeta ) \bigg [\bigg (- \frac{1}{{\tilde{r}}(\zeta )} + \frac{i \mathrm{Im}\,{b_1}}{\zeta {\tilde{r}}(\zeta )}\bigg )_- - \bigg (- \frac{1}{{\tilde{r}}(\zeta )} + \frac{i \mathrm{Im}\,{b_1}}{\zeta {\tilde{r}}(\zeta )}\bigg )_+ \bigg ] \frac{d\zeta }{2\pi i} \bigg \} \end{aligned}$$

where $[b_1, b_2]$ is oriented from $b_1$ to $b_2$ with $+$ and − sides to the left and right as usual, and ${\tilde{r}}(0) = r_-(0) = -i|b_2|$. Thus,

7.4 $$\begin{aligned} I_{1} =&\; i\frac{c_1 + c_2}{2}\Psi (0)\bigg (1 - \frac{\mathrm{Im}\,{b_1}}{|b_1|}\bigg ) -2i\frac{c_1 + c_2}{2} \int _{[b_1, b_2]} \Psi (\zeta ) \bigg (- \frac{1}{{\tilde{r}}_+(\zeta )} + \frac{i \mathrm{Im}\,{b_1}}{\zeta {\tilde{r}}_+(\zeta )}\bigg ) \frac{d\zeta }{2\pi i} \nonumber \\ =&\; i\frac{c_1 + c_2}{2}\Psi (0)\bigg (1 - \frac{\mathrm{Im}\,{b_1}}{|b_1|}\bigg ) - 2i\frac{c_1 + c_2}{2} \int _{\gamma _{b_2b_1}} \Psi (\zeta ) \bigg (1 - \frac{i \mathrm{Im}\,{b_1}}{\zeta }\bigg )\frac{1}{r(\zeta )} \frac{d\zeta }{2\pi i}, \end{aligned}$$

where $\gamma _{b_2b_1}$ denotes the part of the circle of radius $|b_2|$ centered at the origin going from $b_2$ to $b_1$ and oriented counterclockwise.

Let us choose $\zeta _{\star } = 0$; then $\Psi (0) = 0$, so the first term on the right-hand side of (7.5) vanishes. The choice $\zeta _{\star } = 0$ implies that the term $\frac{\frac{1+\alpha }{2}-is^{\rho }\xi }{ \theta }$ is not uniformly large for $\xi \in [0,\zeta ]$ with $\zeta \in \gamma _{b_{1}b_{2}}$ as $s \rightarrow + \infty$, so the large *s* behavior of $\Psi (\zeta )$ does not follow immediately from (6.8) and (7.3); however, we can determine the large *s* asymptotics of $\Psi (\zeta )$ as follows. Using the change of variables

7.5 $$\begin{aligned} x = \frac{1}{\theta }\left( \frac{1+\alpha }{2}-i s^{\rho }\xi \right) , \qquad dx = \frac{-is^{\rho }}{\theta }d\xi , \end{aligned}$$

we can write

$$\begin{aligned} \displaystyle \Psi (\zeta ) =&\; i \int _{0}^{\zeta } \frac{1}{\theta }\left( \frac{1+\alpha }{2}-i s^{\rho }\xi \right) \psi \left( \frac{\frac{1+\alpha }{2}-is^{\rho }\xi }{\theta } \right) \frac{-is^{\rho }}{\theta }d\xi \\ =&\; i \int _{z_{\star }}^{z} x \psi (x)dx = i \int _{z_{\star }}^{z} x \partial _{x}\ln \Gamma (x)dx, \end{aligned}$$

where

$$\begin{aligned} z = \frac{1}{\theta }\left( \frac{1+\alpha }{2}-is^{\rho }\zeta \right) , \qquad z_{\star } = \frac{1+\alpha }{2\theta }. \end{aligned}$$

Integrating by parts, we get

7.6 $$\begin{aligned} \Psi (\zeta ) = i \left( \Big [x \ln \Gamma (x)\Big ]_{z_{\star }}^{z} - \int _{z_{\star }}^{z} \ln \Gamma (x)dx \right) . \end{aligned}$$

Using the well-known identity (see e.g. [27, Eq. 5.17.4])

7.7 $$\begin{aligned} \int _{1}^{z} \ln \Gamma (x)dx = \frac{z-1}{2}\ln (2\pi ) - \frac{(z-1)z}{2}+(z-1)\ln \Gamma (z) - \ln G(z) \end{aligned}$$

in (7.7), we obtain

7.8 $$\begin{aligned} \Psi (\zeta )= & {} i \left( \frac{is^{\rho }\zeta }{2\theta }\Big [ \ln (2\pi )+1 \Big ] - \frac{i s^{\rho }\zeta }{2\theta } \left( \frac{1+\alpha }{\theta }-\frac{is^{\rho }\zeta }{\theta } \right) + \ln \frac{\Gamma \left( \frac{1+\alpha }{2\theta }-\frac{is^{\rho }\zeta }{\theta } \right) }{\Gamma \left( \frac{1+\alpha }{2\theta } \right) }\right. \nonumber \\&\left. + \ln \frac{G \left( \frac{1+\alpha }{2\theta }-\frac{is^{\rho }\zeta }{\theta } \right) }{G\left( \frac{1+\alpha }{2\theta } \right) } \right) . \end{aligned}$$

The above expression is convenient since the large *z* asymptotics of $\Gamma (z)$ and *G*(*z*) are known (see e.g. [27, Eqs. 5.11.1 and 5.17.5]):

7.9 $$\begin{aligned}&\ln G(z+1) = \frac{z^{2}}{4}+z \ln \Gamma (z+1)-\left( \frac{z(z+1)}{2}+\frac{1}{12}\right) \ln z - \frac{1}{12} + \zeta ^{\prime }(-1) + {{{\mathcal {O}}}}(z^{-2}), \end{aligned}$$

7.10 $$\begin{aligned}&\ln \Gamma (z) = (z-\tfrac{1}{2})\ln z - z + \tfrac{1}{2}\ln (2\pi ) + \frac{1}{12z} + {{{\mathcal {O}}}}(z^{-3}), \end{aligned}$$

as $z \rightarrow \infty$ with $|\arg z|< \pi$, where $\zeta$ is Riemann’s zeta function.^9^ Expanding (7.9) as $s^{\rho }\zeta \rightarrow \infty$, we get

7.11 $$\begin{aligned} \displaystyle \Psi (\zeta )=&-\frac{i \zeta ^{2}}{2\theta ^{2}}s^{2\rho }\ln (s^{\rho }) + \frac{i \zeta ^{2}}{4\theta ^{2}}\Big ( 1-2 \ln \Big ( \frac{-i \zeta }{\theta } \Big ) \Big )s^{2\rho } + \frac{1+\alpha }{2\theta ^{2}}\zeta s^{\rho }\ln (s^{\rho }) \nonumber \\&+ \frac{1}{2\theta ^{2}}\Big ( -\zeta \theta + (1+\alpha )\zeta \ln \Big ( \frac{-i \zeta }{\theta } \Big ) \Big ) s^{\rho } + i \frac{3(1+\alpha )^{2}-2\theta ^{2}}{24\theta ^{2}} \ln (s^{\rho }) \nonumber \\&+ \frac{i}{24 \theta ^{2}}\Big (6(1+\alpha )\theta \ln (2\pi ) + (3(1+\alpha )^{2}-2\theta ^{2})\ln \Big ( \frac{-i \zeta }{\theta } \Big )\Big )\nonumber \\&+ i \Big ( \zeta ^{\prime }(-1) - \ln \Gamma \Big ( \frac{1+\alpha }{2\theta } \Big )- \ln G \Big ( \frac{1+\alpha }{2\theta } \Big ) \Big ) + {{{\mathcal {O}}}}\Big ( \frac{1}{\zeta s^{\rho }} \Big ). \end{aligned}$$

Substituting (7.12) into (7.5), we find that $I_1$ satisfies (7.1) as $s \rightarrow +\infty$ with coefficients given by

7.12 $$\begin{aligned} {\mathcal {I}}_{1}^{(1)}&= - 2i\frac{c_1 + c_2}{2} \int _{\gamma _{b_2b_1}} -\frac{i\zeta ^2}{2 \theta ^2} \bigg (1 - \frac{i \mathrm{Im}\,{b_1}}{\zeta }\bigg )\frac{1}{r(\zeta )} \frac{d\zeta }{2\pi i} \nonumber \\&= - \frac{c_1 + c_2}{2 \theta ^2} \bigg (\int _{\gamma _{b_2b_1}} \frac{\zeta ^2}{r(\zeta )} \frac{d\zeta }{2\pi i} - i \mathrm{Im}\,{b_1} \int _{\gamma _{b_2b_1}} \frac{\zeta }{r(\zeta )} \frac{d\zeta }{2\pi i} \bigg ), \nonumber \\ I_{1}^{(1)}&= - 2i\frac{c_1 + c_2}{2} \int _{\gamma _{b_2b_1}} \frac{i \zeta ^2 (1- 2 \ln (-\frac{i}{\theta }) - 2\ln {\zeta })}{4 \theta ^2} \bigg (1 - \frac{i \mathrm{Im}\,{b_1}}{\zeta }\bigg )\frac{1}{r(\zeta )} \frac{d\zeta }{2\pi i} \nonumber \\&= -\frac{1- 2 \ln (-\frac{i}{\theta })}{2} {\mathcal {I}}_{1}^{(1)} - \frac{c_1 + c_2}{2 \theta ^2} \int _{\gamma _{b_2b_1}} \zeta ^2 \ln (\zeta ) \bigg (1 - \frac{i \mathrm{Im}\,{b_1}}{\zeta }\bigg )\frac{1}{r(\zeta )} \frac{d\zeta }{2\pi i} \nonumber \\&= -\frac{1- 2 \ln (-\frac{i}{\theta })}{2} {\mathcal {I}}_{1}^{(1)} - \frac{c_1 + c_2}{2 \theta ^2} \bigg \{\int _{\gamma _{b_2b_1}} \frac{\zeta ^2 \ln (\zeta )}{r(\zeta )} \frac{d\zeta }{2\pi i} - i \mathrm{Im}\,{b_1} \int _{\gamma _{b_2b_1}} \frac{\zeta \ln (\zeta )}{r(\zeta )} \frac{d\zeta }{2\pi i}\bigg \}, \nonumber \\ {\mathcal {I}}_{1}^{(2)}&= - 2i\frac{c_1 + c_2}{2} \int _{\gamma _{b_2b_1}} \frac{(\alpha +1) \zeta }{2 \theta ^2} \bigg (1 - \frac{i \mathrm{Im}\,{b_1}}{\zeta }\bigg )\frac{1}{r(\zeta )} \frac{d\zeta }{2\pi i} \nonumber \\&= - 2i\frac{c_1 + c_2}{2} \frac{\alpha +1}{2 \theta ^2} \bigg \{\int _{\gamma _{b_2b_1}} \frac{\zeta }{r(\zeta )} \frac{d\zeta }{2\pi i} - i \mathrm{Im}\,{b_1} \int _{\gamma _{b_2b_1}} \frac{1}{r(\zeta )} \frac{d\zeta }{2\pi i} \bigg \}, \nonumber \\ I_{1}^{(2)}&= - 2i\frac{c_1 + c_2}{2} \int _{\gamma _{b_2b_1}} \frac{\zeta \left( -\theta +(\alpha +1) \ln (-\frac{i}{\theta }) + (\alpha +1) \ln {\zeta }\right) }{2 \theta ^2} \bigg (1 - \frac{i \mathrm{Im}\,{b_1}}{\zeta }\bigg )\frac{1}{r(\zeta )} \frac{d\zeta }{2\pi i} \nonumber \\&= \frac{-\theta +(\alpha +1) \ln (-\frac{i}{\theta })}{\alpha +1} {\mathcal {I}}_{1}^{(2)} \nonumber \\&\quad - 2i\frac{c_1 + c_2}{2} \frac{\alpha +1}{2 \theta ^2} \bigg \{\int _{\gamma _{b_2b_1}} \frac{\zeta \ln {\zeta }}{r(\zeta )} \frac{d\zeta }{2\pi i} - i \mathrm{Im}\,{b_1} \int _{\gamma _{b_2b_1}} \frac{\ln {\zeta }}{r(\zeta )}\frac{d\zeta }{2\pi i} \bigg \}, \nonumber \\ {\mathcal {I}}_{1}^{(3)}&= - 2i\frac{c_1 + c_2}{2} \int _{\gamma _{b_2b_1}} \frac{i \left( 3 (1+\alpha )^2 -2 \theta ^2 \right) }{24 \theta ^2} \bigg (1 - \frac{i \mathrm{Im}\,{b_1}}{\zeta }\bigg )\frac{1}{r(\zeta )} \frac{d\zeta }{2\pi i} \nonumber \\&= (c_1 + c_2) \frac{3 (1+\alpha )^2-2 \theta ^2}{24 \theta ^2} \bigg \{\int _{\gamma _{b_2b_1}} \frac{1}{r(\zeta )} \frac{d\zeta }{2\pi i} - i \mathrm{Im}\,{b_1} \int _{\gamma _{b_2b_1}} \frac{1}{\zeta r(\zeta )} \frac{d\zeta }{2\pi i}\bigg \}, \nonumber \\ I_{1}^{(3)} =&\; (c_{1}+c_{2}) \bigg \{ \frac{1}{24\theta ^{2}}\Big ( 6(1+\alpha )\theta \ln (2\pi ) + (3(1+\alpha )^{2}-2\theta ^{2})\ln (-\tfrac{i}{\theta }) \Big ) \nonumber \\&+\zeta ^{\prime }(-1)-\ln \Gamma \Big ( \frac{1+\alpha }{2\theta } \Big ) - \ln G \Big ( \frac{1+\alpha }{2\theta } \Big ) \bigg \} \int _{\gamma _{b_{2}b_{1}}} \left( 1 - i \frac{\mathrm{Im}\,b_{2}}{\zeta }\right) \frac{1}{r(\zeta )}\frac{d\zeta }{2\pi i} \nonumber \\&+(c_{1}+c_{2}) \frac{3(1+\alpha )^{2}-2\theta ^{2}}{24\theta ^{2}}\int _{\gamma _{b_{2}b_{1}}}\left( 1 - i \frac{\mathrm{Im}\,b_{2}}{\zeta } \right) \frac{\ln \zeta }{r(\zeta )} \frac{d\zeta }{2\pi i}. \end{aligned}$$

It only remains to show that the coefficients in (7.13) can be expressed as in (7.2). This requires the evaluation of several integrals; we have collected the necessary results in the next lemma.

### Lemma 7.2

Let $\alpha > -1$ and $\theta \in (0,1]$. Let $r(\zeta )$ denote the square root defined in (2.9). Then the following identities hold:

7.13 $$\begin{aligned} 2\int _{\gamma _{b_2b_1}} \frac{1}{r(\zeta )} \frac{d\zeta }{2\pi i} =&\; 1, \end{aligned}$$

7.14a $$\begin{aligned} 2\int _{\gamma _{b_2b_1}} \frac{\zeta }{r(\zeta )} \frac{d\zeta }{2\pi i} =&\; \frac{b_1 + b_2}{2}, \end{aligned}$$

7.14b $$\begin{aligned} 2\int _{\gamma _{b_2b_1}} \frac{\zeta ^2}{r(\zeta )} \frac{d\zeta }{2\pi i} =&\; \frac{3 b_1^2+2 b_1 b_2+3 b_2^2}{8}, \end{aligned}$$

7.14c $$\begin{aligned} 2 \int _{\gamma _{b_{2}b_{1}}} \frac{1}{\zeta r(\zeta )}\frac{d\zeta }{2\pi i} =&- \frac{i}{|b_{2}|}, \end{aligned}$$

7.14d $$\begin{aligned} 2\int _{\gamma _{b_2b_1}} \frac{\ln {\zeta }}{r(\zeta )} \frac{d\zeta }{2\pi i} =&\; \ln (i(|b_2| + \mathrm{Im}\,{b_2})) - \ln {2}, \end{aligned}$$

7.14e $$\begin{aligned} 2\int _{\gamma _{b_2b_1}} \frac{\zeta \ln {\zeta }}{r(\zeta )} \frac{d\zeta }{2\pi i} =&-i |b_2| + i\Big (1 + \ln (2i(|b_2| + \mathrm{Im}\,b_2)) - \ln 4\Big )\mathrm{Im}\,{b_2}, \end{aligned}$$

7.14f $$\begin{aligned} 2\int _{\gamma _{b_2b_1}} \frac{\zeta ^2 \ln {\zeta }}{r(\zeta )} \frac{d\zeta }{2\pi i} =&\; \frac{1}{4} \bigg (2 \left( (\mathrm{Re}\,{b_1})^2-2 (\mathrm{Im}\,{b_2})^2\right) \ln \big (2 i (|b_2|+\mathrm{Im}\,{b_2})\big )+6 |b_2| \mathrm{Im}\,(b_2) \nonumber \\&+(\mathrm{Im}\,{b_2})^2 (8\ln (2)-6)+(\mathrm{Re}\,{b_1})^2 (1-4 \ln {2})\bigg ), \end{aligned}$$

7.14g $$\begin{aligned} 2\int _{\gamma _{b_2b_1}} \frac{\ln {\zeta }}{\zeta r(\zeta )} \frac{d\zeta }{2\pi i} =&\; \frac{\ln (\frac{2i|b_2|^2}{|b_2| + \mathrm{Im}\,{b_2}})}{i|b_2|}. \end{aligned}$$

### Proof

See “Appendix C”. $$\quad \square $$

Since $\theta \in (0,1]$, we have $\arg b_{2} = \pi - \arg b_{1} \in [0, \pi /2)$, and hence

7.14h $$\begin{aligned}&\ln (ib_{1}) = \ln (b_{1}) - \frac{3\pi i}{2},&\ln (-ib_{1}) = \ln (b_{1}) - \frac{i \pi }{2}, \end{aligned}$$

7.15a $$\begin{aligned}&\ln (ib_{2}) = \ln (b_{2}) + \frac{\pi i}{2},&\ln (-ib_{2}) = \ln (b_{2}) - \frac{i \pi }{2}. \end{aligned}$$

Substituting the expressions of Lemma 7.2 into (7.13) and using (1.9), (2.4), (2.8), and (7.15) to simplify, we arrive at the expressions (7.2) for the coefficients ${\mathcal {I}}_{1}^{(1)}$, $I_{1}^{(1)}$, ${\mathcal {I}}_{1}^{(2)}$, $I_{1}^{(2)}$, ${\mathcal {I}}_{1}^{(3)}$, $I_{1}^{(3)}$. This completes the proof of Proposition 7.1. $\quad \square$

## Asymptotics of $I_{2}$

The large *s* asymptotics of $I_{2}$ is a consequence of the following three propositions whose proofs are given in Sects. 8.1, 8.2, and 8.3 , respectively.

### Proposition 8.1

(Splitting of $I_2$). The function $I_{2}$ defined in (6.11) can be written as

$$\begin{aligned} I_2 = X + Z, \end{aligned}$$

where *X* and *Z* are defined by

7.15b $$\begin{aligned} X = \int _{\Sigma _5} H'(\zeta ) \ln {\mathcal {G}}(\zeta )\frac{d\zeta }{2\pi i}, \qquad Z = -2 \int _{\gamma _{b_2b_1}} H'(\zeta ) p(\zeta )\frac{d\zeta }{2\pi i}. \end{aligned}$$

### Proposition 8.2

(Large *s* asymptotics of *X*). Let $\alpha > -1$. The quantity *X* defined in (8.1) admits the following asymptotic expansion as $s \rightarrow +\infty$:

8.1 $$\begin{aligned} X =&\; {\mathscr {X}}_{1}^{(2)} s^{\rho } \big ( \ln (s^{\rho })\big )^{2} +\big ({\mathcal {X}}_{1}^{(2)}+{\mathcal {X}}_{3}^{(2)} \big ) s^{\rho }\ln (s^{\rho }) + \big (X_{1}^{(2)}+X_{3}^{(2)}\big ) s^{\rho } \nonumber \\&+ \big ({\mathcal {X}}_{1}^{(3)} + {\mathcal {X}}_{3}^{(3)} \big ) \ln (s^{\rho }) + X_{1}^{(3)} + X_{2}^{(3)} + X_{3}^{(3)} + {{{\mathcal {O}}}}\big ( s^{-\rho }\ln (s^{\rho })\big ) \end{aligned}$$

uniformly for $\theta$ in compact subsets of (0, 1], where the coefficients are given by

8.2 $$\begin{aligned} {\mathscr {X}}_{1}^{(2)}&= \frac{(\alpha +1) (\theta -1) (b_1-b_2)}{4 \pi \theta ^3}, \nonumber \\ {\mathcal {X}}_{1}^{(2)}&= \frac{\alpha \theta \Big ( b_1 \ln (i b_1)-b_2 \ln (i b_2)\Big )+((\alpha +2) \theta -2 (\alpha +1)) \left( b_1 \ln \left( -\frac{i b_1}{\theta }\right) -b_2 \ln \left( -\frac{i b_2}{\theta }\right) \right) }{4 \pi \theta ^3}, \nonumber \\ X_{1}^{(2)}&= \frac{1}{4 \pi \theta ^{3}}\bigg \{b_{1} \ln (-\frac{ib_{1}}{\theta })\Big ( \alpha \theta \ln (ib_{1})-(1+\alpha -\theta )\ln (-\frac{ib_{1}}{\theta }) \Big ) \nonumber \\&\quad -b_{2} \ln (-\frac{ib_{2}}{\theta })\Big ( \alpha \theta \ln (ib_{2})-(1+\alpha -\theta )\ln (-\frac{ib_{2}}{\theta }) \Big ) \bigg \}, \nonumber \\ {\mathcal {X}}_{1}^{(3)}&= \frac{i (\alpha +1) \left( \alpha \theta \Big ( \ln (i b_1)-\ln (i b_2)\Big )+((\alpha +2) \theta -2 (\alpha +1)) \left( \ln \left( -\frac{i b_1}{\theta }\right) -\ln \left( -\frac{i b_2}{\theta }\right) \right) \right) }{8 \pi \theta ^3}, \nonumber \\ X_{1}^{(3)}&= \frac{i}{48 \pi \theta ^{3}} \bigg \{ 6 \alpha \theta (1+\alpha - \theta )\big ( \ln (ib_{1})-\ln (ib_{2}) \big ) \nonumber \\&\quad - \Big ( 9(1+\alpha )^{2} + 8 \theta ^{2} + \theta (3\alpha (\alpha -6)-19) \Big ) \Big ( \ln \Big ( \tfrac{-ib_{1}}{\theta } \Big )-\ln \Big ( \tfrac{-ib_{2}}{\theta } \Big ) \Big ) \nonumber \\&\quad + 6 \alpha \theta (1+\alpha )\Big ( \ln (ib_{1})\ln \Big ( \tfrac{-ib_{1}}{\theta } \Big ) - \ln (ib_{2})\ln \Big ( \tfrac{-ib_{2}}{\theta } \Big ) \Big ) \nonumber \\&\quad - 6 (1+\alpha )(1+\alpha -\theta ) \Big ( \Big ( \ln \big ( \tfrac{-ib_{1}}{\theta } \big )\Big )^{2} -\Big (\ln \big ( \tfrac{-ib_{2}}{\theta } \big )\Big )^{2} \Big ) \bigg \}, \end{aligned}$$

8.3 $$\begin{aligned} X_{2}^{(3)}&= \; \frac{\alpha }{2\theta } + \sum _{k=1}^{\infty } \bigg \{ -k\psi \left( 1+\alpha +k \theta \right) + k\ln \left( \frac{1+\alpha }{2}+ k \theta \right) +\frac{\alpha }{2\theta }\nonumber \\&\quad + \frac{1-6 \alpha - 9 \alpha ^{2}}{24 \theta ^{2}} \ln \left( 1+\frac{1}{k} \right) \bigg \}, \end{aligned}$$

and

8.4 $$\begin{aligned} {\mathcal {X}}_{3}^{(2)} =&- \frac{i(\alpha +1) (\theta -1) }{2 \theta ^3} \frac{b_1 - b_2}{2\pi i}, \nonumber \\ X_{3}^{(2)} =&- \frac{i(\alpha +1) (\theta -1) }{2 \theta ^3} \frac{b_2 - b_1 + b_1\ln (-\frac{i b_1}{\theta }) - b_2 \ln (-\frac{ib_2}{\theta })}{2\pi i}, \nonumber \\ {\mathcal {X}}_{3}^{(3)} =&\; \frac{\theta \left( 9 \alpha ^2+6 \alpha -1\right) -3 (\alpha +1)^2+2 \theta ^2 }{24 \theta ^3} \frac{\ln (b_1/b_2)}{2 \pi i}, \nonumber \\ X_{3}^{(3)} =&\; \frac{\theta \left( 9 \alpha ^2+6 \alpha -1\right) -3 (\alpha +1)^2+2 \theta ^2}{24 \theta ^3} \frac{\ln (\frac{b_1}{b_2}) \ln (-\frac{b_1b_2}{\theta ^2})}{4\pi i} \nonumber \\&\; + \frac{6 (\alpha +1) (\theta -1) (\alpha -\theta +1)}{24 \theta ^3} \frac{\ln (b_1/b_2)}{2 \pi i}. \end{aligned}$$

### Proposition 8.3

(Large *s* asymptotics of *Z*). Let $\alpha > -1$. The quantity *Z* defined in (8.1) admits the following asymptotic expansion as $s \rightarrow +\infty$:

8.5 $$\begin{aligned} Z = {\mathscr {Z}}^{(2)} s^\rho (\ln (s^\rho ))^2 + {\mathcal {Z}}^{(2)} s^\rho \ln (s^\rho ) + Z^{(2)} s^\rho + {\mathcal {Z}}^{(3)} \ln (s^\rho ) + Z^{(3)} + {{{\mathcal {O}}}}(s^{-\rho } \ln (s^\rho )) \end{aligned}$$

uniformly for $\theta$ in compact subsets of (0, 1], where the coefficients ${\mathscr {Z}}^{(2)}$, ${\mathcal {Z}}^{(2)}$, $Z^{(2)}$, ${\mathcal {Z}}^{(3)}$, $Z^{(3)}$ are given by

$$\begin{aligned} {\mathscr {Z}}^{(2)} =&-\frac{c_4}{2\pi \theta ^2 \rho } (b_1 - b_2), \\ {\mathcal {Z}}^{(2)} =&\; \frac{1}{2 \pi \theta ^2}\bigg \{\pi |b_2| (c_5-c_6)+\frac{b_2 c_4 \ln \left( -\frac{i b_2}{\theta }\right) -b_1 c_4 \ln \left( -\frac{ib_1}{\theta }\right) }{\rho }\\&\quad +(b_1-b_2)(c_5+c_6-c_7) \\&+\frac{i \pi }{2} \Big (b_1 (3c_5+c_6)+b_2 (c_5-c_6)\Big ) -b_1 \ln (b_1)(c_5+c_6) \\&+b_2 \ln (b_2) (c_5+c_6)+\pi \mathrm{Im}\,(b_2) (c_5+c_6)\bigg \},\\ Z^{(2)} =&\; \frac{1}{4\pi \theta ^{2}} \bigg \{ -2 (b_{1}-b_{2})\Big ( 2(c_{5}+c_{6})-c_{7} \Big )\\&\quad - i \pi \Big ( (-4 i |b_{2}| + 3b_{1}+b_{2})c_{5} + (b_{1}-b_{2})c_{6} \Big ) \\&+ b_{1} \ln (b_{1}) \Big ( 4c_{5}+4c_{6}-2c_{7}+ \pi i (3c_{5}+c_{6}) \Big ) - 2 b_{1} (\ln {b_{1}})^2(c_{5}+c_{6}) \\&+b_{2} \ln (b_{2}) \Big ( -4c_{5}-4c_{6}+2c_{7} + \pi i (c_{5}-c_{6})+2(c_{5}+c_{6})\ln (b_{2}) \Big ) \\&+\pi \Big ( 2 |b_{2}|(c_{5}-c_{6})-i(b_{1}+b_{2})(c_{5}+c_{6}) \Big ) \ln \left( \frac{i(|b_{2}|+\mathrm{Im}\,(b_{2}))}{2} \right) \\&+ 2 \left( 1+\ln (\tfrac{-i}{\theta })\right) \bigg [ (b_{1}-b_{2})(c_{5}+c_{6}-c_{7}) + |b_{2}| (c_{5}-c_{6}) \pi \\&+ \frac{\pi i}{2}\Big ( b_{2}(c_{5}-c_{6})+b_{1}(3c_{5}+c_{6}) \Big ) + \pi \mathrm{Im}\,(b_{2}) (c_{5}+c_{6}) \\&- b_{1} \ln (b_{1})(c_{5}+c_{6}) + b_{2} \ln (b_{2})(c_{5}+c_{6}) \bigg ] \bigg \}, \\ {\mathcal {Z}}^{(3)} =&\; \frac{2 \pi \big (c_8-\frac{3\alpha ^2-1}{12}\big ) \rho (\mathrm{Im}\,(b_2)-|b_2|)-i |b_2| (\ln (b_1)-\ln (b_2)) (\alpha c_4+c_4-2 c_8 \rho )}{4 \pi |b_2| \theta ^2 \rho }, \end{aligned}$$

and

$$\begin{aligned} Z^{(3)} =&\; \frac{1}{8 \pi |b_2| \theta ^2}\bigg \{4 \pi \bigg (c_8-\frac{3\alpha ^2-1}{12}\bigg ) \bigg [-|b_2| \ln \left( \frac{2 i |b_2|^2}{|b_2|+\mathrm{Im}\,(b_2)}\right) \\&+\mathrm{Im}\,(b_2) \ln \left( \frac{1}{2} i (|b_2|+\mathrm{Im}\,(b_2))\right) +|b_2|-\mathrm{Im}\,(b_2)\bigg ] \\&+i (\alpha +1) |b_2| \bigg [-4 i \pi \ln (|b_2|) (c_5-c_6)+4 i \pi c_6 \ln \left( \frac{2}{|b_2|+\mathrm{Im}\,(b_2)}\right) \\&+\ln (b_1) \Big (-2 c_7+i \pi (3 c_5+c_6)\Big )-(\ln {b_1})^2 (c_5+c_6) \\&+\ln (b_2) \Big (\ln (b_2) (c_5+c_6) +i \pi (c_5-c_6)+2 c_7\Big )+2 \pi ^2 c_5\bigg ] \\&+4 i \left( 1+\ln \Big (-\frac{i}{\theta }\Big )\right) \bigg (|b_2| c_8 (\ln (b_1)-\ln (b_2))\\&\quad +i \pi \bigg (c_8-\frac{3\alpha ^2-1}{12}\bigg ) (|b_2|-\mathrm{Im}\,(b_2))\bigg ) \\&+2 i |b_2| c_8 \left( (\ln {b_1})^2-(\ln {b_2})^2\right) \bigg \}. \end{aligned}$$

### Proof of Proposition 8.1

Recall that the integral $I_{2}$ is given by

$$\begin{aligned} I_{2}&= \frac{1}{2} \int _{\sigma } H(\zeta ) {{\,\mathrm{Tr}\,}}\Big [ P^{\infty }(\zeta )^{-1}P^{\infty }(\zeta )' \sigma _{3} \Big ] \frac{d\zeta }{2\pi i}, \end{aligned}$$

where *H* is given by (6.8) and $\sigma$ is a closed curve surrounding $\Sigma _{5}$ once in the positive direction which does not surround any of the poles $\zeta _{j}$ of *H*. A straightforward computation gives

$$\begin{aligned} {{\,\mathrm{Tr}\,}}\left[ P^{\infty }(\zeta )^{-1}P^{\infty \prime }(\zeta )\sigma _3 \right] =&\; {{\,\mathrm{Tr}\,}}\bigg [ e^{-p(\zeta ) \sigma _3} Q^\infty (\zeta )^{-1} e^{p_0\sigma _3} \Big (e^{-p_0\sigma _3} Q^{\infty \prime }(\zeta ) e^{p(\zeta ) \sigma _3} \\&+ e^{-p_0\sigma _3} Q^\infty (\zeta ) e^{p(\zeta ) \sigma _3} p'(\zeta ) \sigma _3\Big )\sigma _3 \bigg ] \\ =&\; {{\,\mathrm{Tr}\,}}\bigg [Q^\infty (\zeta )^{-1} Q^{\infty \prime }(\zeta ) \sigma _3 \bigg ] + {{\,\mathrm{Tr}\,}}\big [p'(\zeta ) I\big ] = 2 p'(\zeta ), \end{aligned}$$

where we have used that

$$\begin{aligned} Q^\infty (\zeta )^{-1} Q^{\infty \prime }(\zeta ) \sigma _3 = i\frac{\gamma '(\zeta )}{\gamma (\zeta )} \sigma _1 \end{aligned}$$

has trace zero in the last step. Hence

$$\begin{aligned}&I_{2} = \int _\sigma H(\zeta ) p'(\zeta )\frac{d\zeta }{2\pi i}. \end{aligned}$$

The function $p(\zeta )$ is analytic for $\zeta \in {\mathbb {C}}{\setminus } \Sigma _5$ and satisfies the following jump condition across $\Sigma _5$:

$$\begin{aligned} p_+(\zeta ) + p_-(\zeta ) = -\ln {\mathcal {G}}(\zeta ), \qquad \zeta \in \Sigma _5. \end{aligned}$$

Integrating by parts, deforming the contour, and using the jump condition for *p*, we find

$$\begin{aligned} I_{2}&= -\int _\sigma H'(\zeta ) p(\zeta )\frac{d\zeta }{2\pi i} = \int _{\Sigma _5} H'(\zeta ) (p_+(\zeta ) - p_-(\zeta ))\frac{d\zeta }{2\pi i} \nonumber \\&= \int _{\Sigma _5} H'(\zeta ) (2p_+(\zeta ) + \ln {\mathcal {G}}(\zeta ))\frac{d\zeta }{2\pi i}\nonumber \\&= -2 \int _{\gamma _{b_2b_1}} H'(\zeta ) p(\zeta )\frac{d\zeta }{2\pi i} + \int _{\Sigma _5} H'(\zeta ) \ln {\mathcal {G}}(\zeta )\frac{d\zeta }{2\pi i} = Z + X, \end{aligned}$$

which completes the proof.

### Proof of Proposition 8.2

An integration by parts gives

$$\begin{aligned} X =&\; \frac{H(\zeta ) \ln {\mathcal {G}}(\zeta )}{2\pi i}\bigg |_{\zeta = b_1}^{b_2} - \int _{\Sigma _5} H(\zeta ) \frac{{\mathcal {G}}'(\zeta )}{{\mathcal {G}}(\zeta )} \frac{d\zeta }{2\pi i}. \end{aligned}$$

From the expression (2.17) for $\ln {\mathcal {G}}$, we have

$$\begin{aligned} \frac{{\mathcal {G}}'(\zeta )}{{\mathcal {G}}(\zeta )} =&-is^\rho \bigg \{c_1 +c_2+c_3 + c_1\ln (i\zeta ) + c_2\ln (-i\zeta ) + \ln (s)\\&- \psi \bigg (\frac{1+\alpha }{2} + is^\rho \zeta \bigg ) - \frac{1}{\theta }\psi \bigg (\frac{\frac{1+\alpha }{2} - is^\rho \zeta }{\theta }\bigg ) \bigg \}\\ =&\; i s^\rho \bigg \{\psi \bigg (\frac{1+\alpha }{2} + is^\rho \zeta \bigg ) - \ln (i \zeta s^\rho ) + \frac{\psi (\frac{\frac{1+\alpha }{2} - is^\rho \zeta }{\theta }) - \ln (-\frac{i \zeta s^\rho }{\theta })}{\theta }\bigg \}, \end{aligned}$$

where $c_{1}$, $c_{2}$, and $c_{3}$ are given by (2.4). Thus,

$$\begin{aligned} X =&\; \frac{H(\zeta ) \ln {\mathcal {G}}(\zeta )}{2\pi i}\bigg |_{\zeta = b_1}^{b_2} - i s^\rho \int _{\Sigma _5} \bigg \{ \frac{1}{\theta ^{2}} \left( \frac{1+\alpha }{2}- i s^{\rho }\zeta \right) \psi \bigg (\frac{\frac{1+\alpha }{2}-is^{\rho }\zeta }{\theta } \bigg )\bigg \}\\&\times \bigg \{\psi \bigg (\frac{1+\alpha }{2} + is^\rho \zeta \bigg ) - \ln (i \zeta s^\rho ) + \frac{\psi (\frac{\frac{1+\alpha }{2} - is^\rho \zeta }{\theta }) - \ln (-\frac{i \zeta s^\rho }{\theta })}{\theta }\bigg \}\frac{d\zeta }{2\pi i}\\ =&\; \frac{H(\zeta ) \ln {\mathcal {G}}(\zeta )}{2\pi i}\bigg |_{\zeta = b_1}^{b_2} - \int _{i s^\rho \Sigma _5} \bigg \{ \frac{1}{\theta ^{2}} \left( \frac{1+\alpha }{2}- w \right) \psi \bigg (\frac{\frac{1+\alpha }{2}- w}{\theta } \bigg )\bigg \}\\&\times \bigg \{\psi \bigg (\frac{1+\alpha }{2} + w\bigg ) - \ln (w) + \frac{\psi (\frac{\frac{1+\alpha }{2} - w}{\theta }) - \ln (-\frac{w}{\theta })}{\theta }\bigg \}\frac{dw}{2\pi i}, \end{aligned}$$

where we have changed variables to $w = is^\rho \zeta$ in the second step. The function $\psi (\frac{\frac{1+\alpha }{2} - w}{\theta })$ has poles at the points $w = \frac{1+\alpha }{2} + j\theta \in (0,\infty )$, $j = 0,1,2, \dots$, and the function $\psi (\frac{1+\alpha }{2} + w)$ has poles at the points $w = -\frac{1+\alpha }{2} - j \in (-\infty ,0)$, $j = 0,1,2, \dots$. Thus the term which will cause the most difficulties in the analysis is the one involving the product $\psi (\frac{\frac{1+\alpha }{2} - w}{\theta })\psi (\frac{1+\alpha }{2} + w)$ (for the other terms we can deform the contour into the left half-plane and use the large *z* asymptotics of $\psi (z)$).

Let $m = \frac{1+\alpha }{2}$; the exact value of *m* is not essential as long as $m > 0$. We split *X* as follows:

8.6 $$\begin{aligned} X =&\; X_1 + X_2 + X_3, \end{aligned}$$

where

8.7 $$\begin{aligned} X_1 =&\; \frac{H(\zeta ) \ln {\mathcal {G}}(\zeta )}{2\pi i}\bigg |_{\zeta = b_1}^{b_2}, \end{aligned}$$

8.8 $$\begin{aligned} X_2 =&- \int _{i s^\rho \Sigma _5} \frac{1}{\theta ^{2}} \left( \frac{1+\alpha }{2}- w \right) \psi \bigg (\frac{\frac{1+\alpha }{2}- w}{\theta } \bigg ) \bigg \{\psi \bigg (\frac{1+\alpha }{2} + w\bigg ) - \ln (w) +{\hat{g}}(w) \bigg \}\frac{dw}{2\pi i}, \end{aligned}$$

8.9 $$\begin{aligned} X_3 =&- \int _{i s^\rho \Sigma _5} \frac{1}{\theta ^{2}} \left( \frac{1+\alpha }{2}- w \right) \psi \bigg (\frac{\frac{1+\alpha }{2}- w}{\theta } \bigg ) \bigg \{ \frac{\psi (\frac{\frac{1+\alpha }{2} - w}{\theta }) - \ln (-\frac{w}{\theta })}{\theta } -{\hat{g}}(w)\bigg \}\frac{dw}{2\pi i}, \end{aligned}$$

and the function ${\hat{g}}(w)$ is defined by

8.10 $$\begin{aligned} {\hat{g}}(w) = - \frac{\alpha }{2} \left( \frac{1}{w-m} - \frac{m}{(w-m)^{2}} \right) - \frac{1-3\alpha ^{2}}{24(w-m)^{2}}. \end{aligned}$$

Note that all the *s*-dependence of $X_{2}$ and $X_{3}$ is in the contour. The term ${\hat{g}}(w)$ has been added and subtracted so that the integrand in the definition of $X_2$ is ${{{\mathcal {O}}}}(w^{-2} \ln w)$ as $w \rightarrow \infty$. This can be verified by using the asymptotic expansion (see [27, Eq. 5.11.2])

8.11 $$\begin{aligned} \psi (z) \sim \ln z - \frac{1}{2z} - \sum _{k=1}^\infty \frac{B_{2k}}{2k z^{2k}}, \qquad z \rightarrow \infty , \ |\arg z| \le \pi - \delta , \end{aligned}$$

where $B_{2k}$ is the 2*k*th Bernoulli number, which implies that

8.12 $$\begin{aligned} \psi \bigg (\frac{1+\alpha }{2} + w\bigg ) =&\; \ln \bigg (\frac{1+\alpha }{2} + w\bigg ) - \frac{1}{2(\frac{1+\alpha }{2} + w)} - \frac{1}{12(\frac{1+\alpha }{2} + w)^2} + {{{\mathcal {O}}}}(w^{-4}) \nonumber \\ =&\; \ln {w} + \frac{\alpha }{2w} + \frac{1 - 3\alpha ^2}{24 w^2} + {{{\mathcal {O}}}}(w^{-3}) \end{aligned}$$

as $w \rightarrow \infty$ away from the negative real axis, as well as the expansion

8.13 $$\begin{aligned} {\hat{g}}(w) = - \frac{\alpha }{2w} - \frac{1-3\alpha ^{2}}{24w^{2}} + {{{\mathcal {O}}}}(w^{-3}) \qquad \text{ as } w \rightarrow \infty . \end{aligned}$$

The integrals defining $X_2$ and $X_3$ converge because the function ${\hat{g}}(w)$ is analytic except for a double pole at $w = m > 0$.

We will show that $X_1, X_2, X_3$ satisfy the large *s* asymptotics

8.14 $$\begin{aligned}&X_{1} = {\mathscr {X}}_{1}^{(2)} s^\rho (\ln (s^\rho ))^2 + {\mathcal {X}}_{1}^{(2)} s^\rho \ln (s^\rho ) + X_{1}^{(2)} s^\rho + {\mathcal {X}}_{1}^{(3)} \ln (s^\rho ) + X_{1}^{(3)} + {{{\mathcal {O}}}}\big (s^{-\rho }\ln (s^{\rho })\big ), \end{aligned}$$

8.15 $$\begin{aligned}&X_{2} = X_{2}^{(3)} + {{{\mathcal {O}}}}(s^{-\rho } \ln (s^{\rho })), \end{aligned}$$

8.16 $$\begin{aligned}&X_3 = {\mathcal {X}}_{3}^{(2)} s^{\rho } \ln (s^{\rho }) + X_{3}^{(2)} s^{\rho } + {\mathcal {X}}_{3}^{(3)} \ln (s^{\rho }) + X_{3}^{(3)} + {{{\mathcal {O}}}}\big (s^{-\rho }\ln (s^{\rho })\big ), \end{aligned}$$

uniformly for $\theta$ in compact subsets of (0, 1], where the coefficients of the three expansions are given by (8.3), (8.4), and (8.5), respectively. This will complete the proof of Proposition 8.2.

#### Asymptotics of $X_{1}$

For $\zeta \in {\mathbb {C}}$ bounded away from $i {\mathbb {R}}$, we have the expansions (see (3.1), (6.8), and (8.12))

$$\begin{aligned} \ln {\mathcal {G}}(\zeta ) = \frac{c_4}{\rho } \ln (s^\rho ) + c_5 \ln (i\zeta ) + c_6 \ln (-i\zeta ) + c_7 + \frac{c_8}{i\zeta s^\rho } + {{{\mathcal {O}}}}(s^{-2\rho }), \qquad s \rightarrow +\infty , \end{aligned}$$

and

8.17 $$\begin{aligned} H(\zeta ) = -\frac{i \zeta \ln \left( -\frac{i \zeta s^\rho }{\theta }\right) }{\theta ^2} s^\rho + \frac{\alpha -\theta +(\alpha +1) \ln \left( -\frac{i \zeta s^\rho }{\theta }\right) +1}{2 \theta ^2} + {{{\mathcal {O}}}}(s^{-\rho }), \qquad s \rightarrow +\infty , \end{aligned}$$

where the constants $c_j$ are given by (2.4). Substituting these expansions into the definition (8.8) of $X_1$, we find (8.15).

#### Asymptotics of $X_{2}$

Since the integrand in the definition of $X_2$ is ${{{\mathcal {O}}}}(w^{-2} \ln w)$ as $w \rightarrow \infty$, we see that $X_2$ satisfies (8.16) with

8.18 $$\begin{aligned} X_{2}^{(3)}&= - \int _{-i \infty }^{i \infty } \left\{ \frac{1}{\theta ^{2}}\left( \frac{1+\alpha }{2} - w \right) \psi \left( \frac{\frac{1+\alpha }{2}-w}{\theta } \right) \right\} \nonumber \\&\quad \times \left\{ \psi \left( \frac{1+\alpha }{2}+w \right) - \ln w +{\hat{g}}(w) \right\} \frac{dw}{2\pi i}, \end{aligned}$$

where the contour crosses the real line at 0 and ${\hat{g}}(w)$ is given by (8.11). It remains to show that $X_{2}^{(3)}$ can be written as in (8.4).

Since $\psi (z) = \frac{-1}{z+k} + {{{\mathcal {O}}}}\big ( (z+k)^{-2} \big )$ as $z \rightarrow -k$ for each $k = 0,1,2, \dots$, it follows that

$$\begin{aligned} \psi \left( \frac{\frac{1+\alpha }{2}-w}{\theta } \right) \end{aligned}$$

has a simple pole with residue $\theta$ at each of the points $\frac{1+\alpha }{2}+ k \theta$, $k = 0,1,2, \dots$. For $k \ge 1$, the associated residue gives a contribution to $X_{2}^{(3)}$ equal to (taking into account that after the deformation, the loop is going in the clockwise orientation around $\frac{1+\alpha }{2}+ k \theta$)

$$\begin{aligned} -k \left\{ \psi \left( 1+\alpha +k \theta \right) - \ln \left( \frac{1+\alpha }{2}+ k \theta \right) + {\hat{g}}\left( \frac{1+\alpha }{2}+ k \theta \right) \right\} . \end{aligned}$$

On the other hand, the residue at $m = \frac{1+\alpha }{2}$ is given by

$$\begin{aligned} -\frac{(1-6\alpha - 9 \alpha ^{2}) \gamma _{\mathrm {E}} - 12 \alpha \theta }{24 \theta ^{2}}, \end{aligned}$$

where $\gamma _{\mathrm {E}}$ is Euler’s gamma constant. By (8.13) and (8.14), we have

$$\begin{aligned} \psi \left( \frac{1+\alpha }{2}+w \right) - \ln w +{\hat{g}}(w) = {{{\mathcal {O}}}}(w^{-3}) \end{aligned}$$

as $w \rightarrow \infty$ away from the negative real axis. Moreover, by (8.12),

8.19 $$\begin{aligned} \psi \bigg (\frac{\frac{1+\alpha }{2} - w}{\theta }\bigg ) =&\; \ln \bigg (-\frac{w}{\theta }\bigg ) + O(w^{-1}), \end{aligned}$$

as $w \rightarrow \infty$ away from the positive real axis; in fact, combining (8.12) with the reflection formula $\psi (1-z) = \psi (z) + \pi \cot (\pi z)$, we see that (8.20) holds also as $w \rightarrow \infty$ in a sector containing the positive real axis as long as *w* stays away from the poles $\{\frac{1+\alpha }{2} + j\theta \}_{j=0}^\infty$. Thus, deforming the contour in (8.19) to infinity in the right half-plane along curves which stay away from the set $\{\frac{1+\alpha }{2} + j\theta \}_{j=0}^\infty$, the contribution from infinity vanishes and we find

$$\begin{aligned} X_{2}^{(3)} =&-\frac{(1-6\alpha - 9 \alpha ^{2}) \gamma _{\mathrm {E}} - 12 \alpha \theta }{24 \theta ^{2}} \\&+ \sum _{k=1}^{\infty } k \left\{ -\psi \left( 1+\alpha +k \theta \right) + \ln \left( \frac{1+\alpha }{2}+ k \theta \right) -{\hat{g}}\left( \frac{1+\alpha }{2}+ k \theta \right) \right\} , \end{aligned}$$

where the series is convergent because it originates from a convergent integral. Using the series representation for the Euler gamma constant (see [27, Eq. 5.2.3])

$$\begin{aligned} \gamma _{\mathrm {E}} = \sum _{k=1}^{\infty } \left\{ \frac{1}{k}- \ln \left( 1+\frac{1}{k} \right) \right\} \end{aligned}$$

together with the fact that

$$\begin{aligned} -k{\hat{g}}\left( \frac{1+\alpha }{2}+ k \theta \right) = \frac{1-6 \alpha - 9 \alpha ^{2}}{24 k \theta ^{2}} + \frac{\alpha }{2 \theta }, \end{aligned}$$

we conclude that $X_{2}^{(3)}$ can be written as in (8.4). This proves (8.16).

#### Asymptotics of $X_{3}$

Deforming the contour $is^{\rho }\Sigma _{5}$ in the definition (8.10) of $X_3$ in the left half-plane to the contour $is^{\rho }\gamma _{b_{2}b_{1}}$, we get

8.20 $$\begin{aligned} X_3 =&\int _{i s^\rho \gamma _{b_2b_1}} \frac{1}{\theta ^{2}} \left( \frac{1+\alpha }{2}- w \right) \psi \bigg (\frac{\frac{1+\alpha }{2}- w}{\theta } \bigg ) \bigg \{ \frac{\psi (\frac{\frac{1+\alpha }{2} - w}{\theta }) - \ln (-\frac{w}{\theta })}{\theta } -{\hat{g}}(w)\bigg \}\frac{dw}{2\pi i}. \end{aligned}$$

The above representation is convenient for the asymptotic analysis of $X_3$, because the argument $(\frac{1+\alpha }{2}- w)/\theta$ of $\psi$ is large as $s \rightarrow + \infty$ uniformly for $w \in i s^\rho \gamma _{b_2b_1}$. As $w \rightarrow \infty$ away from the positive real axis, we have

8.21 $$\begin{aligned} \psi \bigg (\frac{\frac{1+\alpha }{2} - w}{\theta }\bigg ) =&\; \ln (-\frac{w}{\theta }) + \frac{\theta -1-\alpha }{2w} + \frac{6\theta - 2\theta ^2 - 3- 3\alpha ^2 + 6\alpha (\theta -1)}{24 w^2} + {{{\mathcal {O}}}}(w^{-3}). \end{aligned}$$

Substitution of the expansions (8.22) and (8.14) into (8.21) yields

$$\begin{aligned} X_3 =&\int _{i s^\rho \gamma _{b_2b_1}} \bigg \{-\frac{(\alpha +1) (\theta -1) \ln \left( -\frac{w}{\theta }\right) }{2 \theta ^3} \\&+\frac{6 (\alpha +1) (\theta -1) (\alpha -\theta +1)+\left( \left( 9 \alpha ^2+6 \alpha -1\right) \theta -3 (\alpha +1)^2+2 \theta ^2\right) \ln \left( -\frac{w}{\theta }\right) }{24 \theta ^3 w} \\&+ {{{\mathcal {O}}}}(w^{-2} \ln (-w))\bigg \}\frac{dw}{2\pi i}. \end{aligned}$$

Letting $w = i s^\rho \zeta$, we obtain

8.22 $$\begin{aligned} X_3 =&\int _{\gamma _{b_2b_1}} \bigg \{-\frac{(\alpha +1) (\theta -1) \ln \left( -\frac{ i s^\rho \zeta }{\theta }\right) }{2 \theta ^3} \nonumber \\&+\frac{6 (\alpha +1) (\theta -1) (\alpha -\theta +1)+\left( \left( 9 \alpha ^2+6 \alpha -1\right) \theta -3 (\alpha +1)^2+2 \theta ^2\right) \ln \left( -\frac{ i s^\rho \zeta }{\theta }\right) }{24 \theta ^3 i s^\rho \zeta } \bigg \}\frac{i s^\rho d\zeta }{2\pi i} \nonumber \\&+ {{{\mathcal {O}}}}(s^{-\rho } \ln (s^\rho )) \nonumber \\ =&\; {\mathcal {X}}_{3}^{(2)} s^{\rho } \ln (s^{\rho }) + X_{3}^{(2)} s^{\rho } + {\mathcal {X}}_{3}^{(3)} \ln (s^{\rho }) + X_{3}^{(3)} + {{{\mathcal {O}}}}\big (s^{-\rho }\ln (s^{\rho })\big ) \end{aligned}$$

uniformly for $\theta$ in compact subsets of (0, 1], where the coefficients are given by

$$\begin{aligned} {\mathcal {X}}_{3}^{(2)} =&- \frac{i(\alpha +1) (\theta -1) }{2 \theta ^3} \int _{\gamma _{b_2b_1}}\frac{d\zeta }{2\pi i}, \\ X_{3}^{(2)} =&- \frac{i(\alpha +1) (\theta -1) }{2 \theta ^3} \int _{\gamma _{b_2b_1}} \ln (-\tfrac{i \zeta }{\theta }) \frac{d\zeta }{2\pi i}, \\ {\mathcal {X}}_{3}^{(3)} =&\; \frac{ \left( 9 \alpha ^2+6 \alpha -1\right) \theta -3 (\alpha +1)^2+2 \theta ^2 }{24 \theta ^3} \int _{\gamma _{b_2b_1}} \frac{1}{\zeta } \frac{d\zeta }{2\pi i}, \\ X_{3}^{(3)} =&\; \frac{\left( 9 \alpha ^2+6 \alpha -1\right) \theta -3 (\alpha +1)^2+2 \theta ^2}{24 \theta ^3} \int _{\gamma _{b_2b_1}}\frac{\ln (-\frac{i \zeta }{\theta })}{\zeta } \frac{d\zeta }{2\pi i}. \end{aligned}$$

Using that

$$\begin{aligned}&\int _{\gamma _{b_2b_1}}\frac{d\zeta }{2\pi i} = \frac{b_1 - b_2}{2\pi i},\\&\int _{\gamma _{b_2b_1}} \ln (-\frac{i \zeta }{\theta })\frac{d\zeta }{2\pi i} = \frac{b_2 - b_1 + b_1\ln (-\frac{i b_1}{\theta }) - b_2 \ln (-\frac{ib_2}{\theta })}{2\pi i},\\&\int _{\gamma _{b_2b_1}} \frac{1}{\zeta } \frac{d\zeta }{2\pi i} = \frac{\ln (b_1/b_2)}{2 \pi i} = \frac{\pi - 2\arg (b_2)}{2\pi },\\&\int _{\gamma _{b_2b_1}}\frac{\ln (-\frac{i \zeta }{\theta })}{\zeta } \frac{d\zeta }{2\pi i} = \frac{\ln (\frac{b_1}{b_2}) \ln (-\frac{b_1b_2}{\theta ^2})}{4\pi i}, \qquad \end{aligned}$$

we see that the coefficients ${\mathcal {X}}_{3}^{(2)}, X_{3}^{(2)}, {\mathcal {X}}_{3}^{(3)}, X_{3}^{(3)}$ can be written as in (8.5). This proves (8.17) and thus completes the proof of Proposition 8.2.

### Proof of Proposition 8.3

We have

$$\begin{aligned} H'(\zeta ) = -\frac{i}{\theta ^2}s^\rho \ln (s^\rho ) -\frac{i(1 + \ln (-\frac{i\zeta }{\theta }))}{\theta ^2}s^\rho + \frac{1+\alpha }{2\zeta \theta ^2} + {{{\mathcal {O}}}}(s^{-\rho }), \qquad s \rightarrow +\infty , \end{aligned}$$

uniformly for $\zeta \in \gamma _{b_2b_1}$. Moreover, by Proposition 3.2,

$$\begin{aligned} p(\zeta ) = -\frac{c_4}{2\rho } \ln (s^\rho ) + \frac{{\mathcal {B}}(\zeta )}{2} + \frac{{\mathcal {A}}(\zeta )}{s^\rho } + {{{\mathcal {O}}}}(s^{-2\rho }), \qquad s \rightarrow +\infty , \end{aligned}$$

uniformly for $\zeta \in \gamma _{b_2b_1}$, where the coefficients ${\mathcal {B}}(\zeta )$ and ${\mathcal {A}}(\zeta )$ are defined by

8.23 $$\begin{aligned} {\mathcal {A}}(\zeta ) =&\; \frac{{\mathcal {A}}_1(\zeta )}{\zeta }= \frac{ic_8}{2\zeta }+\frac{c_8-\frac{3\alpha ^2-1}{12}}{2|b_2|} \frac{r(\zeta )}{\zeta }, \end{aligned}$$

8.24 $$\begin{aligned} {\mathcal {B}}(\zeta ) =&\; {\mathcal {R}}(\zeta ) - c_{7} - c_{5} \ln (i\zeta ) - c_{6} \ln (-i\zeta ), \end{aligned}$$

with ${\mathcal {A}}_1$ and ${\mathcal {R}}$ given by (3.4) and (3.3). Substitution into the definition (8.1) of *Z* shows that *Z* admits an expansion of the form (8.6) as $s \rightarrow + \infty$, uniformly for $\theta$ in compact subsets of (0, 1], with coefficients given by

8.25 $$\begin{aligned} {\mathscr {Z}}^{(2)} =&-\frac{c_4}{2\pi \theta ^2 \rho } (b_1 - b_2) = \frac{(\alpha +1) (\theta -1) \theta ^{\frac{2}{\theta +1}-\frac{5}{2}}}{\pi (\theta +1) \rho }, \nonumber \\ {\mathcal {Z}}^{(2)} =&\int _{\gamma _{b_2b_1}} \frac{-c_4(1 + \ln (-\frac{i\zeta }{\theta }))+\rho {\mathcal {B}}(\zeta )}{2 \pi \theta ^2 \rho } d\zeta \nonumber \\ =&- \frac{c_4 }{2 \pi \theta ^2 \rho } \bigg (b_1\ln (-\frac{i b_1}{\theta }) - b_2\ln (-\frac{ib_2}{\theta })\bigg ) + \frac{1}{2 \pi \theta ^2} \int _{\gamma _{b_2b_1}} {\mathcal {B}}(\zeta ) d\zeta , \nonumber \\ Z^{(2)} =&\; \int _{\gamma _{b_2b_1}} \frac{1+\ln (-\frac{i \zeta }{\theta })}{2 \pi \theta ^2} {\mathcal {B}}(\zeta )d\zeta \nonumber \\ =&\; \frac{1+\ln (-\frac{i}{\theta })}{2 \pi \theta ^2} \int _{\gamma _{b_2b_1}} {\mathcal {B}}(\zeta )d\zeta + \frac{1}{2 \pi \theta ^2} \int _{\gamma _{b_2b_1}} \ln (\zeta ) {\mathcal {B}}(\zeta )d\zeta , \nonumber \\ {\mathcal {Z}}^{(3)} =&\; \int _{\gamma _{b_2b_1}} \bigg \{ \frac{{\mathcal {A}}(\zeta )}{\pi \theta ^2}-\frac{i (\alpha +1) c_4}{4 \pi \zeta \theta ^2 \rho }\bigg \} d\zeta \nonumber \\ =&\; \frac{1}{\pi \theta ^2} \int _{\gamma _{b_2b_1}} {\mathcal {A}}(\zeta )d\zeta - \frac{i (\alpha +1) c_4}{4 \pi \theta ^2 \rho } (\ln {b_1} - \ln {b_2}), \nonumber \\ Z^{(3)} =&\; \int _{\gamma _{b_2b_1}} \bigg \{ \frac{1+\ln {\zeta } + \ln (-\frac{i}{\theta })}{\pi \theta ^2}{\mathcal {A}}(\zeta ) + \frac{i (\alpha +1) {\mathcal {B}}(\zeta )}{4 \pi \zeta \theta ^2}\bigg \}d\zeta \nonumber \\ =&\; \frac{1+ \ln (-\frac{i}{\theta })}{\pi \theta ^2} \int _{\gamma _{b_2b_1}}{\mathcal {A}}(\zeta ) d\zeta + \frac{1}{\pi \theta ^2} \int _{\gamma _{b_2b_1}} \ln ({\zeta }) {\mathcal {A}}(\zeta ) d\zeta \nonumber \\&\qquad + \frac{i (\alpha +1)}{4 \pi \theta ^2} \int _{\gamma _{b_2b_1}} \frac{{\mathcal {B}}(\zeta )}{\zeta } d\zeta . \end{aligned}$$

It only remains to show that the coefficients in (8.26) can be expressed as in the statement of Proposition 8.3. Inspection of (8.26) shows that there are five different integrals that need to evaluated:

$$\begin{aligned}&\int _{\gamma _{b_2b_1}} {\mathcal {B}}(\zeta ) d\zeta , \quad \int _{\gamma _{b_2b_1}} \ln (\zeta ) {\mathcal {B}}(\zeta )d\zeta , \quad \int _{\gamma _{b_2b_1}} {\mathcal {A}}(\zeta ) d\zeta , \\&\qquad \int _{\gamma _{b_2b_1}} \ln (\zeta ) {\mathcal {A}}(\zeta ) d\zeta , \quad \int _{\gamma _{b_2b_1}} \frac{{\mathcal {B}}(\zeta )}{\zeta } d\zeta . \end{aligned}$$

These integrals are evaluated in the following lemma.

#### Lemma 8.4

For $\alpha > -1$ and $\theta \in (0,1]$, it holds that

8.26 $$\begin{aligned} \int _{\gamma _{b_2b_1}} {\mathcal {B}}(\zeta ) d\zeta =&\; \pi |b_2| (c_5-c_6)+(b_1-b_2) (c_5+c_6-c_7) \nonumber \\&+ \frac{ \pi i}{2} \Big (b_1 (3 c_5+c_6)+b_2 (c_5-c_6)\Big )-b_1 \ln (b_1) (c_5+c_6) \nonumber \\&+b_2 \ln (b_2) (c_5+c_6)+\pi \mathrm{Im}\,(b_2) (c_5+c_6), \end{aligned}$$

8.27a $$\begin{aligned} \int _{\gamma _{b_2b_1}} \ln (\zeta ) {\mathcal {B}}(\zeta ) d\zeta =&\; \frac{1}{2} \bigg \{\pi \ln \left( \frac{1}{2} i (|b_2|+\mathrm{Im}\,(b_2))\right) \Big (2 |b_2| (c_5-c_6)-i (b_1+b_2) (c_5+c_6)\Big ) \nonumber \\&-i \pi \Big (c_6 (b_1-b_2)+c_5 (-4 i |b_2|+3 b_1+b_2)\Big ) \nonumber \\&-2 (b_1-b_2) (2 (c_5+c_6)-c_7) \nonumber \\&+b_1 \ln (b_1) \Big (i \pi (3 c_5+c_6)+4 c_5+4 c_6-2 c_7\Big ) -2 b_1 (\ln {b_1})^2 (c_5+c_6) \nonumber \\&+b_2 \ln (b_2) \Big (2 \ln (b_2) (c_5+c_6)+i \pi (c_5-c_6)-4 c_5-4 c_6+2 c_7\Big )\bigg \}, \end{aligned}$$

8.27b $$\begin{aligned} \int _{\gamma _{b_2b_1}} {\mathcal {A}}(\zeta ) d\zeta =&-\frac{c_8(\arg b_1 - \arg b_2)}{2} - \frac{\pi \big (c_8-\frac{3\alpha ^2-1}{12}\big )}{2}\bigg (1 - \frac{\mathrm{Im}\,{b_2}}{|b_2|}\bigg ), \end{aligned}$$

8.27c $$\begin{aligned} \int _{\gamma _{b_2b_1}} \ln (\zeta ) {\mathcal {A}}(\zeta ) d\zeta =&\; \frac{ic_8}{4}((\ln {b_1})^2 - (\ln {b_2})^2) \nonumber \\&+ \frac{\pi \big (c_8-\frac{3\alpha ^2-1}{12}\big )}{2|b_2|}\bigg \{|b_2| - \mathrm{Im}\,{b_2} - |b_2| \ln \bigg (\frac{2i|b_2|^2}{|b_2| + \mathrm{Im}\,{b_2}}\bigg ) \nonumber \\&+ (\mathrm{Im}\,{b_2})\ln \bigg (\frac{i(|b_2| + \mathrm{Im}\,{b_2})}{2}\bigg )\bigg \}, \end{aligned}$$

8.27d $$\begin{aligned} \int _{\gamma _{b_2b_1}} \frac{{\mathcal {B}}(\zeta )}{\zeta } d\zeta =&\; \frac{1}{2} \bigg \{-4 i \pi \ln (|b_2|) (c_5-c_6)+4 i \pi c_6 \ln \left( \frac{2}{|b_2|+\mathrm{Im}\,(b_2)}\right) \nonumber \\&+\ln (b_1) (-2 c_7+i \pi (3 c_5+c_6))-(c_5+c_6)(\ln {b_1})^2 \nonumber \\&+\ln (b_2) (2 c_7+i \pi (c_5-c_6))+ (c_5+c_6)(\ln {b_2})^2 +2 \pi ^2 c_5\bigg \}. \end{aligned}$$

#### Proof

See “Appendix D”. $$\quad \square $$

Substituting the results of Lemma 8.4 into (8.26), we obtain after simplification the expressions for the coefficients ${\mathscr {Z}}^{(2)}$, ${\mathcal {Z}}^{(2)}$, $Z^{(2)}$, ${\mathcal {Z}}^{(3)}$, and $Z^{(3)}$ given in the statement of Proposition 8.3. This completes the proof of Proposition 8.3.

## Asymptotics of $I_{3,K}$ and $I_{4,K}$

In this section, we prove two propositions (Proposition 9.1 and Proposition 9.2) which establish the large *s* asymptotics of $I_{3,K}$ and $I_{4,K}$, respectively, where we henceforth choose $K = s^\rho$.

### Proposition 9.1

(Large *s* asymptotics of $I_{3,K}$). Let $\alpha > -1$ and let $K = s^\rho$. As $s \rightarrow +\infty$, the function $I_{3,K}$ defined in (6.12) satisfies

8.27e $$\begin{aligned} I_{3,K} = {\mathcal {I}}_{3}^{(3)} \ln (s^{\rho }) + I_{3}^{(3)} + {{{\mathcal {O}}}}(s^{-\rho }\ln (s^{\rho })) \end{aligned}$$

uniformly for $\theta$ in compact subsets of (0, 1], where the coefficients ${\mathcal {I}}_{3}^{(3)}$ and $I_{3}^{(3)}$ are given by

$$\begin{aligned} {\mathcal {I}}_{3}^{(3)} =&- \frac{3\alpha (1+\alpha -\theta )+\theta }{12\theta ^{2}(\theta +1)},\\ I_{3}^{(3)} =&\; \frac{3\alpha (1+\alpha -\theta )+\theta }{6\theta (\theta +1)^{2}} \ln ( \theta ) - \frac{3 + 3\alpha (4+3\alpha )-4\theta - 3\alpha \theta (4+\alpha ) + \theta ^{2}}{24 \theta ^{2}(1+\theta )}. \end{aligned}$$

### Proof

By the cyclicity of the trace, we can write the definition (6.12) of $I_{3,K}$ as

9.1 $$\begin{aligned} I_{3,K}&= \frac{1}{2} \int _{\sigma _{K}} H(\zeta ) {{\,\mathrm{Tr}\,}}\Big [R^{-1}(\zeta )R'(\zeta )e^{p_{0}\sigma _{3}}P^{\infty }(\zeta )\sigma _{3}P^{\infty }(\zeta )^{-1}e^{-p_{0}\sigma _{3}}\Big ] \frac{d\zeta }{2\pi i}, \end{aligned}$$

where $K = s^\rho$, and $\sigma _{K}$ and *H* are defined in Lemma 6.2. All the *s*-dependence of the trace lies in the factor $R^{-1}(\zeta )R'(\zeta )$, since by (2.18), the quantity

9.2 $$\begin{aligned} e^{p_{0}\sigma _{3}}P^{\infty }(\zeta )\sigma _{3}P^{\infty }(\zeta )^{-1}e^{-p_{0}\sigma _{3}} =&\; Q^{\infty }(\zeta ) \sigma _{3} Q^{\infty }(\zeta )^{-1} \nonumber \\ =&\; \frac{1}{r(\zeta )} \begin{pmatrix} \zeta -i\mathrm{Im}\,(b_2) &{} i\mathrm{Re}\,{b_2} \\ i\mathrm{Re}\,{b_2} &{} i\mathrm{Im}\,(b_2) - \zeta \end{pmatrix} \end{aligned}$$

is independent of *s*. As $s \rightarrow + \infty$, we have by Proposition 4.1 that

9.3 $$\begin{aligned}&R(\zeta )= I+ \frac{R^{(1)}(\zeta )}{s^\rho } + {{{\mathcal {O}}}}\bigg (\frac{1}{s^{2\rho }(1+ |\zeta |)}\bigg ) \end{aligned}$$

uniformly for $\zeta \in {\mathbb {C}}{\setminus } \Gamma _{R}$ and that this expansion can be differentiated with respect to $\zeta$. The asymptotics in (9.4) as well as all other asymptotic expansions in the rest of this section are uniform with respect to $\theta$ in compact subsets of (0, 1].

From the explicit expression (4.4) for $R^{(1)}$, we see that $R^{(1)}(\zeta )$ and $R^{(1)\prime }(\zeta )$ are ${{{\mathcal {O}}}}((1 +|\zeta |)^{-1})$ and ${{{\mathcal {O}}}}((1 +|\zeta |)^{-2})$, respectively, uniformly for $\zeta \in \sigma _K$ as $s \rightarrow +\infty$. Therefore,

$$\begin{aligned} R^{-1}(\zeta )R'(\zeta ) =&\; \bigg (I - \frac{R^{(1)}(\zeta )}{s^\rho } + {{{\mathcal {O}}}}\bigg (\frac{1}{s^{2\rho }(1+ |\zeta |)}\bigg )\bigg )\bigg (\frac{R^{(1)\prime }(\zeta )}{s^\rho } + {{{\mathcal {O}}}}\bigg (\frac{1}{s^{2\rho }(1+ |\zeta |)^2}\bigg )\bigg ) \\ =&\; \frac{R^{(1)\prime }(\zeta )}{s^\rho } + {\widetilde{R}}_{R}(\zeta ), \qquad \text{ as } s \rightarrow + \infty , \end{aligned}$$

uniformly for $\zeta \in \sigma _{K}$, where

$$\begin{aligned}&{\widetilde{R}}_{R}(\zeta ) = {{{\mathcal {O}}}}\bigg (\frac{1}{s^{2\rho }(1+ |\zeta |)^2}\bigg ) \qquad \text{ as } s \rightarrow + \infty , \end{aligned}$$

uniformly for $\zeta \in \sigma _{K}$. Hence, for large *s* and $\zeta \in \sigma _{K}$, we have

9.4 $$\begin{aligned}&{{\,\mathrm{Tr}\,}}\Big [R^{-1}(\zeta )R'(\zeta )e^{p_{0}\sigma _{3}}P^{\infty }(\zeta )\sigma _{3}P^{\infty }(\zeta )^{-1}e^{-p_{0}\sigma _{3}}\Big ]\nonumber \\&\quad = \frac{W(\zeta )}{s^\rho } + {{\,\mathrm{Tr}\,}}\Big [ {\widetilde{R}}_{R}(\zeta ) Q^{\infty }(\zeta ) \sigma _{3} Q^{\infty }(\zeta )^{-1} \Big ], \end{aligned}$$

where the function $W(\zeta )$ is defined by

9.5 $$\begin{aligned} W(\zeta ) = {{\,\mathrm{Tr}\,}}\bigg [R^{(1)\prime }(\zeta )Q^{\infty }(\zeta ) \sigma _{3} Q^{\infty }(\zeta )^{-1}\bigg ]. \end{aligned}$$

Substituting (9.5) into (9.2), we see that

9.6 $$\begin{aligned} I_{3,K} = I_{3} + {\widetilde{I}}_{3,K}, \end{aligned}$$

where $I_{3}$ and ${\widetilde{I}}_{3,K}$ are defined by

$$\begin{aligned} I_{3} =&\; \frac{1}{2s^{\rho }}\int _{\sigma } H(\zeta ) W(\zeta ) \frac{d\zeta }{2\pi i}, \\ {\widetilde{I}}_{3,K} =&\; \frac{1}{2}\int _{\sigma _{K}}H(\zeta ) {{\,\mathrm{Tr}\,}}\Big [ {\widetilde{R}}_{R}(\zeta ) Q^{\infty }(\zeta ) \sigma _{3} Q^{\infty }(\zeta )^{-1} \Big ] \frac{d\zeta }{2\pi i}. \end{aligned}$$

We first estimate ${\widetilde{I}}_{3,K}$. Since

9.7 $$\begin{aligned}&H(\zeta ) = {{{\mathcal {O}}}}\left( \frac{1}{s^{\rho }(\zeta -\zeta _{j})}\right) \qquad \text{ as } \zeta \rightarrow \zeta _{j}, \ j = 0, 1, \dots , \nonumber \\&H(\zeta ) = {{{\mathcal {O}}}}\big (\zeta s^{\rho } \ln (\zeta s^{\rho }) \big ) \qquad \text{ as } \zeta s^{\rho } \rightarrow \infty , \end{aligned}$$

we have (see also Fig. 8)

9.8 $$\begin{aligned} |{\widetilde{I}}_{3,K}| =&\; \bigg | \frac{1}{2}\int _{\sigma _{K}\cap \{|\zeta | \le c' s^{-\rho }\}}H(\zeta ) {{\,\mathrm{Tr}\,}}\Big [ {\widetilde{R}}_{R}(\zeta ) Q^{\infty }(\zeta ) \sigma _{3} Q^{\infty }(\zeta )^{-1} \Big ] \frac{d\zeta }{2\pi i} \nonumber \\&+ \frac{1}{2}\int _{\sigma _{K}\cap \{|\zeta |> c' s^{-\rho }\}}H(\zeta ) {{\,\mathrm{Tr}\,}}\Big [ {\widetilde{R}}_{R}(\zeta ) Q^{\infty }(\zeta ) \sigma _{3} Q^{\infty }(\zeta )^{-1} \Big ] \frac{d\zeta }{2\pi i} \bigg | \nonumber \\ \le&\; C' \int _{\sigma _{K}\cap \{|\zeta | \le c' s^{-\rho }\}} \frac{|d\zeta |}{s^{3\rho }|\zeta -\zeta _0|} + C' \int _{\sigma _{K}\cap \{|\zeta | > c' s^{-\rho }\}} \frac{\ln |\zeta s^{\rho }|}{|\zeta | s^{\rho }}|d\zeta | \nonumber \\ \le&\; \frac{C'}{s^{3\rho }} + C' \frac{\ln s}{s^{\rho }} = {{{\mathcal {O}}}}\left( \frac{\ln s}{s^{\rho }}\right) \end{aligned}$$

where $c',C'>0$ are two sufficiently large constants.

We next consider $I_{3}$. Substituting (4.4) and (9.3) into (9.6), it follows that

9.9 $$\begin{aligned} W(\zeta ) =&\; \frac{1}{r(\zeta )} {{\,\mathrm{Tr}\,}}\bigg [ \bigg (-\frac{A}{(\zeta -b_1)^2} - \frac{2B}{(\zeta -b_1)^3} + \frac{{\bar{A}}}{(\zeta -b_2)^2} - \frac{2 {\bar{B}}}{(\zeta -b_2)^3}\bigg ) \nonumber \\&\times \begin{pmatrix} \zeta -i\mathrm{Im}\,(b_2) &{} i\mathrm{Re}\,{b_2} \\ i\mathrm{Re}\,{b_2} &{} i\mathrm{Im}\,(b_2) - \zeta \end{pmatrix}\bigg ]. \end{aligned}$$

Replacing *r* by ${\tilde{r}}$ in *W* does not change the value of $I_{3}$. Deforming $\sigma$ (which surrounds 0) into another contour ${\tilde{\sigma }}$ which surrounds the cut $[b_{1},b_{2}]$ of ${\tilde{r}}$ once in the positive direction but which does not surround 0, it transpires that

$$\begin{aligned} I_{3} = \frac{1}{2s^{\rho }}\int _{{\tilde{\sigma }}} H(\zeta ) {\widetilde{W}}(\zeta ) \frac{d\zeta }{2\pi i}, \end{aligned}$$

where ${\widetilde{W}}$ is defined by the expression obtained by replacing *r* by ${\tilde{r}}$ in the right-hand side of (9.10). Assuming that ${\tilde{\sigma }}$ is bounded away from 0, we can replace $H(\zeta )$ by its large *s* asymptotics (8.18); this gives

$$\begin{aligned} I_{3} =&- \frac{1}{2} \int _{{\tilde{\sigma }}} \frac{i \zeta \ln \left( -\frac{i \zeta s^\rho }{\theta }\right) }{\theta ^2} {\widetilde{W}}(\zeta )\frac{d\zeta }{2\pi i} + {{{\mathcal {O}}}}(s^{-\rho }\ln (s^\rho )). \end{aligned}$$

We split the leading term as follows:

9.10 $$\begin{aligned} I_{3} = I_{3,1} + I_{3,2} + {{{\mathcal {O}}}}\big (s^{-\rho }\ln (s^\rho )\big ), \end{aligned}$$

where $I_{3,1}$ and $I_{3,2}$ are given by

$$\begin{aligned} I_{3,1} = - \frac{1}{2} \int _{{\tilde{\sigma }}} \frac{i \zeta \ln (-\frac{i s^\rho }{\theta })}{\theta ^2} {\widetilde{W}}(\zeta )\frac{d\zeta }{2\pi i}, \qquad I_{3,2} = - \frac{1}{2} \int _{{\tilde{\sigma }}} \frac{i \zeta \ln (\zeta )}{\theta ^2} {\widetilde{W}}(\zeta )\frac{d\zeta }{2\pi i}. \end{aligned}$$

From (9.10), we have the expansion

$$\begin{aligned} {\widetilde{W}}(\zeta ) = -\frac{1}{\zeta ^2} {{\,\mathrm{Tr}\,}}[(A - {\bar{A}})\sigma _3] + {{{\mathcal {O}}}}(\zeta ^{-3}) \qquad \text{ as } \zeta \rightarrow \infty . \end{aligned}$$

By deforming the contour ${\tilde{\sigma }}$ to infinity, we get

9.11 $$\begin{aligned} I_{3,1} = \frac{1}{2} \frac{i \ln (-\frac{i s^\rho }{\theta })}{\theta ^2} {{\,\mathrm{Tr}\,}}[(A - {\bar{A}})\sigma _3] = -\frac{(3 \alpha (1+\alpha -\theta )+\theta ) \ln (-\frac{i s^\rho }{\theta })}{12 \theta ^2 (\theta +1)}, \end{aligned}$$

while

$$\begin{aligned} I_{3,2} =&- \frac{1}{2} \lim _{R\rightarrow \infty }\bigg \{ \int _{C_{R}} \frac{i \zeta \ln (\zeta )}{\theta ^2} {\widetilde{W}}(\zeta )\frac{d\zeta }{2\pi i} + \int _{-R}^0 \frac{i \zeta }{\theta ^2} {\widetilde{W}}(\zeta ) d\zeta \bigg \}\\ =&- \frac{1}{2} \lim _{R\rightarrow \infty }\bigg \{-\frac{i }{\theta ^2} {{\,\mathrm{Tr}\,}}[(A - {\bar{A}})\sigma _3] \int _{C_{R}} \frac{\ln (\zeta )}{\zeta } \frac{d\zeta }{2\pi i} + \int _{-R}^0 \frac{i \zeta }{\theta ^2} W(\zeta ) d\zeta \bigg \}. \end{aligned}$$

We have

$$\begin{aligned} \int _{C_{R}} \frac{\ln (\zeta )}{\zeta } \frac{d\zeta }{2\pi i} = \ln {R} \end{aligned}$$

and the integral $\int _{-R}^0 \frac{i \zeta }{\theta ^2} W(\zeta ) d\zeta$ can be computed explicitly using (9.10). After simplification this gives

$$\begin{aligned} I_{3,2} = - \frac{3 + 3\alpha (4+3\alpha )-4\theta - 3\alpha \theta (4+\alpha ) + \theta ^{2}}{24 \theta ^{2}(1+\theta )} - \frac{3\alpha (1+\alpha - \theta )+\theta }{12 \theta ^{2}(1+\theta )}\ln \Big ( i \theta ^{\frac{1-\theta }{1+\theta }} \Big ). \end{aligned}$$

Substituting this expression for $I_{3,2}$ into (9.11) and recalling (9.7), (9.9), and (9.12), equation (9.1) follows. $\quad \square$

### Proposition 9.2

(Large *s* asymptotics of $I_{4,K}$). Let $\alpha > -1$ and let $K = s^\rho$. As $s \rightarrow +\infty$, the function $I_{4,K}$ defined in (6.13) satisfies, for any $N \ge 1$,

9.12 $$\begin{aligned} I_{4,K} = {{{\mathcal {O}}}}\big (s^{-N\rho }\big ) \end{aligned}$$

uniformly for $\theta$ in compact subsets of (0, 1].

### Proof

In view of (9.3), we can write

$$\begin{aligned} I_{4,K} = -\frac{1}{2} \int _{{\widetilde{\Sigma }}_{K}} H(\zeta ) {{\,\mathrm{Tr}\,}}\Big [\Big (R_{+}^{-1}(\zeta )R_{+}'(\zeta )-R_{-}^{-1}(\zeta )R_{-}'(\zeta )\Big )Q^{\infty }(\zeta ) \sigma _{3} Q^{\infty }(\zeta )^{-1}\Big ], \end{aligned}$$

where $Q^{\infty }(\zeta ) \sigma _{3} Q^{\infty }(\zeta )^{-1}$ is independent of *s* and ${{{\mathcal {O}}}}(1)$ as $\zeta \rightarrow \infty$. Using (4.3) and (9.8), we conclude that, for any *N* large enough,

$$\begin{aligned} |I_{4,K}| = {{{\mathcal {O}}}}\bigg (\int _{s^\rho }^\infty \frac{|\zeta | s^{\rho } \ln (|\zeta | s^{\rho })}{s^{N\rho }(1+ |\zeta |)^N} d|\zeta |\bigg ) = {{{\mathcal {O}}}}\big ( s^{-N\rho }\big ) \end{aligned}$$

uniformly for $\theta$ in compact subsets of (0, 1]. This proves (9.13). $\quad \square$

## Proof of Theorem 1.1

Substituting the large *s* asymptotics of the integrals $I_{1}$, $I_{2}$, $I_{3,K}$, and $I_{4,K}$ established in Sects. 7–9 (see Propositions 7.1, 8.1, 8.2, 8.3, 9.1, and 9.2 ) into the differential identity (6.9), we obtain

9.13 $$\begin{aligned} \partial _{\theta } \ln \det \Big ( \left. 1-{\mathbb {K}} \right| _{[0,s]} \Big )&= {\mathcal {I}}_{1}^{(1)} s^{2\rho }\ln (s^{\rho }) + I_{1}^{(1)}s^{2\rho } + ({\mathscr {X}}_{1}^{(2)}+{\mathscr {Z}}^{(2)})s^{\rho } \big ( \ln (s^{\rho })\big )^{2} \nonumber \\&\quad +\big ( {\mathcal {I}}_{1}^{(2)} + {\mathcal {X}}_{1}^{(2)}+{\mathcal {X}}_{3}^{(2)}+{\mathcal {Z}}^{(2)} \big ) s^{\rho }\ln (s^{\rho }) \nonumber \\&\quad + \big ( I_{1}^{(2)} + X_{1}^{(2)}+X_{3}^{(2)}+Z^{(2)} \big ) s^{\rho } \nonumber \\&\quad + \big ( {\mathcal {I}}_{1}^{(3)} + {\mathcal {X}}_{1}^{(3)} + {\mathcal {X}}_{3}^{(3)} + {\mathcal {Z}}^{(3)} + {\mathcal {I}}_{3}^{(3)} \big ) \ln (s^{\rho }) \nonumber \\&\quad + I_{1}^{(3)} + X_{1}^{(3)} + X_{2}^{(3)} + X_{3}^{(3)} + Z^{(3)} + I_{3}^{(3)} + {{{\mathcal {O}}}}\big ( s^{-\rho }\ln (s^{\rho })\big ) \end{aligned}$$

as $s \rightarrow + \infty$ uniformly for $\theta$ in compact subsets of (0, 1].

### Integration of the differential identity

Since the asymptotic formula (10.1) is valid uniformly for $\theta$ in compact subsets of (0, 1], we can integrate (10.1) with respect to $\theta$ from $\theta = 1$ to an arbitrary $\theta \in (0,1]$. Using the known result (1.7) valid for $\theta =1$, this yields the following lemma.

#### Lemma 10.1

Let $\alpha > -1$. The following expansion is valid uniformly for $\theta$ in compact subsets of (0, 1] as $s \rightarrow + \infty$:

10.1 $$\begin{aligned} \ln \det \Big ( \left. 1-{\mathbb {K}} \right| _{[0,s]} \Big ) =&-a s^{2\rho }+b s^{\rho }+c \ln s + \ln G(1+\alpha ) - \frac{\alpha }{2}\ln (2\pi ) - \frac{\alpha ^2}{2}\ln {2} \nonumber \\&- \int _{1}^{\theta } \ln G \left( 1+ \frac{1+\alpha }{2\theta '} \right) d\theta ' + \int _{1}^{\theta } W(\theta ', \alpha ) d\theta ' \nonumber \\&+ \int _{1}^{\theta } X_{2}^{(3)}(\theta ', \alpha )d\theta ' + {{{\mathcal {O}}}}\big (s^{-\rho } \ln (s^{\rho })\big ), \end{aligned}$$

where the coefficients *a*, *b*, *c* are given by (1.9) and (1.10), $X_{2}^{(3)} = X_{2}^{(3)}(\theta , \alpha )$ is given in (8.4), and $W(\theta , \alpha )$ is defined by

$$\begin{aligned} W(\theta , \alpha ) =&\; \frac{-3-12 \alpha - 6 \alpha ^{2} + 2 \theta + \theta ^{2}}{6(1+\theta )^{2}} \ln (\theta ) + \frac{1+\alpha }{4\theta }\ln (2\pi )\\&\quad + \frac{-3(1+\alpha )^{2} + (2+3\alpha )^{2} \theta - (1+6\alpha ) \theta ^{2}}{24 \theta ^{2} (1+\theta )} + \frac{1+6\alpha (1+\alpha )-\theta ^{2}}{12\theta ^{2}} \ln (1+\theta ) \\&\quad + \zeta '(-1). \end{aligned}$$

#### Proof

The proof involves long computations which use the definitions (2.4) and (2.7) of the constants $\{c_j\}_1^8$, $b_1$, and $b_2$, as well as the relations (7.15) satisfied by $\ln {b_1}$ and $\ln {b_2}$. Explicit expressions for the coefficients in (10.1) are given in Propositions 7.1, 8.1, 8.2, 8.3, 9.1, 9.2. After rather lengthy calculations, we find that the first six coefficients on the right-hand side of (10.1) can be expressed as

$$\begin{aligned}&{\mathcal {I}}_{1}^{(1)} = - \frac{2a}{\rho (1+\theta )^2}, \\&I_{1}^{(1)} = - \partial _\theta a, \\&{\mathscr {X}}_{1}^{(2)}+{\mathscr {Z}}^{(2)}= 0, \\&{\mathcal {I}}_{1}^{(2)} + {\mathcal {X}}_{1}^{(2)}+{\mathcal {X}}_{3}^{(2)}+{\mathcal {Z}}^{(2)} = \frac{b}{\rho (1+\theta )^2}, \\&I_{1}^{(2)} + X_{1}^{(2)}+X_{3}^{(2)}+Z^{(2)} = \partial _\theta b, \\&{\mathcal {I}}_{1}^{(3)} + {\mathcal {X}}_{1}^{(3)} + {\mathcal {X}}_{3}^{(3)} + {\mathcal {Z}}^{(3)} + {\mathcal {I}}_{3}^{(3)} = \frac{\partial _\theta c}{\rho }, \end{aligned}$$

where *a*, *b*, and *c* are given by (1.9) and (1.10). Integrating (10.1) from 1 to $\theta$ and using (1.7) to compute the boundary term at 1, this yields

$$\begin{aligned} \ln \det \Big ( \left. 1-{\mathbb {K}} \right| _{[0,s]} \Big ) =&-a s^{2\rho }+b s^{\rho }+c \ln s + \ln G(1+\alpha ) - \frac{\alpha }{2}\ln (2\pi )\\&+ \int _{1}^{\theta } \left. \Big ( I_{1}^{(3)} + X_{1}^{(3)} + X_{2}^{(3)} + X_{3}^{(3)} + Z^{(3)} + I_{3}^{(3)} \Big )\right| _{\theta '}d\theta ' \\&\quad + {{{\mathcal {O}}}}\big (s^{-\rho } \ln (s^{\rho })\big ) \end{aligned}$$

as $s \rightarrow +\infty$ uniformly for $\theta$ in compact subsets of (0, 1]. The lemma will follow if we can show that

$$\begin{aligned} I_{1}^{(3)} + X_{1}^{(3)} + X_{2}^{(3)} + X_{3}^{(3)} + Z^{(3)} + I_{3}^{(3)} = - \ln G \left( 1+ \frac{1+\alpha }{2\theta } \right) + W + X_{2}^{(3)}. \end{aligned}$$

This identity is a consequence of another long computation which also employs the identities

$$\begin{aligned} \ln (b_{1})&\; = \frac{1-\theta }{1+\theta } \ln (\theta ) + \ln (1+\theta ) + i(\pi -\phi ), \\ \ln (b_{2})&\; = \frac{1-\theta }{1+\theta } \ln (\theta ) + \ln (1+\theta ) + i\phi , \end{aligned}$$

which are a consequence of (2.7) and (2.8). $\quad \square$

#### Remark 10.2

Lemma 10.1 provides an alternative proof of the expressions (1.9) and (1.10) for *a*, *b*, and *c* based on the differential identity in $\theta$. Note that this method yields an error term in (10.2) of order ${{{\mathcal {O}}}}\big (s^{-\rho } \ln (s^{\rho })\big )$ for $\theta \le 1$ (and for $\theta \ge 1$ we would get ${{{\mathcal {O}}}}(s^{-\frac{1}{2}}\ln s)$), which is slightly worse than the optimal bound ${{{\mathcal {O}}}}(s^{-\rho })$ (which was proved via the differential identity in *s* in [12]).

To complete the proof of Theorem 1.1 it only remains to verify that the sum of the terms of order 1 on the right-hand side of (10.2) equals $\ln C$, where *C* is given by (1.11). In order to verify this we need to compute the three integrals on the right-hand side of (10.2). These integrals are computed in the following three lemmas.

#### Lemma 10.3

For $\alpha > -1$ and $\theta \in (0,1]$, it holds that

10.2 $$\begin{aligned}&-\int _{1}^{\theta } \ln G \Big (1 + \frac{1+\alpha }{2\theta '} \Big )d\theta ' = -\theta \ln G \left( 1+ \frac{1+\alpha }{2\theta } \right) + \ln G \left( 1+\frac{1+\alpha }{2} \right) \nonumber \\&\quad - \frac{1+\alpha }{2} \left( \frac{\ln (2\pi )-1}{2}\ln \theta - \frac{1+\alpha }{2} \frac{\theta -1}{\theta } - \ln \Gamma \left( 1+ \frac{1+\alpha }{2\theta } \right) + \ln \Gamma \left( \frac{3+\alpha }{2} \right) \right) . \end{aligned}$$

#### Proof

A simple integration by parts shows that

10.3 $$\begin{aligned}&\int _{1}^{\theta } \ln G \Big ( 1 + \frac{1+\alpha }{2\theta '} \Big )d\theta ' \nonumber \\&\quad = \bigg [\theta ' \ln G\Big (1+\frac{1+\alpha }{2\theta '} \Big ) \bigg ]_{\theta '=1}^\theta - \int _{1}^{\theta } \theta ' \partial _{\theta '} \left[ \ln G\left( 1+\frac{1+\alpha }{2 \theta '} \right) \right] d\theta '. \end{aligned}$$

Using the identities (see [27, Eq. 5.17.4] for the first identity)

$$\begin{aligned}&(\ln G)^{\prime }(z) = \frac{1}{2}(\ln (2\pi ) + 1) - z + (z-1)\psi (z) \quad \text{ and } \quad \partial _{\theta '}\left( 1+ \frac{1+\alpha }{2 \theta '} \right) = - \frac{1+\alpha }{2 \theta '^{2}}, \end{aligned}$$

we obtain

10.4 $$\begin{aligned}&- \int _{1}^{\theta } \theta ' \partial _{\theta '} \left[ \ln G\left( 1+\frac{1+\alpha }{2\theta '} \right) \right] d\theta ' \nonumber \\&\quad = \frac{1+\alpha }{2} \int _{1}^{\theta } \frac{1}{\theta '} \left[ \frac{\ln (2\pi )-1}{2} - \frac{1+\alpha }{2\theta '} + \frac{1+\alpha }{2\theta '} \psi \left( 1+ \frac{1+\alpha }{2\theta '} \right) \right] d\theta ' \nonumber \\&\quad = \frac{1+\alpha }{2} \left( \frac{\ln (2\pi )-1}{2}\ln \theta - \frac{1+\alpha }{2}\frac{\theta -1}{\theta }-\int _{1}^{\theta } \partial _{\theta '}\psi \left( 1+ \frac{1+\alpha }{2\theta '} \right) d\theta ' \right) \nonumber \\&\quad = \frac{1+\alpha }{2} \left( \frac{\ln (2\pi )-1}{2}\ln \theta - \frac{1+\alpha }{2}\frac{\theta -1}{\theta }-\left[ \psi \left( 1+ \frac{1+\alpha }{2\theta '} \right) \right] _{\theta ' = 1}^{\theta } \right) . \end{aligned}$$

Substituting (10.5) into (10.4) and simplifying, we find (10.3). $\quad \square$

#### Lemma 10.4

For $\alpha > -1$ and $\theta \in (0,1]$, it holds that

$$\begin{aligned}&\int _{1}^{\theta } W(\theta ', \alpha ) d\theta ' \\&\quad = \; \zeta '(-1) (\theta -1) + \left( \frac{1+\alpha }{4}\ln (2\pi ) + \frac{9+30\alpha +24\alpha ^{2}-3\theta -18 \alpha \theta + 4 \theta ^{2}}{24(1+\theta )} \right) \ln (\theta ) \\&\qquad + \frac{-1-6\alpha -6\alpha ^{2}+2\theta + 6 \alpha \theta - \theta ^{2}}{12\theta }\ln (1+\theta )-\frac{(\theta -1)\big ( 3(1+\alpha )^{2}+2\theta \big )}{24\theta } + \frac{\alpha ^{2}}{2} \ln 2. \end{aligned}$$

#### Proof

This follows from a long but straightforward computation. $\quad \square$

#### Lemma 10.5

For $\alpha > -1$ and $\theta \in (0,1]$, it holds that

10.5 $$\begin{aligned}&\int _{1}^{\theta } X_{2}^{(3)}(\theta ', \alpha ) d\theta ' \nonumber \\&\quad = \frac{\alpha }{2}\ln \theta - d(\theta ,\alpha ) + d(1,\alpha ) - (\theta - 1)\zeta '(-1) + \theta \ln G \left( 1 + \frac{1+\alpha }{2\theta } \right) \nonumber \\&\qquad - \ln G \left( \frac{3+\alpha }{2} \right) - \frac{1+\alpha }{2} \ln \Gamma \left( 1+\frac{1+\alpha }{2\theta } \right) +\frac{1+\alpha }{2}\ln \Gamma \left( 1+\frac{1+\alpha }{2} \right) \nonumber \\&\qquad - \frac{(\theta -1)\big ( 3(1+\alpha )^{2} - 2 \theta \big )}{24 \theta }. \end{aligned}$$

#### Proof

Integrating the definition (8.4) of $X_{2}^{(3)}$ from 1 to $\theta$ and appealing to Fubini’s theorem to interchange the order of integration and summation, we obtain

10.6 $$\begin{aligned} \int _{1}^{\theta } X_{2}^{(3)}(\theta ', \alpha ) d\theta ' =&\; \frac{\alpha }{2}\ln \theta + \lim _{N\rightarrow + \infty }\sum _{k=1}^{N} \Bigg \{ -\ln \Gamma (1+\alpha + k \theta ) + \ln \Gamma (1+\alpha + k) \nonumber \\&+ k (1-\theta ) + \left( \frac{1+\alpha }{2} + k \theta \right) \ln \left( \frac{1+\alpha }{2} + k \theta \right) \nonumber \\&- \left( \frac{1+\alpha }{2} + k \right) \ln \left( \frac{1+\alpha }{2} + k \right) \nonumber \\&- \frac{1-6\alpha - 9 \alpha ^{2}}{24 }\frac{1-\theta }{\theta } \ln \left( 1+\frac{1}{k} \right) + \frac{\alpha }{2} \ln \theta \Bigg \}. \end{aligned}$$

To simplify the sum in (10.7), we first consider the sum of $\ln \Gamma (1+\alpha + k)$. Using the reproducing formula for Barnes’ *G*-function (see [27, Eq. 5.17.1]),

$$\begin{aligned} G(z+1) = \Gamma (z) G(z), \end{aligned}$$

we can write

$$\begin{aligned} \sum _{k=1}^{N} \ln \Gamma (1+\alpha + k) = \ln G(2+\alpha +N)-\ln G (2+\alpha ). \end{aligned}$$

The asymptotics (7.10) of $\ln G$ then leads to the large *N* asymptotics

10.7 $$\begin{aligned} \sum _{k=1}^{N}&\ln \Gamma (1+\alpha + k) = \frac{N^{2}}{2}\ln N - \frac{3}{4} N^{2} + (1+\alpha ) N \ln N + \left( \frac{\ln (2\pi )}{2}-(1+\alpha ) \right) N \nonumber \\&+ \frac{5+12 \alpha + 6 \alpha ^{2}}{12} \ln N + \Big ( \zeta '(-1) + \frac{\ln (2\pi )}{2}(1+\alpha ) - \ln G(2+\alpha ) \Big ) + {{{\mathcal {O}}}}(N^{-1}). \end{aligned}$$

To simplify the terms in (10.7) involving $\ln (\frac{1+\alpha }{2} + k \theta )$, we utilize the Hurwitz zeta function $\zeta (u,z)$ which is defined for $\mathrm{Re}\,u > 1$ and $z \ne 0, -1, -2, \dots$ by

$$\begin{aligned} \zeta (u,z) = \sum _{n=0}^\infty \frac{1}{(n + z)^u}. \end{aligned}$$

We recall that this function, which generalizes Riemann’s zeta function $\zeta (u)$ in the sense that $\zeta (u,1) = \zeta (u)$, is defined for all $u \in {\mathbb {C}}{\setminus } \{1\}$ by analytic continuation. A simple shift of the summation index shows that

10.8 $$\begin{aligned} \zeta (u,z)-\zeta (u,z+N) = \sum _{n=0}^\infty \frac{1}{(n + z)^u} - \sum _{n=N+1}^\infty \frac{1}{(n + z)^u} = \sum _{n=0}^{N-1} \frac{1}{(n+z)^{u}}, \end{aligned}$$

whenever $\mathrm{Re}\,u > 1$. By analyticity, (10.9) is in fact valid for all $u \in {\mathbb {C}}{\setminus } \{1\}$ and $z \in {\mathbb {C}}{\setminus } \{0,-1, \dots \}$. Differentiating (10.9) with respect to *u* and evaluating the resulting equation at $u=-1$, we obtain

10.9 $$\begin{aligned} \zeta '(-1,z+N)-\zeta '(-1,z) = \sum _{n=0}^{N-1} (n+z)\ln (n+z), \end{aligned}$$

where $\zeta '(-1,z) := \left. \partial _{u} \zeta (u,z) \right| _{u=-1}$. It is a simple calculation to deduce from (10.10) that

$$\begin{aligned}&\sum _{k=1}^{N} \left( \frac{1+\alpha }{2} + k \theta \right) \ln \left( \frac{1+\alpha }{2} + k \theta \right) \\&\quad = \theta \bigg [ \zeta '\left( -1,1+N+\frac{1+\alpha }{2\theta }\right) - \zeta '\left( -1,1+\frac{1+\alpha }{2\theta } \right) \bigg ] \\&\qquad + \frac{\ln \theta }{2} N (1+\alpha + \theta + N \theta ). \end{aligned}$$

Using the asymptotic formula [27, Eq. 25.11.44]

$$\begin{aligned} \zeta '(-1,z) = \frac{z^{2}}{2}\ln z - \frac{z^{2}}{4} - \frac{z}{2}\ln z + \frac{1}{12} \ln z + \frac{1}{12} + {{{\mathcal {O}}}}(z^{-2}), \qquad z \rightarrow \infty , \end{aligned}$$

which is valid in the sector $|\arg z| < \frac{\pi }{2} - \delta$ for any fixed $\delta > 0$, we obtain, for any $\theta \in (0,1]$,

10.10 $$\begin{aligned}&\sum _{k=1}^{N}\left( \frac{1+\alpha }{2} + k \theta \right) \ln \left( \frac{1+\alpha }{2} + k \theta \right) = \frac{\theta }{2}N^{2} \ln N + \frac{\theta }{4}(2\ln \theta - 1) N^{2}\nonumber \\&\quad + \frac{1}{2}(1+\alpha +\theta ) N \ln N \nonumber \\&+ \frac{1}{2}(1+\alpha +\theta ) N \ln \theta + \frac{3(1+\alpha ^{2})+2\theta (3+\theta ) + 6 \alpha (1+\theta )}{24 \theta } \ln N\nonumber \\&+ \frac{3(1+\alpha ^{2})+2\theta (3+\theta ) + 6 \alpha (1+\theta )}{24 \theta } - \theta \zeta '\left( -1;\frac{1+\alpha + 2 \theta }{2 \theta }\right) + {{{\mathcal {O}}}}(N^{-1}) \end{aligned}$$

as $$N \rightarrow + \infty $$.

The asymptotics of the terms in (10.7) involving $\ln (\frac{1+\alpha }{2} + k)$ can be obtained by setting $\theta = 1$ in (10.11). Moreover, it is easy to check that

10.11 $$\begin{aligned}&\sum _{k=1}^{N} \bigg \{k(1-\theta ) + \frac{\alpha }{2} \ln \theta \bigg \} = \frac{1-\theta }{2}N^{2} + \frac{1-\theta }{2} N + \frac{\alpha }{2}N \ln \theta \end{aligned}$$

and

10.12 $$\begin{aligned} - \sum _{k=1}^{N} \frac{1-6\alpha - 9 \alpha ^{2}}{24 }\frac{1-\theta }{\theta } \ln \left( 1+\frac{1}{k} \right) = - \frac{1-6\alpha - 9 \alpha ^{2}}{24 }\frac{1-\theta }{\theta } \ln (N+1). \end{aligned}$$

Substituting (10.8), (10.11), (10.12), and (10.13) into (10.7) and using that $\ln (N+1)$ can be replaced by $\ln N$ because $\ln (N+1) - \ln {N} \rightarrow 0$ as $N \rightarrow +\infty$, we obtain

$$\begin{aligned}&\int _{1}^{\theta } X_{2}^{\infty }(c) d\theta = \frac{\alpha }{2}\ln \theta + \lim _{N \rightarrow + \infty } \Bigg \{ \bigg ( -\sum _{k=1}^{N} \ln \Gamma (1+\alpha + k \theta ) \bigg ) + \frac{\theta }{2} N^{2} \ln N + (2 \ln \theta - 3)\frac{\theta }{4}N^{2} \\&\quad +\left( 1+\alpha + \frac{\theta -1}{2}\right) N \ln N + \left( \frac{\ln (2\pi )}{2}-(1+\alpha )+\frac{1-\theta }{2} + \left( \alpha + \frac{1+\theta }{2} \right) \ln \theta \right) N \\&\quad +\frac{1+6\alpha ^{2} + \theta (3+\theta ) + 6\alpha (1+\theta )}{12 \theta } \ln N + \zeta '(-1) - \theta \zeta '\left( -1;\frac{1+\alpha + 2 \theta }{2 \theta } \right) + \zeta ' \left( -1;\frac{3+\alpha }{2} \right) \\&\quad +\frac{\ln (2\pi )}{2}(1+\alpha ) - \ln G(2+\alpha ) + \frac{3(1+\alpha ^{2})+2\theta (3+\theta ) + 6 \alpha (1+\theta )}{24 \theta } - \frac{11+12\alpha + 3 \alpha ^{2}}{24} \Bigg \}, \end{aligned}$$

which, recalling the definition (1.12) of the quantity $d(\theta ,\alpha )$, can be rewritten as

10.13 $$\begin{aligned} \int _{1}^{\theta } X_{2}^{(3)}(\theta ', \alpha ) d\theta ' =&\; \frac{\alpha }{2}\ln \theta - d(\theta ,\alpha ) + d(1,\alpha ) - \theta \zeta '\left( -1;\frac{1+\alpha + 2 \theta }{2 \theta } \right) + \zeta ' \left( -1;\frac{3+\alpha }{2} \right) \nonumber \\&- \frac{(\theta -1)\big ( 3(1+\alpha )^{2} - 2 \theta \big )}{24 \theta }. \end{aligned}$$

Using the following identity which relates the Barnes *G*-function to $\zeta '(-1,z)$ (see [2, Eq. (18)]):

$$\begin{aligned} \ln G(z) = \zeta '(-1)-\zeta '(-1,z) + (z-1) \ln \Gamma (z), \end{aligned}$$

we can rewrite (10.14) as (10.6). $\quad \square$

#### Remark 10.6

Note that the reasoning leading to (10.8) cannot be applied to the sum

$$\begin{aligned} \sum _{k=1}^{N} \ln \Gamma (1+\alpha + k \theta ) \end{aligned}$$

for general values of $\theta$. In fact, this is the only finite *N* sum in (10.7) which we are not able to evaluate in terms of known special functions.

Replacing the three integrals on the right-hand side of (10.2) with the expressions derived in Lemmas 10.3–10.5, we obtain the following expression for the term of order 1 in the large *s* asymptotics of $\ln \det ( \left. 1-{\mathbb {K}} \right| _{[0,s]} )$:

$$\begin{aligned}&\ln G ( 1+\alpha ) - \frac{\alpha }{2} \ln (2\pi ) + d(1,\alpha ) - d(\theta ,\alpha ) \\&\quad + \frac{24 \alpha (\alpha +2)+15+3\theta + 4 \theta ^{2}}{24(1+\theta )} \ln \theta + \frac{6\alpha \theta - 6 \alpha (1+\alpha )-(\theta -1)^{2}}{12 \theta } \ln (1+\theta ), \end{aligned}$$

which is precisely $\ln C$, where *C* is defined by (1.11). This finishes the proof in the case when $\theta \in (0,1]$; as explained in Sect. 1.2.1 the result for $\theta \in [1, \infty )$ then follows by symmetry. The proof of Theorem 1.1 is therefore complete.

## Proof of Proposition 1.4

In this “Appendix”, we establish the formula (1.17) for $d(\theta , \alpha )$ for rational values of $\theta$ stated in Proposition 1.4.

Let $\theta = p/q$ where $p,q \ge 1$ are two (not necessarily relatively prime) integers. Let $N = mq$ where $m \ge 1$ is an integer (later we will take $m \rightarrow + \infty$). We have

10.14 $$\begin{aligned} \prod _{k=1}^{N} \Gamma (1+\alpha + k \theta ) = \prod _{k=1}^{mq} \Gamma \left( 1+\alpha + \frac{kp}{q}\right) = \prod _{k=1}^{q} \prod _{j=0}^{m-1} \Gamma \left( 1+\alpha + jp + k\frac{p}{q} \right) . \end{aligned}$$

We recall that $\Gamma (z)$ satisfies the duplication formula (see [27, Eq. 5.5.6])

A.1 $$\begin{aligned} \Gamma (pz) = p^{pz-\frac{1}{2}}(2\pi )^{\frac{1-p}{2}} \prod _{\ell = 0}^{p-1} \Gamma \left( z + \frac{\ell }{p} \right) . \end{aligned}$$

Evaluating (A.2) at $z = \frac{1+\alpha }{p}+j + \frac{k}{q}$, we find

A.2 $$\begin{aligned} \Gamma \left( 1+\alpha + jp + k\frac{p}{q} \right) = p^{\frac{1}{2}+\alpha + jp + k\frac{p}{q}}(2\pi )^{\frac{1-p}{2}} \prod _{\ell = 0}^{p-1} \Gamma \left( \frac{1+\alpha }{p}+j + \frac{k}{q} + \frac{\ell }{p} \right) . \end{aligned}$$

Substituting (A.3) into (A.1), we obtain

A.3 $$\begin{aligned} \prod _{k=1}^{N} \Gamma (1+\alpha + k \theta )&= \prod _{k=1}^{q} \prod _{j=0}^{m-1} p^{\frac{1}{2}+\alpha + jp + k\frac{p}{q}}(2\pi )^{\frac{1-p}{2}} \nonumber \\&\times \prod _{k=1}^{q} \prod _{\ell =0}^{p-1} \prod _{j=0}^{m-1} \Gamma \left( \frac{1+\alpha }{p}+j + \frac{k}{q} + \frac{\ell }{p} \right) . \end{aligned}$$

The last product can be expressed in terms of Barnes’ *G*-function:

$$\begin{aligned} \prod _{j=0}^{m-1} \Gamma \left( \frac{1+\alpha }{p}+j + \frac{k}{q} + \frac{\ell }{p} \right) = \frac{G \left( \frac{1+\alpha }{p} + m + \frac{k}{q} + \frac{\ell }{p} \right) }{G \left( \frac{1+\alpha }{p} + \frac{k}{q} + \frac{\ell }{p} \right) }. \end{aligned}$$

On the other hand, we have

$$\begin{aligned} \prod _{j=0}^{m-1} p^{\frac{1}{2}+\alpha + jp + k\frac{p}{q}}(2\pi )^{\frac{1-p}{2}} = p^{m \left( \frac{1}{2} + \alpha + k \frac{p}{q} \right) }(2\pi )^{\frac{(1-p)m}{2}} p^{\frac{pm(m-1)}{2}}. \end{aligned}$$

Therefore, taking the logarithm, we can write equation (A.4) as

$$\begin{aligned}&\sum _{k=1}^{N} \ln \Gamma (1+\alpha + k \theta ) \\&\quad = \sum _{k=1}^{q} \left\{ \left( \frac{1}{2} + \alpha + k \frac{p}{q} \right) m \ln p + \frac{(1-p)m}{2} \ln (2\pi ) + \frac{pm(m-1)}{2} \ln p \right\} \\&\quad + \sum _{k=1}^{q} \sum _{\ell = 0}^{p-1} \left\{ \ln G \left( \frac{1+\alpha }{p} + m + \frac{k}{q} + \frac{\ell }{p} \right) - \ln G \left( \frac{1+\alpha }{p} + \frac{k}{q} + \frac{\ell }{p} \right) \right\} . \end{aligned}$$

As $m \rightarrow + \infty$, by definition of *d*, the term of order 1 in the above expression is given by

$$\begin{aligned}&d\Big (\theta = \frac{p}{q},\alpha \Big ) +\frac{1+6\alpha ^{2} + \theta (3+\theta ) + 6\alpha (1+\theta )}{12 \theta } \ln q \\&\quad = \sum _{k=1}^{q} \sum _{\ell = 0}^{p-1} \left\{ \zeta '(-1) + \left( \frac{k}{2q} + \frac{1+\alpha + \ell -p}{2p} \right) \ln (2\pi ) - \ln G \left( \frac{1+\alpha }{p} + \frac{k}{q} + \frac{\ell }{p} \right) \right\} , \end{aligned}$$

where we have used that the term of order 1 in the large *m* expansion of $\ln G \left( \frac{1+\alpha }{p} + m + \frac{k}{q} + \frac{\ell }{p} \right)$ is given by

A.4 $$\begin{aligned} \zeta '(-1) + \left( \frac{k}{2q} + \frac{1+\alpha + \ell -p}{2p} \right) \ln (2\pi ). \end{aligned}$$

Simplifying the double sum, we arrive at the following expression for *d*:

$$\begin{aligned} d\Big (\theta = \frac{p}{q},\alpha \Big )&= pq\zeta '(-1) + \left( \frac{p(q+1)}{4} + \frac{(1+\alpha )q}{2} -\frac{1}{2} pq + \frac{q(p-1)}{4} \right) \ln (2\pi ) \\&\quad - \sum _{k=1}^{q} \sum _{\ell = 0}^{p-1} \ln G \left( \frac{1+\alpha }{p} + \frac{k}{q} + \frac{\ell }{p} \right) - \frac{1+6\alpha ^{2} + \theta (3+\theta ) + 6\alpha (1+\theta )}{12 \theta } \ln q \\&= pq\zeta '(-1) + \left( \frac{(1+\alpha )q}{2} + \frac{p-q}{4} \right) \ln (2\pi ) - \sum _{k=1}^{q} \sum _{\ell = 0}^{p-1} \ln G \left( \frac{1+\alpha }{p} + \frac{k}{q} + \frac{\ell }{p} \right) . \end{aligned}$$

After some simple cancellations and a simple change of indices, we obtain (1.17).

## Proof of Proposition 1.6

In this “Appendix”, we prove the symmetry relation (1.20) for *d* given in Proposition 1.6. We first use (1.17) to prove the relation for rational values of $\theta$. We then use continuity to extend it to all $\theta > 0$.

Let $\theta = p/q$ for some $p,q \in {\mathbb {N}}{\setminus } \{ 0 \}$. From (1.17), we have

A.5 $$\begin{aligned} d(\theta ,\alpha )-d \left( \frac{1}{\theta },\frac{1+\alpha }{\theta }-1 \right) =&\; \frac{p-q}{2} \ln (2\pi )+\frac{\alpha -\theta \alpha +1-\theta }{ \theta } \ln p\nonumber \\&+ \frac{1+6\alpha ^{2} + \theta (3+\theta ) + 6\alpha (1+\theta )}{12 \theta } \ln \theta + D(p,q, \alpha ), \end{aligned}$$

where the function $D(p,q, \alpha )$ is defined by

$$\begin{aligned} D(p,q, \alpha ) = - \sum _{k=1}^{q} \sum _{\ell = 1}^{p} \ln G \left( \frac{\ell +\alpha }{p} + \frac{k}{q} \right) + \sum _{k=1}^{q} \sum _{\ell = 1}^{p} \ln G \left( \frac{k-1 }{q} + \frac{\ell +1+\alpha }{p} \right) . \end{aligned}$$

Simplification gives

$$\begin{aligned} D(p,q, \alpha ) =&- \sum _{k=1}^{q} \sum _{\ell = 1}^{p} \ln G \left( \frac{\ell +\alpha }{p} + \frac{k}{q} \right) + \sum _{k=0}^{q-1} \sum _{\ell = 2}^{p+1} \ln G \left( \frac{k}{q} + \frac{\ell +\alpha }{p} \right) \\ =&-\sum _{\ell = 1}^{p} \ln G \left( \frac{\ell +\alpha }{p} + 1 \right) - \sum _{k=1}^{q} \ln G \left( \frac{1+\alpha }{p} + \frac{k}{q} \right) \\&+\sum _{\ell = 2}^{p+1} \ln G \left( \frac{\ell +\alpha }{p} \right) +\sum _{k=0}^{q-1}\ln G \left( \frac{k}{q} + \frac{1+\alpha }{p}+1 \right) \\ =&\; \sum _{k=1}^{q-1} \bigg \{ \ln G \left( \frac{k}{q} + \frac{1+\alpha }{p}+1 \right) -\ln G \left( \frac{1+\alpha }{p} + \frac{k}{q} \right) \bigg \}\\&+\sum _{\ell = 1}^{p-1}\bigg \{ \ln G \left( \frac{\ell +1+\alpha }{p} \right) -\ln G \left( \frac{\ell +1+\alpha }{p} + 1 \right) \bigg \}. \end{aligned}$$

Using the identity $G(z+1)=\Gamma (z)G(z)$ and the duplication formula for $\Gamma$ (see (A.2)), we obtain

$$\begin{aligned} D(p,q, \alpha ) =&\;\sum _{k=1}^{q-1} \ln \Gamma \left( \frac{1+\alpha }{p} + \frac{k}{q} \right) -\sum _{\ell = 1}^{p-1} \ln \Gamma \left( \frac{1+\alpha }{p} +\frac{\ell }{p} \right) \\ =&\; \sum _{k=0}^{q-1} \ln \Gamma \left( \frac{1+\alpha }{p} + \frac{k}{q} \right) -\sum _{\ell = 0}^{p-1} \ln \Gamma \left( \frac{1+\alpha }{p}+\frac{\ell }{p} \right) \\ =&\; \ln \Gamma \left( \frac{1+\alpha }{\theta } \right) - \left( \frac{1+\alpha }{\theta }-\frac{1}{2} \right) \ln q -\frac{1-q}{2}\ln (2\pi )- \ln \Gamma \left( 1+\alpha \right) \\&+ \left( \frac{1}{2}+\alpha \right) \ln p+ \frac{1-p}{2} \ln (2\pi ). \end{aligned}$$

Substituting this expression for *D* into (B.1), we arrive at

$$\begin{aligned} d(\theta ,\alpha )-d \left( \frac{1}{\theta },\frac{1+\alpha }{\theta }-1 \right) =&\; \ln \Gamma \left( \frac{1+\alpha }{\theta } \right) - \ln \Gamma \left( 1+\alpha \right) +\left( \frac{1+\alpha }{\theta }-\frac{1}{2} \right) \ln \theta \\&+ \frac{1+6\alpha ^{2} + \theta (3+\theta ) + 6\alpha (1+\theta )}{12 \theta } \ln \theta \\ =&\; \ln \Gamma \left( \frac{1+\alpha }{\theta } \right) - \ln \Gamma \left( 1+\alpha \right) \\&+ \frac{13 + 6 \alpha ^{2} + \theta (\theta -3) + 6 \alpha (\theta + 3)}{12 \theta }\ln \theta , \end{aligned}$$

which proves (1.20) for rational values of $\theta$.

The definition (1.12) of $d(\theta , \alpha )$ can be written as

B.1 $$\begin{aligned} d(\theta ,\alpha ) = \lim _{N\rightarrow + \infty } d_N(\theta ,\alpha ), \end{aligned}$$

where the functions $d_N$ are defined by

$$\begin{aligned} d_N(\theta ,\alpha ) =&\; \sum _{k=1}^{N} \ln \Gamma (1+\alpha + k \theta ) - \Bigg \{\frac{\theta }{2} N^{2} \ln N + \frac{\theta (2 \ln \theta - 3)}{4}N^{2} \\&+\left( 1+\alpha + \frac{\theta -1}{2}\right) N \ln N + \left( \frac{\ln (2\pi )}{2}-(1+\alpha )+\frac{1-\theta }{2} + \left( \alpha + \frac{1+\theta }{2} \right) \ln \theta \right) N \nonumber \\&+\frac{1+6\alpha ^{2} + \theta (3+\theta ) + 6\alpha (1+\theta )}{12 \theta } \ln N \Bigg \}. \end{aligned}$$

The proof of Proposition 1.6 will be complete if we can show that the convergence in (B.2) is uniform for $(\theta ,\alpha )$ in compact subsets of ${\mathcal {U}}$, where

$$\begin{aligned} {\mathcal {U}} = ({\mathbb {C}}{\setminus } [0,-\infty )) \times \{ \alpha \in {\mathbb {C}}: \mathrm{Re}\,\alpha >-1\}. \end{aligned}$$

Indeed, if this is the case, then since each function $d_N$ is holomorphic ${\mathcal {U}} \rightarrow {\mathbb {C}}$, so is *d*; thus (1.20) must hold also for irrational values of $\theta > 0$ by continuity.

Let $K \subset {\mathcal {U}}$ be compact. By (3.5), we have

$$\begin{aligned} \sum _{k=1}^{N} \ln \Gamma (1+\alpha + k \theta ) =&\; \sum _{k=1}^{N}\bigg [ (1+\alpha + k \theta ) \ln (1+\alpha + k \theta )- (1+\alpha + k \theta ) -\frac{1}{2} \ln (1+\alpha + k \theta )\\&+\frac{1}{2} \ln (2\pi )+\frac{1}{12(1+\alpha + k \theta )}\bigg ] + \sum _{k=1}^{N} {\mathcal {D}}_1(1+\alpha + k \theta ), \end{aligned}$$

where ${\mathcal {D}}_1$ is the remainder defined in (3.6). Using the relation (10.9), we obtain

B.2 $$\begin{aligned}&\sum _{k=1}^{N}\bigg [ (1+\alpha + k \theta ) \ln (1+\alpha + k \theta )- (1+\alpha + k \theta ) -\frac{1}{2} \ln (1+\alpha + k \theta )\nonumber \\&\qquad +\frac{1}{2} \ln (2\pi )+\frac{1}{12(1+\alpha + k \theta )}\bigg ] \nonumber \\&\quad = \; \theta \bigg ( \zeta '\bigg ( -1,1+N+\frac{1+\alpha }{\theta } \bigg )- \zeta '\bigg ( -1,1+\frac{1+\alpha }{\theta } \bigg )\bigg ) + \frac{\ln \theta }{2} N(2+2\alpha +\theta + N\theta ) \nonumber \\&\qquad - N(1+\alpha )+\theta \frac{N^2 +N-2}{2}- \frac{1}{2} N \ln \theta -\frac{1}{2} \bigg ( \ln \Gamma \bigg ( 1+\frac{1+\alpha }{\theta }+N \bigg ) - \ln \Gamma \bigg ( 1+\frac{1+\alpha }{\theta } \bigg )\bigg )\nonumber \\&\qquad +\frac{N}{2} \ln (2\pi )+ \frac{1}{12 \theta } \bigg ( \psi \bigg ( 1+\frac{1+\alpha }{\theta }+N \bigg ) - \psi \bigg ( 1+\frac{1+\alpha }{\theta } \bigg )\bigg ). \end{aligned}$$

All the special functions on the right-hand side of (B.3) have uniform expansions for large *N* whenever the argument of

$$\begin{aligned} 1+\frac{1+\alpha }{\theta }+N \end{aligned}$$

is bounded away from $\pm \pi$; in particular, this is the case for $(\theta ,\alpha )\in K$. Furthermore, by (3.7), there are constants $C',C''>0$ (that only depend on *K*) such that

$$\begin{aligned} |{\mathcal {D}}_1(1+\alpha +k\theta )| \le \frac{C'}{(1+\alpha + k\theta )^3} \le \frac{C''}{k^3} \end{aligned}$$

and thus the series $\sum _{k=1}^{\infty } {\mathcal {D}}_1(1+\alpha +k\theta )$ converges uniformly for $(\theta ,\alpha )\in K$. We conclude that the sequence of functions $d_N$ converges to *d* uniformly for $(\theta ,\alpha )$ in compact subsets of ${\mathcal {U}}$ and thus the proof of Proposition 1.6 is complete.

## Proof of Lemma 7.2

Assume $\alpha > -1$ and $\theta \in (0,1]$, so that $b_1$ and $b_2$ lie in the second and first quadrants, respectively. For any integer *j*, a contour deformation shows that

B.3 $$\begin{aligned} 2\int _{\gamma _{b_2b_1}} \frac{\zeta ^j}{r(\zeta )} \frac{d\zeta }{2\pi i} = 2\int _{\gamma _{b_2b_1}} \frac{\zeta ^j}{{\tilde{r}}(\zeta )} \frac{d\zeta }{2\pi i} = \int _{\sigma } \frac{\zeta ^j}{{\tilde{r}}(\zeta )} \frac{d\zeta }{2\pi i}, \end{aligned}$$

where $\sigma$ is a closed loop surrounding once $\Sigma _{5}$ in the positive direction but not surrounding 0, and where we recall that the square roots *r* and ${\tilde{r}}$ defined in (2.9) and (7.4) have branch cuts along $\Sigma _{5}$ and $[b_{1},b_{2}]$, respectively. If $j \ge 0$, then $\frac{\zeta ^j}{{\tilde{r}}(\zeta )}$ is analytic in ${\mathbb {C}}{\setminus } [b_{1},b_{2}]$, and then deforming the contour $\sigma$ to infinity, we see that the right-hand side of (C.1) equals the coefficient of $\zeta ^{-1}$ in the large $\zeta$ expansion of $\frac{\zeta ^j}{r(\zeta )}$. Since

$$\begin{aligned} \frac{1}{r(\zeta )} = \frac{1}{\zeta } + \frac{b_1 + b_2}{2\zeta ^2} + \frac{3 b_1^2+2 b_1 b_2+3 b_2^2}{8 \zeta ^3 } + {{{\mathcal {O}}}}(\zeta ^{-4}), \qquad \zeta \rightarrow \infty , \end{aligned}$$

this proves the first three identities (7.14a)–(7.14c) of the lemma.

On the other hand, if $j \le -1$ in (C.1), then $\frac{\zeta ^{j}}{{\tilde{r}}(\zeta )}$ has no residue at $\infty$ but has a pole of order |*j*| at 0. By deforming the contour $\sigma$ through $\infty$, we obtain

$$\begin{aligned} 2 \int _{\gamma _{b_{2}b_{1}}} \frac{\zeta ^{j}}{ r(\zeta )}\frac{d\zeta }{2\pi i} = - \int _{C_{\epsilon }} \frac{\zeta ^{j}}{ {\tilde{r}}(\zeta )}\frac{d\zeta }{2\pi i}, \end{aligned}$$

where $C_{\epsilon }$ denotes a small circle of radius $\epsilon$ centered at 0 oriented positively. Therefore the right-hand side of (C.1) is equal to the coefficient of $\zeta ^{-1}$ in the expansion of $\frac{\zeta ^{j}}{{\tilde{r}}(\zeta )}$ as $\zeta \rightarrow 0$. Since

$$\begin{aligned} \frac{1}{{\tilde{r}}(\zeta )} = \frac{i}{|b_{2}|} + {{{\mathcal {O}}}}(\zeta ) \qquad \text{ as } \zeta \rightarrow 0, \end{aligned}$$

this proves (7.14d).

To prove the remaining four identities, we note that the same kind of argument that gave (C.1), shows that, for any $j \in {\mathbb {Z}}$,

C.1 $$\begin{aligned} 2\int _{\gamma _{b_2b_1}} \frac{\zeta ^j \ln {\zeta }}{r(\zeta )} \frac{d\zeta }{2\pi i} =&\; 2\int _{\gamma _{b_2b_1}} \frac{\zeta ^j \ln {\zeta }}{{\tilde{r}}(\zeta )} \frac{d\zeta }{2\pi i} = \int _{\sigma } \frac{\zeta ^j \ln {\zeta }}{{\tilde{r}}(\zeta )} \frac{d\zeta }{2\pi i}. \end{aligned}$$

If $j \ge 0$, then by deforming $\sigma$ to $C_{R} \cup \big ((-R,0) + i 0^{+}\big ) \cup \big ((0,-R) - i 0^{+}\big )$ where $R > 0$ is any large radius, and noting that $r = {\tilde{r}}$ over the range of integration, we get

$$\begin{aligned} 2\int _{\gamma _{b_2b_1}} \frac{\zeta ^j \ln {\zeta }}{r(\zeta )} \frac{d\zeta }{2\pi i} =&\; \int _{C_{R}} \frac{\zeta ^j \ln {\zeta }}{r(\zeta )} \frac{d\zeta }{2\pi i} + \int _{-R}^0 \frac{\zeta ^j 2\pi i}{r(\zeta )} \frac{d\zeta }{2\pi i}. \end{aligned}$$

Since the left-hand side is independent of *R*, by taking the limit $R \rightarrow + \infty$, we obtain

C.2 $$\begin{aligned}&2\int _{\gamma _{b_2b_1}} \frac{\zeta ^j \ln {\zeta }}{r(\zeta )} \frac{d\zeta }{2\pi i} \nonumber \\&=\lim _{R\rightarrow + \infty } \bigg \{\int _{-\pi }^{\pi } R^je^{i j \varphi } \ln (Re^{i\varphi })\bigg (\frac{1}{Re^{i\varphi }} + \frac{b_1 + b_2}{2 R^2 e^{2i \varphi }} + {{{\mathcal {O}}}}(R^{-3})\bigg ) \frac{iRe^{i\varphi } d\varphi }{2\pi i} + \int _{-R}^0 \frac{\zeta ^j 2\pi i}{r(\zeta )} \frac{d\zeta }{2\pi i} \bigg \}. \end{aligned}$$

Taking $j = 0$ in (C.3), we find

$$\begin{aligned} 2\int _{\gamma _{b_2b_1}} \frac{\ln {\zeta }}{r(\zeta )} \frac{d\zeta }{2\pi i} =&\lim _{R \rightarrow \infty } \bigg \{\int _{-\pi }^{\pi } \ln (Re^{i\varphi })\bigg (\frac{1}{Re^{i\varphi }} + \frac{b_1 + b_2}{2 R^2 e^{2i \varphi }} \bigg ) \frac{iRe^{i\varphi } d\varphi }{2\pi i} + \int _{-R}^0 \frac{1}{r(\zeta )} d\zeta \bigg \}\\ =&\lim _{R \rightarrow \infty } \bigg \{\ln {R} + \int _{-R}^0 \frac{1}{r(\zeta )} d\zeta \bigg \}. \end{aligned}$$

Using that

$$\begin{aligned} \frac{d}{d\zeta } \ln \big (-2 r(\zeta ) (1 + r'(\zeta ))\big ) = \frac{1}{r(\zeta )}, \end{aligned}$$

we can compute the large *R* asymptotics of the integral from $-R$ to 0:

$$\begin{aligned}&\int _{-R}^0 \frac{1}{r(\zeta )} d\zeta = \bigg [\ln \big (-2 r(\zeta ) (1 + r'(\zeta ))\big )\bigg ]_{\zeta = -R}^0 \\&\quad = -\ln (R) + \ln (i(|b_2| + \mathrm{Im}\,{b_2})) - \ln {2} + {{{\mathcal {O}}}}(R^{-1}). \end{aligned}$$

This yields (7.14e).

Taking $j = 1$ in (C.3), we find

$$\begin{aligned}&2\int _{\gamma _{b_2b_1}} \frac{\zeta \ln {\zeta }}{r(\zeta )} \frac{d\zeta }{2\pi i} \\&\quad = \lim _{R \rightarrow \infty } \bigg \{\int _{-\pi }^{\pi } Re^{i \varphi } \ln (Re^{i\varphi })\bigg (\frac{1}{Re^{i\varphi }} + \frac{b_1 + b_2}{2 R^2 e^{2i \varphi }} \bigg ) \frac{iRe^{i\varphi } d\varphi }{2\pi i} + \int _{-R}^0 \frac{\zeta }{r(\zeta )} d\zeta \bigg \}. \end{aligned}$$

Using that

$$\begin{aligned} \frac{d}{d\zeta } \bigg \{r(\zeta ) + \frac{b_1 + b_2}{2} \ln (-2 r(\zeta ) (1 + r'(\zeta )))\bigg \} = \frac{\zeta }{r(\zeta )}, \end{aligned}$$

we obtain the following large *R* asymptotics:

$$\begin{aligned}&\int _{-R}^0 \frac{\zeta }{r(\zeta )} d\zeta = \bigg [r(\zeta ) + \frac{b_1 + b_2}{2} \ln [-2 r(\zeta ) (1 + r'(\zeta ))]\bigg ]_{\zeta = -R}^0 \\&\quad = r_{-}(0) + \frac{b_1 + b_2}{2} \ln [-2 r_{-}(0) (1 + r_{-}'(0))] - \bigg (r(-R) + \frac{b_1 + b_2}{2} \ln [-2 r(-R) (1 + r'(-R))]\bigg ) \\&\quad = -i|b_2| + i \mathrm{Im}\,(b_2)\ln (2i(|b_2| + \mathrm{Im}\,b_2)) \\&\qquad - \bigg (-\sqrt{(R+b_1)(R+b_2)} + \frac{b_1 + b_2}{2} \ln [2 \sqrt{(R+b_1)(R+b_2)} + b_1 + b_2 + 2R]\bigg ) \\&\quad = R - i\mathrm{Im}\,(b_2) \ln R -i |b_2| + i\Big (1 + \ln (2i(|b_2| + \mathrm{Im}\,b_2)) - \ln 4\Big )\mathrm{Im}\,{b_2} + {{{\mathcal {O}}}}(R^{-1}), \end{aligned}$$

where we have fixed the branch of $\sqrt{(R+b_1)(R+b_2)}$ so that $\sqrt{(R+b_1)(R+b_2)} \sim R$ as $R \rightarrow +\infty$. Since

$$\begin{aligned}&\int _{-\pi }^{\pi } \ln (Re^{i\varphi }) \frac{d \varphi }{2\pi } = \ln R, \\&\int _{-\pi }^{\pi } \ln (Re^{i\varphi })Re^{i\varphi } \frac{d \varphi }{2\pi } = - R, \end{aligned}$$

this proves (7.14f).

Taking $j = 2$ in (C.3) and utilizing the fact that

$$\begin{aligned}&\int _{-\pi }^{\pi } \ln (Re^{i\varphi })R^{2}e^{2i\varphi } \frac{d \varphi }{2\pi } = \frac{R^{2}}{2}, \end{aligned}$$

we find

C.3 $$\begin{aligned} 2\int _{\gamma _{b_2b_1}} \frac{\zeta ^2 \ln {\zeta }}{r(\zeta )} \frac{d\zeta }{2\pi i} =&\; \lim _{R \rightarrow \infty } \bigg \{\int _{-\pi }^{\pi } R^2e^{2i \varphi } \ln (Re^{i\varphi })\bigg (\frac{1}{Re^{i\varphi }} + \frac{b_1 + b_2}{2 R^2 e^{2i \varphi }} \nonumber \\&+ \frac{3 b_1^2+2 b_1 b_2+3 b_2^2}{8 R^3 e^{3i\varphi }} \bigg ) \frac{iRe^{i\varphi } d\varphi }{2\pi i} + \int _{-R}^0 \frac{\zeta ^2 }{r(\zeta )} d\zeta \bigg \} \nonumber \\ =&\; \lim _{R \rightarrow \infty } \bigg \{\frac{R^2}{2} - \frac{b_1 + b_2}{2} R + \frac{(\mathrm{Re}\,{b_2})^2 - 2(\mathrm{Im}\,{b_2})^2}{2}\ln {R} + \int _{-R}^0 \frac{\zeta ^2 }{r(\zeta )} d\zeta \bigg \}. \end{aligned}$$

With the help of the identity

$$\begin{aligned} \frac{d}{d\zeta } \bigg \{\frac{3b_1 + 2\zeta + 3 b_2}{4}r(\zeta ) + \frac{3b_1^2 + 2b_1 b_2 + 3b_2^2}{8} \ln (-2 r(\zeta ) (1 + r'(\zeta )))\bigg \} = \frac{\zeta ^2}{r(\zeta )}, \end{aligned}$$

we infer the following large *R* asymptotics:

$$\begin{aligned} \int _{-R}^0 \frac{\zeta ^2}{r(\zeta )} d\zeta =&\; \bigg [\frac{3(b_1 + b_2) + 2\zeta }{4}r(\zeta ) + \frac{3b_1^2 + 2b_1 b_2 + 3b_2^2}{8} \ln (-2 r(\zeta ) (1 + r'(\zeta )))\bigg ]_{\zeta = -R}^0 \\ =&-\frac{R^2}{2} + i\mathrm{Im}\,(b_2) R + \frac{2(\mathrm{Im}\,{b_2})^2 - (\mathrm{Re}\,{b_2})^2}{2} \ln {R} \\&+\frac{1}{4} \bigg (2 \left( (\mathrm{Re}\,{b_1})^2-2 (\mathrm{Im}\,{b_2})^2\right) \ln (2 i (|b_2|+\mathrm{Im}\,(b_2)))+6 |b_2| \mathrm{Im}\,(b_2) \\&+(\mathrm{Im}\,{b_2})^2 (8\ln (2)-6)+(\mathrm{Re}\,{b_1})^2 (1-4 \ln (2))\bigg ) + {{{\mathcal {O}}}}(R^{-1}). \end{aligned}$$

Substituting the above expansion into (C.4), we obtain (7.14g).

If $j \le -1$ in (C.2), we have

$$\begin{aligned} 2\int _{\gamma _{b_2b_1}} \frac{\zeta ^{j}\ln {\zeta }}{ r(\zeta )} \frac{d\zeta }{2\pi i} =&\; \int _{\sigma } \frac{\ln {\zeta }}{\zeta ^{|j|} r(\zeta )} \frac{d\zeta }{2\pi i} \\ =&\; \int _{C_{R}} \frac{\ln {\zeta }}{\zeta ^{|j|} r(\zeta )} \frac{d\zeta }{2\pi i} + \int _{-R}^{-\epsilon } \frac{2\pi i}{\zeta ^{|j|} r(\zeta )} \frac{d\zeta }{2\pi i} - \int _{C_{\epsilon }} \frac{\ln {\zeta }}{\zeta ^{|j|} r(\zeta )} \frac{d\zeta }{2\pi i} \\ =&\; \int _{-\pi }^{\pi } \frac{\ln (Re^{i\varphi })}{R^{|j|}e^{i |j| \varphi }} \bigg (\frac{1}{Re^{i\varphi }} + \frac{b_1 + b_2}{2 R^2 e^{2i \varphi }} + {{{\mathcal {O}}}}(R^{-3})\bigg ) \frac{iRe^{i\varphi } d\varphi }{2\pi i} \\&+ \int _{-R}^{-\epsilon } \frac{d\zeta }{\zeta ^{|j|} r(\zeta )} - \int _{-\pi }^\pi \frac{\ln (\epsilon e^{i\varphi })}{\epsilon ^{|j|}e^{i |j| \varphi }}\bigg (\frac{i}{|b_2|} + \frac{\mathrm{Im}\,{b_2}}{|b_2|^3} \epsilon e^{i\varphi } + {{{\mathcal {O}}}}(\epsilon ^2)\bigg ) \frac{i\epsilon e^{i\varphi } d\varphi }{2\pi i}, \end{aligned}$$

as $R \rightarrow + \infty$ and $\epsilon \rightarrow 0^{+}$. Taking the limit $R \rightarrow + \infty$, and then the limit $\epsilon \rightarrow 0^{+}$ gives

$$\begin{aligned}&2\int _{\gamma _{b_2b_1}} \frac{\zeta ^{j}\ln {\zeta }}{ r(\zeta )} \frac{d\zeta }{2\pi i} \\&\quad = \lim _{\epsilon \rightarrow 0^{+}} \bigg \{ \int _{-\infty }^{-\epsilon } \frac{d\zeta }{\zeta ^{|j|} r(\zeta )} - \int _{-\pi }^\pi \frac{\ln (\epsilon e^{i\varphi })}{\epsilon ^{|j|}e^{i |j| \varphi }}\bigg (\frac{i}{|b_2|} + \frac{\mathrm{Im}\,{b_2}}{|b_2|^3} \epsilon e^{i\varphi } + {{{\mathcal {O}}}}(\epsilon ^2)\bigg ) \frac{i\epsilon e^{i\varphi } d\varphi }{2\pi i} \bigg \}. \end{aligned}$$

Suppose now that $j = -1$. Using that

$$\begin{aligned} \frac{d}{d\zeta } \frac{\ln \bigg (\frac{i(b_2 - \zeta ) - \frac{b_2}{|b_2|} r(\zeta )}{i(b_2 - \zeta ) + \frac{b_2}{|b_2|} r(\zeta )}\bigg )}{i|b_2|} = \frac{1}{\zeta r(\zeta )}, \end{aligned}$$

we find that

$$\begin{aligned} \int _{-R}^{-\epsilon } \frac{d\zeta }{\zeta r(\zeta )}&= \frac{\ln \bigg (\frac{i(b_2 - \zeta ) - \frac{b_2}{|b_2|} r(\zeta )}{i(b_2 - \zeta ) + \frac{b_2}{|b_2|} r(\zeta )}\bigg )}{i|b_2|}\bigg |_{-\infty }^{-\epsilon } \\&= \frac{i \ln {\epsilon }}{|b_2|} + \frac{\ln (\frac{2|b_2|^2}{\mathrm{Re}\,{b_2}})}{i|b_2|} - \frac{\ln (-\frac{i(|b_2| + \mathrm{Im}\,{b_2})}{\mathrm{Re}\,{b_2}})}{i|b_2|} + {{{\mathcal {O}}}}(\epsilon ) \end{aligned}$$

as $\epsilon \rightarrow 0^{+}$. Also, by a straightforward computation,

$$\begin{aligned}&\int _{-\pi }^\pi \frac{\ln (\epsilon e^{i\varphi })}{\epsilon e^{i \varphi }}\bigg (\frac{1}{-i|b_2|} + \frac{\mathrm{Im}\,{b_2}}{|b_2|^3} \epsilon e^{i\varphi } + \cdots \bigg ) \frac{i\epsilon e^{i\varphi } d\varphi }{2\pi i} = \frac{i \ln {\epsilon }}{|b_2|} + {{{\mathcal {O}}}}(\epsilon ) \end{aligned}$$

as $$\epsilon \rightarrow 0^{+}$$. Hence

$$\begin{aligned} 2\int _{\gamma _{b_2b_1}} \frac{\ln {\zeta }}{\zeta r(\zeta )} \frac{d\zeta }{2\pi i} =&\; \frac{\ln (\frac{2|b_2|^2}{\mathrm{Re}\,{b_2}})}{i|b_2|} - \frac{\ln (-\frac{i(|b_2| + \mathrm{Im}\,{b_2})}{\mathrm{Re}\,{b_2}})}{i|b_2|} = \frac{\ln (\frac{2i|b_2|^2}{|b_2| + \mathrm{Im}\,{b_2}})}{i|b_2|}, \end{aligned}$$

which proves the eighth and last identity (7.14h). The proof of Lemma 7.2 is complete.

## Proof of Lemma 8.4

Suppose $\alpha > -1$ and $\theta \in (0,1]$. Defining $f_+(\zeta )$ and $f_-(\zeta )$ by

$$\begin{aligned}&f_\pm (\zeta ) = \ln (\pm i\zeta ) \pm \ln \bigg (\frac{|b_2|^2 + i\zeta \mathrm{Im}\,{b_2} - i|b_2| r(\zeta )}{(\pm r(\zeta ) + \zeta - i\mathrm{Im}\,{b_2})\zeta }\bigg ), \end{aligned}$$

the definition (8.25) of ${\mathcal {B}}(\zeta )$ can be written as

C.4 $$\begin{aligned} {\mathcal {B}}(\zeta ) = -c_5 f_+(\zeta ) - c_6 f_-(\zeta ) - c_{7}. \end{aligned}$$

Thus,

D.1 $$\begin{aligned} \int _{\gamma _{b_2b_1}} {\mathcal {B}}(\zeta ) d\zeta = - c_5 \int _{\gamma _{b_2b_1}} f_+(\zeta ) d\zeta - c_6 \int _{\gamma _{b_2b_1}} f_-(\zeta ) d\zeta - c_7 (b_1 - b_2). \end{aligned}$$

Integrating by parts and using that

D.2 $$\begin{aligned} f_\pm '(\zeta ) = \frac{1}{\zeta } - \frac{1}{r(\zeta )} \mp \frac{i|b_2|}{\zeta r(\zeta )}, \end{aligned}$$

we find

$$\begin{aligned} \int _{\gamma _{b_2b_1}} f_\pm (\zeta ) d\zeta = b_1 f_\pm (b_1) - b_2 f_\pm (b_2) - \int _{\gamma _{b_2b_1}} \bigg (1 - \frac{\zeta }{r(\zeta )} \mp \frac{i|b_2|}{r(\zeta )}\bigg ) d\zeta , \end{aligned}$$

Substituting these expressions into (D.2) and using (7.14a) and (7.14b), the first assertion (8.27a) of the lemma follows after simplification.

To prove (8.27b), we use (D.1) to write

D.3 $$\begin{aligned}&\int _{\gamma _{b_2b_1}} \ln (\zeta ) {\mathcal {B}}(\zeta ) d\zeta \nonumber \\&\quad = - c_5 \int _{\gamma _{b_2b_1}} \ln (\zeta ) f_+(\zeta ) d\zeta - c_6 \int _{\gamma _{b_2b_1}} \ln (\zeta ) f_-(\zeta ) d\zeta - c_7 \int _{\gamma _{b_2b_1}} \ln ( \zeta ) d\zeta . \end{aligned}$$

Employing (D.3) and the fact that

$$\begin{aligned} \int \ln {\zeta } d\zeta = \zeta (\ln (\zeta ) - 1), \end{aligned}$$

partial integration gives

$$\begin{aligned}&\int _{\gamma _{b_2b_1}} \ln (\zeta ) {\mathcal {B}}(\zeta ) d\zeta \\&\quad = - c_5 \bigg \{\Big [ \zeta (\ln (\zeta ) - 1) f_+(\zeta )\Big ]_{\zeta =b_2}^{b_1} - \int _{\gamma _{b_2b_1}} (\ln (\zeta ) - 1) \bigg (1 - \frac{\zeta }{r(\zeta )} - \frac{i|b_2|}{r(\zeta )}\bigg )d\zeta \bigg \}\\&\qquad - c_6\bigg \{\Big [\zeta (\ln (\zeta ) - 1) f_-(\zeta )\Big ]_{\zeta =b_2}^{b_1} - \int _{\gamma _{b_2b_1}} (\ln (\zeta ) - 1) \bigg (1 - \frac{\zeta }{r(\zeta )} + \frac{i|b_2|}{r(\zeta )}\bigg )d\zeta \bigg \}\\&\qquad - c_7 \int _{\gamma _{b_2b_1}} \ln (\zeta ) d\zeta , \end{aligned}$$

that is,

D.4 $$\begin{aligned} \int _{\gamma _{b_2b_1}} \ln (\zeta ) {\mathcal {B}}(\zeta ) d\zeta =&- c_5 \Big [ \zeta (\ln (\zeta ) - 1) f_+(\zeta )\Big ]_{\zeta =b_2}^{b_1} - c_6\Big [\zeta (\ln (\zeta ) - 1) f_-(\zeta )\Big ]_{\zeta =b_2}^{b_1} \nonumber \\&+ (c_5 + c_6) \int _{\gamma _{b_2b_1}} (\ln (\zeta ) - 1) d\zeta -(c_5 + c_6) \int _{\gamma _{b_2b_1}} \frac{\zeta \ln (\zeta )}{r(\zeta )} d\zeta \nonumber \\&+ (c_5 + c_6) \int _{\gamma _{b_2b_1}} \frac{\zeta }{r(\zeta )} d\zeta - (c_5 - c_6) i|b_2| \int _{\gamma _{b_2b_1}} \frac{\ln (\zeta )}{r(\zeta )}d\zeta \nonumber \\&+ (c_5 - c_6) i|b_2| \int _{\gamma _{b_2b_1}} \frac{1}{r(\zeta )}d\zeta - c_7 \int _{\gamma _{b_2b_1}} \ln (\zeta ) d\zeta . \end{aligned}$$

An easy computation shows that

$$\begin{aligned} \int _{\gamma _{b_2b_1}} \ln (\zeta ) d\zeta = b_2 - b_1 + b_1 \ln (b_1) - b_2 \ln (b_2) \end{aligned}$$

and explicit expressions for the other integrals on the right-hand side of (D.5) have already been obtained in (7.14f), (7.14b), (7.14e), and (7.14a). Substituting these expressions into (D.5), a long but straightforward simplification gives (8.27b).

We next prove (8.27c). According to the definition (8.24) of ${\mathcal {A}}$, we have

D.5 $$\begin{aligned} \int _{\gamma _{b_2b_1}} {\mathcal {A}}(\zeta ) d\zeta = \frac{ic_8}{2}\int _{\gamma _{b_2b_1}} \frac{d\zeta }{\zeta } + \frac{c_8-\frac{3\alpha ^2-1}{12}}{2|b_2|} \int _{\gamma _{b_2b_1}} \frac{r(\zeta )}{\zeta }d\zeta . \end{aligned}$$

Clearly,

D.6 $$\begin{aligned} \int _{\gamma _{b_{2}b_{1}}} \frac{d\zeta }{\zeta } = \ln b_{1} - \ln b_{2} = i(\arg b_{1} - \arg b_{2}), \end{aligned}$$

so it only remains to compute $\int _{\gamma _{b_2b_1}} \frac{r(\zeta )}{\zeta }d\zeta$. Let ${\tilde{r}}(\zeta )$ denote the analytic continuation of $r(\zeta )$ defined in (7.4). For $r > 0$, let $C_r$ denote the positively oriented circle of radius *r* centered at the origin. A contour deformation shows that

D.7 $$\begin{aligned} \int _{\gamma _{b_{2}b_{1}}}\frac{r(\zeta )}{ \zeta }d\zeta = \int _{\gamma _{b_{2}b_{1}}}\frac{{\tilde{r}}(\zeta )}{ \zeta }d\zeta = \frac{1}{2} \int _{C_{R}}\frac{{\tilde{r}}(\zeta )}{ \zeta }d\zeta - \frac{1}{2}\int _{C_{\epsilon }}\frac{{\tilde{r}}(\zeta )}{ \zeta }d\zeta , \end{aligned}$$

where $R>|b_{1}|$ and $0< \epsilon < \mathrm{Im}\,b_2$. Using the expansions

$$\begin{aligned}&\frac{{\tilde{r}}(\zeta )}{\zeta } = 1 - \frac{b_{1}+b_{2}}{2\zeta } + {{{\mathcal {O}}}}(\zeta ^{-2}), \qquad \zeta \rightarrow \infty , \\&\frac{{\tilde{r}}(\zeta )}{\zeta } = - \frac{i |b_{2}|}{\zeta } + {{{\mathcal {O}}}}(1), \qquad \zeta \rightarrow 0, \end{aligned}$$

and letting $R \rightarrow + \infty$ and $\epsilon \rightarrow 0^{+}$ in (D.8), we infer that

D.8 $$\begin{aligned} \int _{\gamma _{b_{2}b_{1}}}\frac{r(\zeta )}{ \zeta }d\zeta = - \pi \big ( |b_{2}|-\mathrm{Im}\,b_{2} \big ). \end{aligned}$$

Substituting (D.7) and (D.9) into (D.6) and simplifying, we find (8.27c).

To prove (8.27d), we use the definition (8.24) of ${\mathcal {A}}$ to write

D.9 $$\begin{aligned} \int _{\gamma _{b_{2}b_{1}}} \ln ( \zeta ) {\mathcal {A}}(\zeta )d\zeta = \frac{ic_{8}}{2}\int _{\gamma _{b_{2}b_{1}}} \frac{\ln (\zeta )}{\zeta }d\zeta + \frac{c_8-\frac{3\alpha ^2-1}{12}}{2|b_2|}\int _{\gamma _{b_{2}b_{1}}} \frac{\ln (\zeta ) r(\zeta )}{\zeta }d\zeta .\nonumber \\ \end{aligned}$$

The first integral on the right-hand side is easily computed:

D.10 $$\begin{aligned} \int _{\gamma _{b_2b_1}} \frac{\ln {\zeta }}{\zeta } d\zeta = \frac{(\ln {b_1})^2 - (\ln {b_2})^2}{2}. \end{aligned}$$

To compute the second integral, we use a contour deformation to obtain

D.11 $$\begin{aligned} \int _{\gamma _{b_2b_1}} r(\zeta )\frac{\ln \zeta }{\zeta }d\zeta&= \frac{1}{2}\int _{C_{R}}\frac{{\tilde{r}}(\zeta ) \ln \zeta }{ \zeta }d\zeta - \frac{1}{2}\int _{C_{\epsilon }}\frac{{\tilde{r}}(\zeta )\ln \zeta }{ \zeta }d\zeta + \pi i \int _{-R}^{-\epsilon } \frac{{\tilde{r}}(\zeta )}{\zeta }d\zeta ,\nonumber \\&= \frac{i}{2}\int _{-\pi }^{\pi } {\tilde{r}}(Re^{i\varphi })\ln (Re^{i\varphi })d\varphi - \frac{i}{2}\int _{-\pi }^{\pi } {\tilde{r}}(\epsilon e^{i\varphi })\ln (\epsilon e^{i\varphi })d\varphi \nonumber \\&\qquad + \pi i \int _{-R}^{-\epsilon } \frac{{\tilde{r}}(\zeta )}{\zeta }d\zeta . \end{aligned}$$

where *R* and $\epsilon$ are as in (D.8). Since

$$\begin{aligned}&\partial _{\zeta } \Bigg [ r(\zeta ) + i |b_{2}| \ln \left( \frac{b_{2}r(\zeta )+i|b_{2}|(\zeta -b_{2})}{-b_{2} r(\zeta )+i|b_{2}|(\zeta -b_{2})} \right) - \frac{b_{1}+b_{2}}{2}\ln \big ( 2r(\zeta ) ( 1+r'(\zeta )) \big ) \Bigg ] \\&\quad = \frac{r(\zeta )}{\zeta }, \end{aligned}$$

we have

$$\begin{aligned} \int _{-R}^{-\epsilon } \frac{{\tilde{r}}(\zeta )}{\zeta }d\zeta =&- i \sqrt{- b_{1}-\epsilon }\sqrt{b_{2} + \epsilon } + i |b_{2}| \ln \left( \frac{|b_{2}| \sqrt{b_{2}+\epsilon } + b_{2} \sqrt{-b_{1}-\epsilon }}{|b_{2}| \sqrt{b_{2}+\epsilon } - b_{2} \sqrt{-b_{1}-\epsilon }} \right) \\&- i \mathrm{Im}\,(b_{2}) \left[ \ln \left( 2\epsilon + 2 i \mathrm{Im}\,(b_{2}) + 2i \sqrt{- b_{1}-\epsilon }\sqrt{b_{2} + \epsilon } \right) -\pi i \right] \\&+ \sqrt{b_{1}+R}\sqrt{b_{2}+R} - i \mathrm{Im}\,(b_{2}) \ln \left( \frac{i |b_{2}| \sqrt{b_{2}+R} + b_{2} \sqrt{b_{1}+R}}{i |b_{2}| \sqrt{b_{2}+R} - b_{2} \sqrt{b_{1}+R}} \right) \\&+i \mathrm{Im}\,(b_{2}) \left[ \ln \left( 2i \mathrm{Im}\,(b_{2})+2R+2\sqrt{b_{1}+R}\sqrt{b_{2}+R} \right) -\pi i \right] . \end{aligned}$$

Substituting this into (D.12) and letting $R \rightarrow + \infty$ and $\epsilon \rightarrow 0^{+}$ in the resulting equation, we find

D.12 $$\begin{aligned} \int _{\gamma _{b_2b_1}} r(\zeta )\frac{\ln \zeta }{\zeta }d\zeta =&\; \frac{i}{2}\int _{-\pi }^{\pi } \Big ( Re^{i\varphi }- \frac{b_{1}+b_{2}}{2} + {{{\mathcal {O}}}}(R^{-1}) \Big )\ln (Re^{i\varphi })d\varphi \nonumber \\&-\frac{i}{2} \int _{-\pi }^{\pi } \Big ( -i|b_{2}| + {{{\mathcal {O}}}}(\epsilon ) \Big ) \ln (\epsilon e^{i\varphi }) d\varphi +\pi i \int _{-R}^{-\epsilon } \frac{{\tilde{r}}(\zeta )}{\zeta }d\zeta \nonumber \\ =&\; \pi \bigg \{|b_2| - |b_2| \ln \bigg (\frac{2i|b_2|^2}{|b_2| + \mathrm{Im}\,{b_2}}\bigg ) - \mathrm{Im}\,{b_2} + \mathrm{Im}\,(b_2)\ln \bigg (\frac{i(|b_2| + \mathrm{Im}\,{b_2})}{2}\bigg )\bigg \}. \end{aligned}$$

Substituting of (D.11) and (D.13) into (D.10), we obtain (8.27d).

We finally prove (8.27e). By (D.1), we have

D.13 $$\begin{aligned} \int _{\gamma _{b_2b_1}} \frac{{\mathcal {B}}(\zeta )}{\zeta } d\zeta = - c_5 \int _{\gamma _{b_2b_1}} \frac{f_+(\zeta )}{\zeta } d\zeta - c_6 \int _{\gamma _{b_2b_1}} \frac{f_-(\zeta )}{\zeta } d\zeta - c_7 \int _{\gamma _{b_2b_1}} \frac{d\zeta }{\zeta }. \end{aligned}$$

Integration by parts using (D.3) gives

$$\begin{aligned} \int _{\gamma _{b_2b_1}} \frac{{\mathcal {B}}(\zeta )}{\zeta } d\zeta =&- c_5 \bigg \{\Big [ \ln (\zeta ) f_+(\zeta )\Big ]_{\zeta =b_2}^{b_1} - \int _{\gamma _{b_2b_1}} \ln (\zeta ) \bigg (\frac{1}{\zeta } - \frac{1}{r(\zeta )} - \frac{i|b_2|}{\zeta r(\zeta )}\bigg )d\zeta \bigg \}\\&- c_6\bigg \{\Big [\ln (\zeta ) f_-(\zeta )\Big ]_{\zeta =b_2}^{b_1} - \int _{\gamma _{b_2b_1}} \ln (\zeta ) \bigg (\frac{1}{\zeta } - \frac{1}{r(\zeta )} + \frac{i|b_2|}{\zeta r(\zeta )}\bigg )d\zeta \bigg \} \\&- c_7 \int _{\gamma _{b_2b_1}} \frac{d\zeta }{\zeta }, \end{aligned}$$

i.e.,

D.14 $$\begin{aligned} \int _{\gamma _{b_2b_1}} \frac{{\mathcal {B}}(\zeta )}{\zeta } d\zeta =&- c_5\Big [ \ln (\zeta ) f_+(\zeta )\Big ]_{\zeta =b_2}^{b_1} - c_6\Big [\ln (\zeta ) f_-(\zeta )\Big ]_{\zeta =b_2}^{b_1} +(c_5 + c_6) \int _{\gamma _{b_2b_1}} \frac{\ln (\zeta )}{\zeta } d\zeta \nonumber \\&- (c_5 + c_6) \int _{\gamma _{b_2b_1}} \frac{\ln (\zeta )}{r(\zeta )} d\zeta + i|b_2| (c_6- c_5) \int _{\gamma _{b_2b_1}} \frac{\ln (\zeta )}{\zeta r(\zeta )} d\zeta - c_7 \int _{\gamma _{b_2b_1}} \frac{d\zeta }{\zeta }. \end{aligned}$$

The four integrals on the right-hand side of (D.15) have been computed in (D.11), (7.14e), (7.14h), and (D.7), respectively. Substituting the expressions from these equations into (D.15) and simplifying, (8.27e) follows. This completes the proof of Lemma 8.4.

## Footnotes

1 Note that, due to the re-scaling (1.5), $\left. \det \big ( 1-\left. {\mathbb {K}}\right| _{[0,s]} \big )\right| _{\theta = 1} = \det \big ( \left. 1-{\mathbb {K}}_{\mathrm {Be}} \right| _{[0,4s]} \big )$, and thus one should use [15, Theorem 4] with *s* replaced by 4*s* to obtain (1.7).

2 Note that $d(\theta ,\alpha )$ is well-defined for $\alpha = -1$ even though the point process is defined only for $\theta > 0$ and $\alpha > -1$.

3 From the analysis of [12], we can show that the error term in (1.8) is indeed differentiable and satisfies $\partial _{s} {{{\mathcal {O}}}}(s^{-\rho }) = {{{\mathcal {O}}}}(s^{-\rho -1})$ and $\partial _{\theta } {{{\mathcal {O}}}}(s^{-\rho }) = {{{\mathcal {O}}}}(s^{-\rho } \ln s)$ as $s \rightarrow + \infty$.

4 Here we have corrected a small typo in [12, equation (3.12)] in the expression for $c_{8}$, which has no consequence for the results of [12] as $c_{8}$ does not play any role in the computation of *a* and *b*.

5 Note that the conjugation by $e^{p_{0}\sigma _{3}}$ only affects the off-diagonal elements of *R*. Thus, even though this conjugation was not present in [12], this does not affect the results of that paper as they only depend on the (2, 2) element of *R*.

6 Note that there is a typo in the lemma: it should be $2g(\zeta ) - i h(\zeta ) + \ell$ instead of $2g(\zeta ) - i h(\zeta ) - \ell$. Existence of a constant $c'$ is clear from the proof of the lemma.

7 ${\mathbb {K}}$ in the present paper is denoted by ${\mathbb {K}}^{(3)}$ in [12].

8 The quantities $\rho$, *a*, *b*, *c* and *C* in the present paper are denoted by $\rho ^{(3)}$, $a^{(3)}$, $b^{(3)}$, $c^{(3)}$ and $C^{(3)}$ in [12].

9 The Riemann zeta function $\zeta (\cdot )$ should not be confused with the complex variable $\zeta$ introduced in (2.2).
